# A Generative Angular Model of Protein Structure Evolution

**DOI:** 10.1093/molbev/msx137

**Published:** 2017-04-27

**Authors:** Michael Golden, Eduardo García-Portugués, Michael Sørensen, Kanti V. Mardia, Thomas Hamelryck, Jotun Hein

**Affiliations:** 1 Department of Statistics, University of Oxford, Oxford, United Kingdom; 2 Department of Statistics, Carlos III University of Madrid, Madrid, Spain; 3 Department of Mathematical Sciences, University of Copenhagen, Copenhagen, Denmark; 4 Department of Mathematics, University of Leeds, Leeds, United Kingdom; 5 Bioinformatics Centre, Section for Computational and RNA Biology, Department of Biology, University of Copenhagen, Copenhagen, Denmark; 6 Image Section, Department of Computer Science, University of Copenhagen, Copenhagen, Denmark

**Keywords:** evolution, protein structure, probabilistic model, directional statistics

## Abstract

Recently described stochastic models of protein evolution have demonstrated that the inclusion of structural information in addition to amino acid sequences leads to a more reliable estimation of evolutionary parameters. We present a generative, evolutionary model of protein structure and sequence that is valid on a local length scale. The model concerns the local dependencies between sequence and structure evolution in a pair of homologous proteins. The evolutionary trajectory between the two structures in the protein pair is treated as a random walk in dihedral angle space, which is modeled using a novel angular diffusion process on the two-dimensional torus. Coupling sequence and structure evolution in our model allows for modeling both “smooth” conformational changes and “catastrophic” conformational jumps, conditioned on the amino acid changes. The model has interpretable parameters and is comparatively more realistic than previous stochastic models, providing new insights into the relationship between sequence and structure evolution. For example, using the trained model we were able to identify an apparent sequence–structure evolutionary motif present in a large number of homologous protein pairs. The generative nature of our model enables us to evaluate its validity and its ability to simulate aspects of protein evolution conditioned on an amino acid sequence, a related amino acid sequence, a related structure or any combination thereof.

## Introduction

Recently, several studies (Challis and Schmidler 2012; Herman et al. 2014) have proposed joint stochastic models of evolution which take into account simultaneous alignment of protein sequence and structure. These studies point out the limitations of earlier non-probabilistic methods, which often rely on heuristic procedures to infer parameters of interest. A major disadvantage of using heuristic procedures is that they typically fail to account for sources of uncertainty. For example, relying on a single fixed alignment, which is highly unlikely to be the *true* underlying alignment, may bias the inference of the posterior distribution over evolutionary trees.

We present a generative evolutionary model, ETDBN (Evolutionary Torus Dynamic Bayesian Network) for pairs of homologous proteins. ETDBN captures dependencies between sequence and structure evolution, accounts for alignment uncertainty, and models the local dependencies between aligned sites.

A key step in modeling protein structure evolution is selecting a suitable structural representation and corresponding evolutionary model. Early works by Gutin and Badretdinov (1994) and Grishin (1997) represented protein structure using three-dimensional Cartesian coordinates of protein backbone atoms and used diffusions processes to model the relationship between structural distance (measured using RMSD) and sequence similarity. More recent publications by Challis and Schmidler (2012) and Herman et al. (2014) likewise used the three-dimensional Cartesian coordinates of amino acid C_*α*_ atoms to represent protein structure and additionally used Ornstein-Uhlenbeck (OU) processes to construct Bayesian probabilistic models of protein structure evolution. These models emphasize estimation of evolutionary parameters such as the evolutionary time between species, tree topologies and alignment, and attempt to fully account for sources of uncertainty. For the sake of computational tractability, the aforementioned approaches treat the Cartesian coordinates associated with atoms as evolving independently of another. A non-probabilistic approach by Echave (2008) and Echave and Fernández (2010) referred to as the Linearly Forced Elastic Network Model (LFENM) treats protein structures as a collection of C_*α*_ atoms connected by spring forces. The major benefit of LFENMs is that they do not assume independence of atomic coordinates and take into account non-local dependencies due to physical interactions. In their current formulation LFENMs do not distinguish between the differing chemical nature of different amino acids and therefore do not account for the variable effect of sequence mutation on protein structure evolution.

**Fig. 1:**
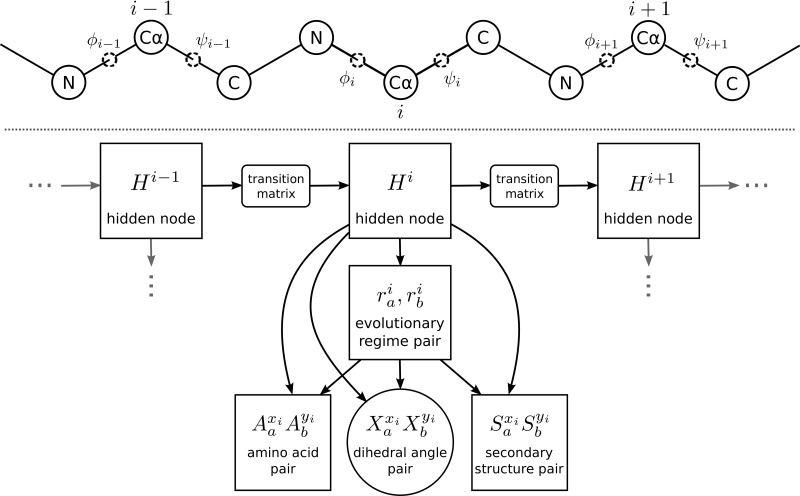
Above: dihedral angle representation. A small section of a single protein backbone (three amino acids) with φ and *ψ* dihedral angles shown, together with C_*α*_ atoms which attach to the amino acid side-chains. Each amino acid side-chain determines the characteristic nature of each amino acid. Every amino acid position corresponds to a hidden node in the HMM below. Note that we only show a single protein, whereas the model considers a pair. Below: depiction of HMM architecture of ETDBN where each *H* along the horizontal axis represents an evolutionary hidden node. The horizontal edges between evolutionary hidden nodes encode neighboring dependencies between aligned sites. The arrows between the evolutionary hidden nodes and site-class pair nodes encode the conditional independence between the observation pair variables $A_{a}^{x_{i}},A_{b}^{y_{i}}$ (amino acid site pair), $X_{a}^{x_{i}}=\langle \varphi_{a}^{x_{i}},\psi_{a}^{x_{i}}\rangle ,X_{b}^{y_{i}}=\langle \varphi_{b}^{y_{i}},\psi_{b}^{y_{i}}\rangle$ (dihedral angle site pair) and $S_{a}^{x_{i}},S_{b}^{y_{i}}$ (secondary structure class site pair). The circles represent continuous variables and the rectangles represent discrete variables.

Rather than using a Cartesian coordinate representation, our model, ETDBN, uses a dihedral angle representation motivated by the non-evolutionary TorusDBN model (Boomsma et al. 2008, 2014). TorusDBN represents a single protein structure as a sequence of (φ,ψ) dihedral angle pairs, which are modeled using continuous bivariate angular distributions (Frellsen et al. 2012). Likewise, ETDBN treats protein structure as a random walk in space, again making use of the φ and *ψ* dihedral angles (top of fig. 1).

The dihedral angle representation is informed by the chemical nature of peptide bonds. Each amino acid in a protein peptide chain is covalently bonded to the next via a peptide bond. Peptide bonds have a partial double bond nature that results in a planar configuration of atoms in space. This configuration allows the protein backbone structure to be largely described in terms of a series of φ and *ψ* dihedral angles that defines the relationship between the planes in three-dimensional space. A benefit of this representation is that it bypasses the need for structural alignment, unlike in models on Cartesian coordinates which typically need to additionally superimpose the structures for comparison purposes (Herman et al. 2014). Accordingly, having to account for superimposition introduces an additional source of uncertainty. A further advantage of the dihedral angle representation is that there are fewer degrees of freedom per amino acid and therefore typically fewer parameters required in order to model their evolution.

The evolution of dihedral angles in ETDBN is modeled using a novel stochastic diffusion process developed in García-Portugués et al. (2017). In addition to this, a coupling is introduced such that an amino acid change can lead to a *jump* in dihedral angles and a change in diffusion process, allowing the model to capture changes in amino acid that are directionally coupled with changes in dihedral angle or secondary structure. As in Challis and Schmidler (2012) and Herman et al. (2014), the insertion and deletion (indel) evolutionary process is also modeled in order to account for alignment uncertainty (Thorne et al. 1992).

The OU processes used in Challis and Schmidler (2012) and Herman et al. (2014) ignore bond lengths and treat C_*α*_ atoms as evolving independently for the sake of computationally tractability. Furthermore, the OU process makes Gaussian assumptions. From a generative perspective these properties will lead to evolved proteins with C_*α*_ atoms that are unnaturally dispersed in space. Bond lengths are also ignored in ETDBN, but can be plausibly fixed or modeled. As a result, it is expected that the use of angular diffusions will much more naturally capture the underlying protein structure manifold.

Two or more homologous proteins will share a common ancestor, which leads to underlying tree-like dependencies. These dependencies manifest themselves most noticeably in the degree of amino acid sequence similarity between two homologous proteins. The strength of these dependencies is assumed to be a result of two major factors: the time since the common ancestor and the rate of evolution.

Failing to account for evolutionary dependencies can lead to false conclusions (Felsenstein 1985), whereas accounting for evolutionary dependencies allows information from homologous proteins to be incorporated in a principled manner. This can lead to more accurate inferences, such as the prediction of a protein structure from a homologous protein sequence and structure, known as homology modeling (Arnold et al. 2006). Stochastic models such as ETDBN are not expected to compete with homology modeling software such as SWISS-MODEL (Arnold et al. 2006). However, they allow for estimation of evolutionary parameters and statements about uncertainty to be made in a statistically rigorous manner.

Most models of structural evolution ignore dependencies amongst sites because of the increased computational demand and complexity associated with such models. These dependencies are expected to influence patterns of evolution, specifically patterns of amino acid substitution. The current model deals with local dependencies only—dependencies that are expected to arise due to interactions between neighboring amino acids, for example, between amino acids in an *α*-helix. ETDBN does not account for global dependencies—dependencies that result in the globular nature of proteins (Boomsma et al. 2008). In ETDBN, we attempt to model local dependencies only by using a Hidden Markov Model (HMM) to capture dependencies amongst neighboring aligned positions. HMMs such as PASSML (Liò et al. 1998) have been successfully used to predict protein secondary structure from aligned sequences, however, these models typically have the disadvantage that they assume a canonical secondary structure shared amongst all the sequences being analyzed. This restricts analysis to closely related sequences where conservation of secondary structure is a reasonable assumption. ETDBN does not assume a canonical secondary structure, but instead uses a phylogenetic HMM approach, similar to Siepel and Haussler (2004), that assumes dependencies between evolutionary processes at neighboring aligned positions.

Parameters of ETDBN were estimated using 1,200 homologous protein pairs from the HOMSTRAD database (Mizuguchi et al. 1998). The resulting model provides a realistic prior distribution over proteins and protein structure evolution in comparison to previous stochastic models. Doing so enables biological insights into the relationship between sequence and structure evolution, such as patterns of amino acid change that are informative of patterns of structural change (Grishin 2001). It was with these features in mind that ETDBN was developed.

## Evolutionary Model

### Overview

ETDBN is a dynamic Bayesian network model of local protein sequence and structure evolution along a pair of aligned homologous proteins *p_a_* and *p_b_*. ETDBN can be can be viewed as an HMM (see fig. 1). Each hidden node of the HMM, corresponding to an aligned position, adopts an evolutionary hidden state specifying a distribution over three different observations pairs: a pair of amino acid characters, a pair of dihedral angles and a pair of secondary structures classifications. A transition probability matrix specifies neighboring dependencies between adjacent evolutionary states. For example, transitions along the alignment between hidden states encoding predominantly *α*-helix evolution would be expected to occur more frequently than transitions between an evolutionary hidden state encoding predominantly *α*-helix evolution and another encoding predominantly *β*-sheet evolution.

Ideally, the underlying hidden states would not just vary across the length of the alignment as captured by the HMM in the current model, but also evolve along the branches of the phylogenetic tree. This remains computationally intractable at present. Allowing the hidden states to evolve along the tree would allow capturing large structural changes, even induced by a single mutation. For now we model such events using a jump model (see below).

Partially in order to mitigate this, each hidden state specifies a distribution over a pair of site-classes at each aligned position. This gives rise to the possibility of a ‘jump event’. A jump event allows a large change in dihedral angle or secondary structure (e.g. helix to sheet) to occur at a given aligned position and also introduces a directional coupling between changes in amino acid that are informative of changes in dihedral angle or secondary structure conformation.

### Observation Types

The two proteins, *p_a_* and *p_b_*, in a homologous pair are associated with a pair of observation sequences *O_a_* and *O_b_* obtained from experimental data, respectively. An *i*th site observation pair, $O^{i}=(O_{a}^{x(i)},O_{b}^{y(i)})$, is associated with every aligned site *i* in an alignment *M_ab_* of *p_a_* and *p_b_*, where $M_{ab}^{i}\in \left\{\left(\overset{x}{y}\right),\left(\overset{x}{-}\right),\left(\overset{-}{y}\right)\right\}$ specifies the homology relationship at position *i* of the alignment (homologous, deletion with respect to *p_a_* and insertion with respect to *p_a_*, respectively), *i* is taken to run from 1 to *m*, *m* is the length of the alignment *M_ab_*, and $x\in \{1,\ldots ,|p_{a}|\}$ and $y\in \{1,\ldots ,|p_{b}|\}$ specify the indices of the positions in *p_a_* and *p_b_*, respectively. |*p_a_*| and |*p_a_*| give the number of sites in *p_a_* and *p_b_*, respectively.

Each site observation, $O_{a}^{x(i)}$ and $O_{b}^{y(i)}$, contains amino acid and structural information corresponding to the two C_*α*_ atoms at aligned site *i* belonging to each of the two proteins. A site observation corresponding to a particular protein at aligned site *i*, $O_{a}^{x(i)}$, is comprised of three different data types associated with the C_*α*_ atom: an amino acid ($A_{a}^{x(i)}$; discrete, one of twenty canonical amino acids), φ and *ψ* dihedral angles ($X_{a}^{x(i)}=\langle \varphi_{a}^{x(i)},\psi_{a}^{x(i)}\rangle$; continuous, bivariate), and a secondary structure classification ($S_{a}^{x(i)}$; discrete, one of three classes: helix (H), sheet (S), or coil (C)). Therefore, $O_{a}^{x(i)}=(A_{a}^{x(i)},X_{a}^{x(i)},S_{a}^{x(i)})$ and $O_{b}^{y(i)}=(A_{b}^{y(i)},X_{b}^{y(i)},S_{b}^{y(i)})$.

### Model Structure

The sequence of hidden nodes in the HMM is written as $H=(H^{1},H^{2},\ldots ,H^{m}$). Each hidden node *H^i^* in the HMM corresponds to a site observation pair, $O_{a}^{x(i)}$ and $O_{b}^{y(i)}$, at an aligned site *i* in the alignment *M_ab_*. Initially we treat the alignment *M_ab_* as given a priori, but later modify the HMM to marginalize out an unobserved alignment.

The model is parameterized by *h* hidden states. Every hidden node *H^i^* corresponding to an aligned site *i* takes an integer value from 1 to *q* for the hidden state at node *H^i^*. In turn, each hidden state specifies a distribution over a site-class pair: $\left(r_{a}^{i},r_{b}^{i}\right)$ as a function of evolutionary time. A site-class pair consists of two site-classes: $r_{a}^{i}$ and $r_{b}^{i}$. Each of the two site-classes takes an integer value 1 or 2, that is, $\left(r_{a}^{i},r_{b}^{i})\in \{(1,1),(1,2),(2,1),(2,2)\right\}$. We return to the specific role of the site-classes pairs in the next section.

The state of *H^i^* together with the site-class pair, $\left(r_{a}^{i},r_{b}^{i}\right)$, and the evolutionary time separating proteins *p_a_* and *p_b_*, *t_ab_*, specify a distribution over three conditionally independent stochastic processes describing each of the three types of site observation pairs: $A^{i}=(A_{a}^{x(i)},A_{b}^{y(i)}),\;X^{i}=(X_{a}^{x(i)},X_{b}^{y(i)})$ and $S^{i}=(S_{a}^{x(i)},S_{b}^{y(i)})$. This conditional independence structure allows the likelihood of a site observation pair at an aligned site *i* to be written as follows:

(1) $$p(O^{i}|H^{i},r_{a}^{i},r_{b}^{i},t_{ab})=\overset{\text{amino acid evolution}}{\overset{︷}{p(A^{i}|H^{i},r_{a}^{i},r_{b}^{i},t_{ab})}}\times \overset{\text{dihedral angle evolution}}{\overset{︷}{p(X^{i}|H^{i},r_{a}^{i},r_{b}^{i},t_{ab})}}\times \overset{\text{secondary structure evolution}}{\overset{︷}{p(S^{i}|H^{i},r_{a}^{i},r_{b}^{i},t_{ab})}}.$$

The assumption of conditional independence provides computational tractability, allowing us to avoid costly marginalization when certain combinations of data are missing (e.g. amino acid sequences present, but secondary structures and dihedral angles missing).

### Stochastic Processes: Modeling Evolutionary Dependencies

Each site-class couples together three time-reversible stochastic processes that separately describe the evolution of the three pairs of observation types, as in equation (1). Each site-class is intended to capture both physical and evolutionary features pertaining to sequence and structure. Parameters that correspond to a particular site-class are termed *site-class specific*, whereas parameters that are shared across all site-classes are termed *global*. The use of site-class specific parameters, such as site-class specific amino acid frequencies and dihedral angle diffusion parameters, as described in the next section, is intended to model site-specific physical–chemical properties (Halpern and Bruno 1998; Koshi and Goldstein 1998; Lartillot and Philippe 2004).

#### Amino Acid Evolution

As is typical with models of sequence evolution, amino acid evolution, $p(A_{a}^{x(i)},A_{b}^{y(i)}|H^{i},r_{a}^{i},r_{b}^{i},t_{ab})$, is described by a Continuous-Time Markov Chain (CTMC). Each amino acid CTMC is parameterized in the following way: the exchangeability of amino acids is described by a 20 × 20 symmetric global exchangeability matrix *S* (190 free parameters; Whelan and Goldman 2001), a site-class specific set of 20 amino acid equilibrium frequencies $\Pi_{r}^{h}=\text{diag}\{\pi_{1},\pi_{2},\ldots ,\pi_{20}\}$ (19 free parameters per site-class) and a site-class specific scaling factor $\Lambda_{r}^{h}$ (one free parameter per site-class). Together these parameters define a site-class specific time-reversible amino acid rate matrix $Q_{r}^{h}=\Lambda_{r}^{h}S\Pi_{r}^{h}$. The stationary distribution of $Q_{r}^{h}$ is given by the amino acid equilibrium frequencies: $\Pi_{r}^{h}$.

#### Secondary Structure Evolution

Secondary structure evolution, $p(S_{a}^{x(i)},S_{b}^{y(i)}|H^{i},r_{a}^{i},r_{b}^{i},t_{ab})$, is also described by a CTMC. For the sake of simplicity we use only three discrete classes to describe secondary structure at each position: helix (H), sheet (S), and random coil (C).

The exchangeability of secondary structure classes at a position is described by a 3 × 3 symmetric global exchangeability matrix *V* and a site-class specific set of three secondary structure equilibrium frequencies $\Omega_{r}^{h}=\text{diag}\{\pi_{1},\pi_{2},\pi_{3}\}$. Together they define a site-class specific time-reversible secondary structure rate matrix $R_{r}^{h}=V\Omega_{r}^{h}$, with stationary distribution: $\Omega_{r}^{h}$.

#### Dihedral Angle Evolution

Central to our model is evolutionary dependence between dihedral angles, $p(X_{a}^{x(i)},X_{b}^{y(i)}|H^{i},r_{a}^{i},r_{b}^{i},t_{ab})$. Typically, the continuous-time evolution of the continuous-state random variables is modeled by a diffusive process such as the OU process, as in Challis and Schmidler (2012). However, an OU process is not appropriate for dihedral angles as they have a natural periodicity. For this reason, a bivariate diffusion that captures the periodic nature of dihedral angles, the *Wrapped Normal* (WN) *diffusion*, was specifically developed for this paper in García-Portugués et al. (2017).

**Fig. 2:**
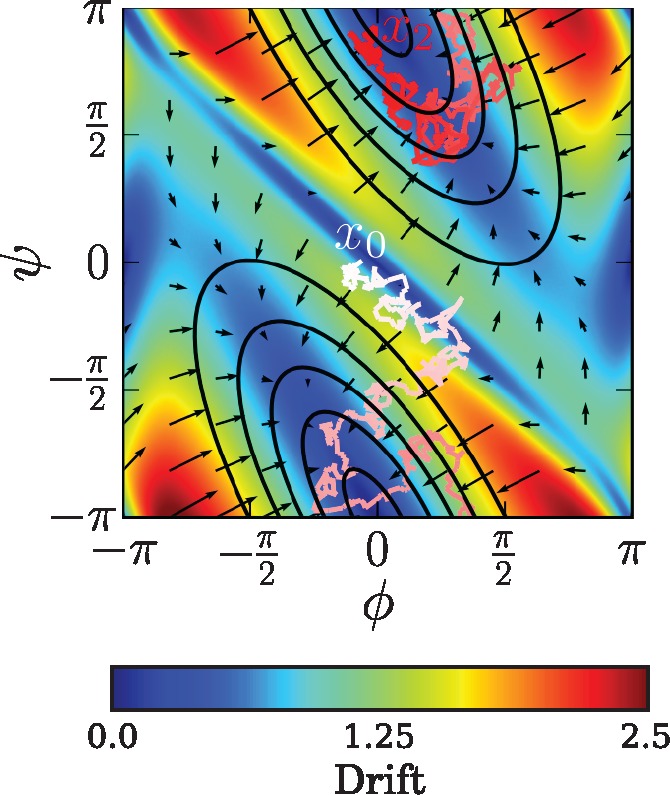
Drift vector field for the WN diffusion with A=(1,0.5;0.5,0.5), μ=(0,0) and $\Sigma ={\left(1.5\right)}^{2}I$. The color gradient represents the Euclidean norm of the drift. The contour lines represent the stationary distribution. An example trajectory starting at *x*_0_ = (0,0) and ending at *x*_2_, running in the time interval [0, 2] is depicted using a white to red color gradient indicating the progression of time. The periodic nature of the diffusion can be seen by the wrapping of both the stationary diffusion and the trajectory at the boundaries of the square plane. The fact that stationary distribution is not aligned with the horizontal and vertical axes illustrates the dependence (given by *α*_3_) between the ϕ and *ψ* dihedral angles.

Topologically, the WN diffusion (see fig. 2 for a pictorial example) can be thought of as the analogue of the OU process on the torus $T^{2}=[-\pi ,\pi )\times [-\pi ,\pi )$. The WN diffusion arises as the wrapping on $T^{2}$ of the following Euclidean diffusion:

(2) $$dX_{t}=\overset{\overset{\text{drift}}{\text{coefficient}}}{\overset{︷}{A\sum_{k\in {\mathbb{Z}}^{2}}(\mu -X_{t}-2k\pi )w_{k}(X_{t})}}dt\;+\overset{\overset{\text{diffusion}}{\text{coefficient}}}{\overset{︷}{\Sigma^{\frac{1}{2}}}}dW_{t},$$

where *W_t_* is the two-dimensional Wiener process, *A* is the drift matrix, $\mu \in T^{2}$ is the stationary mean, Σ is the infinitesimal covariance matrix and

(3) $$w_{k}(\theta )=\frac{\varphi_{\frac{1}{2}A^{-1}\Sigma }(\theta -\mu +2k\pi )}{\sum_{m\in {\mathbb{Z}}^{2}}\varphi_{\frac{1}{2}A^{-1}\Sigma }(\theta -\mu +2m\pi )},\theta \in T^{2},$$

is a probability density function (pdf) for $k\in {\mathbb{Z}}^{2}$. $\varphi_{\Sigma }$ stands for the pdf of a bivariate Gaussian N(0,Σ). The pdf (eq. 3) weights the linear drifts of equation (2) such that they become smooth and periodic.

It is shown in García-Portugués et al. (2017) that the stationary distribution of the WN diffusion is a WN(μ,Σ), which has pdf:

(4) $$p_{\text{WN}}(\theta |\mu ,\Sigma )=\sum_{k\in {\mathbb{Z}}^{2}}\varphi_{\Sigma }(\theta -\mu +2k\pi ).$$

Despite involving an infinite sum over ${\mathbb{Z}}^{2}$, taking just the first few terms of this sum provides a tractable and accurate approximation to the stationary density for most of the realistic parameter values.

Maximum Likelihood Estimation (MLE) for diffusions is based on the transition probability density (tpd), which only has a tractable analytical form for very few specific processes. A highly tractable and accurate approximation to the tpd is given for the WN diffusion. This approximation results from weighting the tpd of the OU process in the same fashion as the linear drifts are weighted in equation (2), yielding the following multimodal pseudo-tpd:

(5) $$\tilde{p}(\theta_{2}|\theta_{1},A,\mu ,\Sigma ,t)=\sum_{m\in {\mathbb{Z}}^{2}}p_{\text{WN}}(\theta_{2}|\mu_{t}^{m},\Gamma_{t})w_{m}(\theta_{1}),$$

with $\theta_{1},\theta_{2}\in T^{2},\;\mu_{t}^{m}=\mu +e^{-tA}(\theta_{1}-\mu +2\pi m)$ and $\Gamma_{t}=\int_{0}^{t}e^{-sA}\Sigma e^{-sA^{T}}ds$. The pseudo-tpd provides a good approximation to the true tpd in key circumstances: 1) t→0, since it collapses in the Dirac delta; 2) t→∞, since it converges to the stationary distribution; 3) high concentration, since the WN diffusion becomes an OU process. Furthermore, it is shown in García-Portugués et al. (2017) that the pseudo-tpd has a lower Kullback–Leibler divergence with respect to the true tpd than the Euler and Shoji-Ozaki pseudo-tpds, for most typical scenarios and discretization times in the diffusion trajectory.

A further desirable property of the pseudo-tpd is that it obeys the time-reversibility equation, which in terms of $\left(X_{a}^{x(i)},X_{b}^{y(i)}\right)$ is

$$\begin{array}{c} \tilde{p}(X_{b}^{y(i)}|X_{a}^{x(i)},A,\mu ,\Sigma ,t_{ab})p_{\text{WN}}(X_{a}^{x(i)}|\mu ,\frac{1}{2}A^{-1}\Sigma )\\ =\tilde{p}(X_{a}^{x(i)}|X_{b}^{y(i)};A,\mu ,\Sigma ,t_{ab})p_{\text{WN}}(X_{b}^{y(i)}|\mu ,\frac{1}{2}A^{-1}\Sigma ). \end{array}$$

Indeed, the WN diffusion is the *unique* time-reversible diffusion with constant diffusion coefficient and stationary pdf (eq. 4), in the same way the OU is with respect to a Gaussian. Time-reversibility is an assumption of the overall model and many other models of sequence evolution. A benefit of time-reversibility in a pairwise model such as ETDBN is that one of the proteins in a pair may be arbitrarily chosen as the ancestor, thus avoiding a computationally expensive marginalization of an unobserved ancestor.

The likelihood of a dihedral angle observation pair $\left(X_{a}^{x(i)},X_{b}^{y(i)}\right)$, assuming that $X_{a}^{x(i)}$ is drawn from the stationary distribution, is given by:

(6) $$\begin{array}{c} p(X_{a}^{x(i)},X_{b}^{y(i)}|H^{i},r_{a}^{i},r_{b}^{i},t_{ab})\\ =p(X_{a}^{x(i)},X_{b}^{y(i)}|A,\mu ,\Sigma ,t_{ab})\\ \approx \tilde{p}(X_{b}^{y(i)}|X_{a}^{x(i)},A,\mu ,\Sigma ,t_{ab})p_{\text{WN}}(X_{a}^{x(i)}|\mu ,\frac{1}{2}A^{-1}\Sigma ), \end{array}$$

*A* and Σ are constrained to yield a covariance matrix $A^{-1}\Sigma$. A parameterization that achieves this is $\Sigma =\text{diag}(\sigma_{1}^{2},\sigma_{2}^{2})$ and $A=(\alpha_{1},\frac{\sigma_{1}}{\sigma_{2}}\alpha_{3};\frac{\sigma_{2}}{\sigma_{1}}\alpha_{3},\alpha_{2}),\;\alpha_{1}\alpha_{2}>\alpha_{3}^{2}$. *α*_1_ and *α*_2_ are the drift components for the φ and *ψ* dihedral angles, respectively. Dependence (correlation) between the dihedral angles is captured by *α*_3_. A depiction of a WN diffusion with given drift and diffusion parameters is shown in figure 2.

### Site-Classes: Constant Evolution and Jump Events

We now turn to the meaning of the site-class pairs. Two modes of evolution are modeled: *constant evolution* and *jump events*. Constant evolution occurs when the site-class starting in protein *p_a_* at aligned site *i*, $r_{a}^{i}$, is the same as the site-class ending in protein *p_b_* at aligned site *i*, $r_{b}^{i}$, that is, $r_{a}^{i}=r_{b}^{i}$. Thus the distribution over observation pairs at a site is specified by a single site-class.

As already stated, a site-class specifies the parameters of the three conditionally independent stochastic processes describing amino acid, dihedral angle, and secondary structure evolution. A limitation of “constant evolution” is that the coupling between the three stochastic processes is somewhat weak. This in part stems from the time-reversibility of the stochastic processes—swapping the order of one of the three observation pairs at a homologous site, e.g. (Glycine, Proline) instead of (Proline, Glycine), does not alter the likelihood in equation (1). Alternatively restated from a generative perspective: a ‘directional coupling’ of an amino acid interchange does not inform the direction of change in dihedral angle or secondary structure. For example, replacing a glycine in an *α*-helix in one protein with a proline at the homologous position in a second protein would be expected to break the *α*-helix in the second protein and to strongly inform the plausible dihedral angle conformations in the second protein.

Ideally, we would consider a model in which the underlying site-classes were not fixed over the evolutionary trajectory separating the two proteins, as in the case of constant evolution as described above, but instead were able to ‘evolve’ in time. This would allow occasional switches in the underlying site-class at a particular homologous site, which would create a stronger dependency between amino acid, dihedral angle and secondary structure evolution, that furthermore captures the directional coupling we desire. Such an approach is considered computationally intractable due to the introduction of context-dependence when having to consider neighboring dependencies amongst evolutionary trajectories at adjacent sites.

In order to approximate this ‘ideal’ model in a computationally efficient manner we introduce the notion of a *jump event*. A jump event occurs when $r_{a}^{i}\neq r_{b}^{i}$. Whereas constant evolution is intended to capture angular drift (changes in dihedral angles localized to a region of the Ramachandran plot), a jump event is intended to create a directional coupling between amino acid and structure evolution, and is also expected to capture angular shift (large changes in dihedral angles, possibly between distant regions of the Ramachandran plot).

The hidden state at node *H^i^*, together with the evolutionary time *t_ab_* separating proteins *p_a_* and *p_b_*, specifies a joint distribution over a site-class pair:

(7) $$p(r_{a}^{i},r_{b}^{i}|H^{i},t_{ab})=p(r_{a}^{i}|H^{i},r_{b}^{i},t_{ab})p(r_{b}^{i}|H^{i}),$$

where

$$\begin{array}{c} p(r_{a}^{i}|H^{i},r_{b}^{i},t_{ab})\\ =\{\begin{array}{cc} e^{-\gamma_{H^{i}}t_{ab}}+\pi_{H^{i},r_{b}^{i}}(1-e^{-\gamma_{H^{i}}t_{ab}}), & \text{if }r_{a}^{i}=r_{b}^{i},\\ \pi_{H^{i},r_{b}^{i}}(1-e^{-\gamma_{H^{i}}t_{ab}}), & \text{if }r_{a}^{i}\neq r_{b}^{i}, \end{array} \end{array}$$

and $p(r_{a}^{i}|H^{i})=\pi_{H^{i},r_{a}^{i}}$ and $p(r_{b}^{i}|H^{i})=\pi_{H^{i},r_{b}^{i}}$. $\pi_{H^{i},r_{a}^{i}}$ and $\pi_{H^{i},r_{b}^{i}}$ are model parameters specifying the probability of starting in site-class $r_{a}^{i}$ or $r_{b}^{i}$, respectively, corresponding to the hidden state specified by node *H^i^*. $\gamma_{H^{i}}>0$ is a model parameter giving the jump rate corresponding to the hidden state given by node *H^i^*.

The site-class jump probabilities have been chosen so that time-reversibility holds, in other words:

$$p(r_{a}^{i}|H^{i},r_{b}^{i},t_{ab})p(r_{b}^{i}|H^{i})=p(r_{b}^{i}|H^{i},r_{a}^{i},t_{ab})p(r_{a}^{i}|H^{i}).$$

The hidden state at node *H^i^*, together with a site-class pair $\left(r_{a}^{i},r_{b}^{i}\right)$ and the evolutionary time *t_ab_*, specifies the joint likelihood over site observation pairs:

(8) $$\begin{array}{c} p(O_{a}^{x(i)},O_{b}^{y(i)}|H^{i},r_{a}^{i},r_{b}^{i},t_{ab})\\ =\{\begin{array}{cc} p(O_{a}^{x(i)},O_{b}^{y(i)}|H^{i},r_{c}^{i},t_{ab}), & \text{if }r_{a}^{i}=r_{b}^{i}=r_{c}^{i},\\ p(O_{a}^{x(i)}|H^{i},r_{a}^{i})p(O_{b}^{y(i)}|H^{i},r_{b}^{i}), & \text{if }r_{a}^{i}\neq r_{b}^{i}. \end{array} \end{array}$$

In the case of constant evolution, evolution at aligned *i* is described in terms of the same site-class $r_{c}^{i}$. Evolution is considered constant because each observation type is drawn from a single stochastic process specified by *H^i^* and *r_c_*. Note that the strength of the evolutionary dependency within an observation pair is a function of the evolutionary time *t_ab_*.

In the case of a jump event, the evolutionary processes are, after the evolutionary jump, restarted independently in the stationary distribution of the new site-class. Thus the site observations $O_{a}^{x(i)}$ and $O_{b}^{y(i)}$ are assumed to be drawn from the stationary distributions of two separate stochastic processes corresponding to site-classes $r_{a}^{i}$ and $r_{b}^{i}$, respectively. This implies that, conditional on a jump, the likelihood of the observations is no longer dependent on *t_ab_*. A jump event is an abstraction that captures the end-points of the evolutionary process, but ignores the potential evolutionary trajectory linking the two site observations. The advantage of abstracting the evolutionary trajectory is that there is no need to perform a computationally expensive marginalization over all possible trajectories, as might be necessary in a model where the hidden states evolve along a tree. The likelihood of an observation pair is now simply a sum over the four possible site-class pairs:

$$\begin{array}{c} p(O^{x(i)},O^{y(i)}|H^{i},t_{ab})\\ =\sum_{(r_{a}^{i},r_{b}^{i})\in R}p(O^{x(i)},O^{y(i)}|H^{i},r_{a}^{i},r_{b}^{i},t_{ab})p(r_{a}^{i},r_{b}^{i}|H^{i},t_{ab}), \end{array}$$

where R={(1,1),(1,2),(2,1),(2,2)} is the set of four site-class pairs, $p(O^{x(i)},O^{y(i)}|H^{i},r_{a}^{i},r_{b}^{i})$ is given by equation (8) and $p(r_{a}^{i},r_{b}^{i}|H^{i},t_{ab})$ is given by equation (7).

### Identification of Evolutionary Motifs Encoding Jump Events

In order to identify aligned sites having potential evolutionary motifs encoding jump events, a specific criterion was developed.

For a particular protein pair, inference was performed under the model conditioned on the amino acid sequence and dihedral angles for both proteins $\left(A_{a},A_{b},X_{a},X_{b}\right)$. Homologous sites corresponding to a single hidden state and with evidence of a jump event ($r_{a}^{i}\neq r_{b}^{i}$) at posterior probability > 0.90 were identified, that is, the *i*’s such that $p(H^{i},r_{a}^{i}\neq r_{b}^{i}|A_{a},A_{b},X_{a},X_{b})>0.90$.

In a second filtering step, amino acid sequences and a single set of dihedral angles corresponding to one of the proteins were used ($A_{a},A_{b},X_{a}$ or $A_{a},A_{b},X_{b}$) to infer the posterior probability, this time at a lower threshold: $p(H^{i},r_{a}^{i}\neq r_{b}^{i}|A_{a},A_{b},X_{a})>0.50$ or $p(H^{i},r_{a}^{i}\neq r_{b}^{i}|A_{a},A_{b},X_{b})>0.50$. This second criterion ensured that the evolutionary motif was identifiable under typical conditions where one has limited access to structural information (in this case a single protein structure in a pair). Only those aligned sites meeting both criteria were selected for downstream analysis.

### Statistical Alignment: Modeling Insertions and Deletions

Protein sequences can not only undergo amino transitions due to underlying nucleotide mutations in the coding sequence, but also indel events. To account this, a modified pairwise TKF92 alignment HMM based on Miklós et al. (2004) was implemented. The TKF92 alignment HMM was augmented with the ETDBN evolutionary hidden states in order to capture local sequence and structure evolutionary dependencies. Furthermore, it was modified such that neighboring dependencies amongst hidden states at adjacent alignment sites were modeled. For more details see ‘Statistical alignment’ in Supplementary Material online.

Whilst it is possible to fix the alignment in advance by pre-aligning the sequences using one of the many available alignment methods (Katoh et al. 2002; Edgar 2004) or using a curated alignment (such as from the HOMSTRAD database), doing so ignores alignment uncertainty.

### Training and Test Datasets

A training dataset of 1,200 protein pairs (2,400 proteins; 417,870 site observation pairs) and a test dataset of 38 protein pairs (76 proteins; 14,125 site observation pairs) were assembled from 1,032 protein families in the HOMSTRAD database. For further details see ‘Construction of test and training datasets' in the supplementary Material, Supplementary Material online.

### Model Training and Selection

Maximum Likelihood Estimation of the model parameters, $\hat{\Psi }$, was done using Stochastic Expectation Maximization (StEM, Gilks et al. 1995).

For further details of the E- and M-steps of the StEM algorithm and for details about model selection please refer to ‘Model training and selection’ in the supplementary Material, Supplementary Material online.

## Results and Discussion

### Selecting the Number of Hidden States

**Fig. 3:**
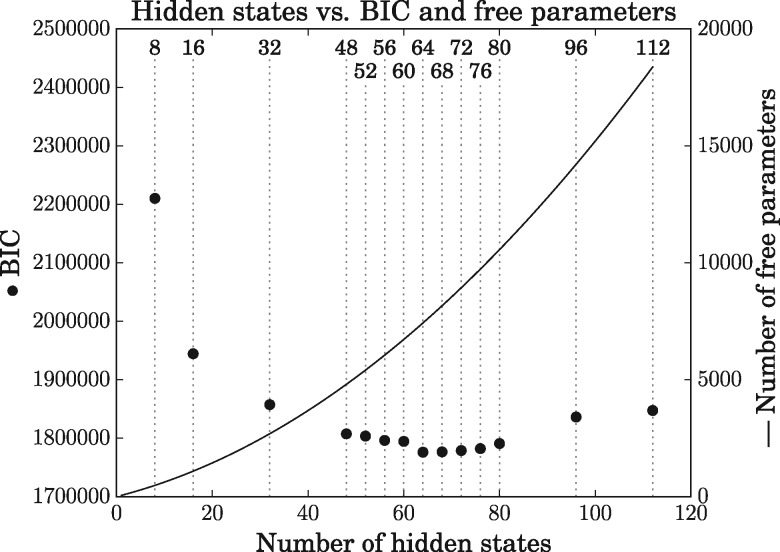
BIC scores (points) and number of free parameters (curve) as a function of the number of hidden states across 14 models (indicated above the dotted vertical lines) trained using the 1,200 protein pairs in the training dataset. A 64 hidden state model had the lowest BIC score. Each model represents the best of several attempts.

Fourteen models were trained (8, 16, 32, 48, 52, 56, 60, 64, 68, 72, 76, 80, 96, and 112 hidden state models). The 64 hidden state model was chosen as the best model, as it had the lowest Bayesian Information Criterion (BIC, fig. 3).

### Stationary Distributions over Dihedral Angles Capture the Empirical Distribution

**Fig. 4:**
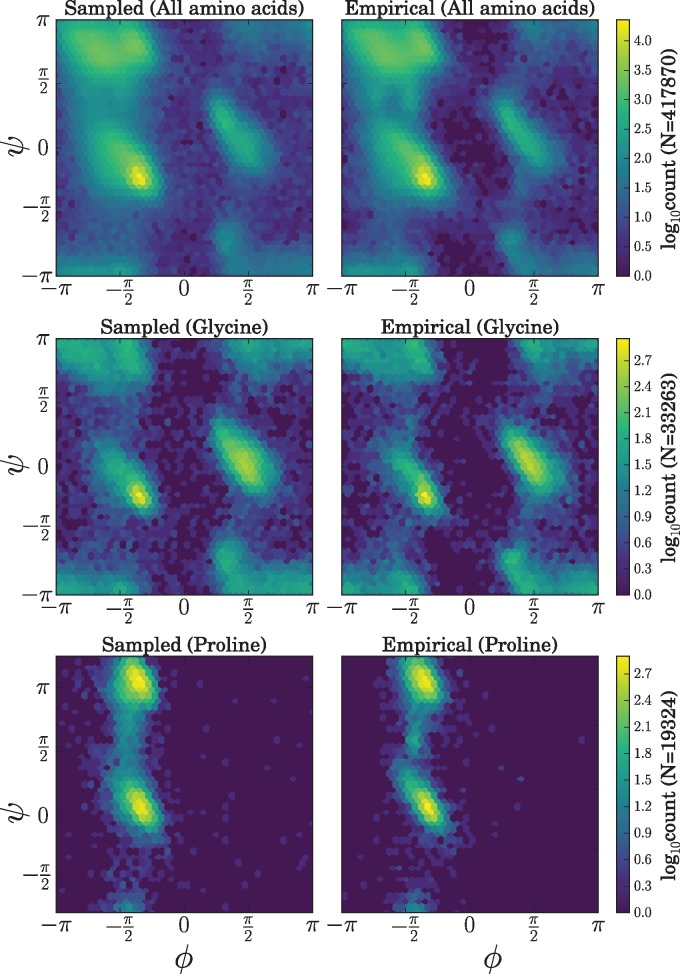
Ramachandran plots depicting sampled and empirical dihedral angle distributions. The top row depicts the distributions for all amino acids, the middle for glycine only and the bottom for proline only. The leftmost plots show dihedral angles sampled under the jump model, whereas the rightmost plots show the empirical distributions of dihedral angles in the training dataset.

Figure 4 illustrates the sampled and empirical dihedral angle distributions. There is a good correspondence between dihedral angles sampled under the model (fig. 4, left) and the empirical distribution of dihedral angles in our training dataset (fig. 4, right) for all three cases illustrated (all amino acids, glycine only, and proline only). The correspondence is not surprising given that ETDBN is effectively a mixture model with a large number of mixture components.

### Estimates of Evolutionary Time from Dihedral Angles Are Consistent with Estimates from Sequence

Whilst ETDBN has a general scope with respect to applications (including acting as proposal distribution or as a building block in a homology modeling application), we envision the primary application being inference of evolutionary parameters.

**Fig. 5:**
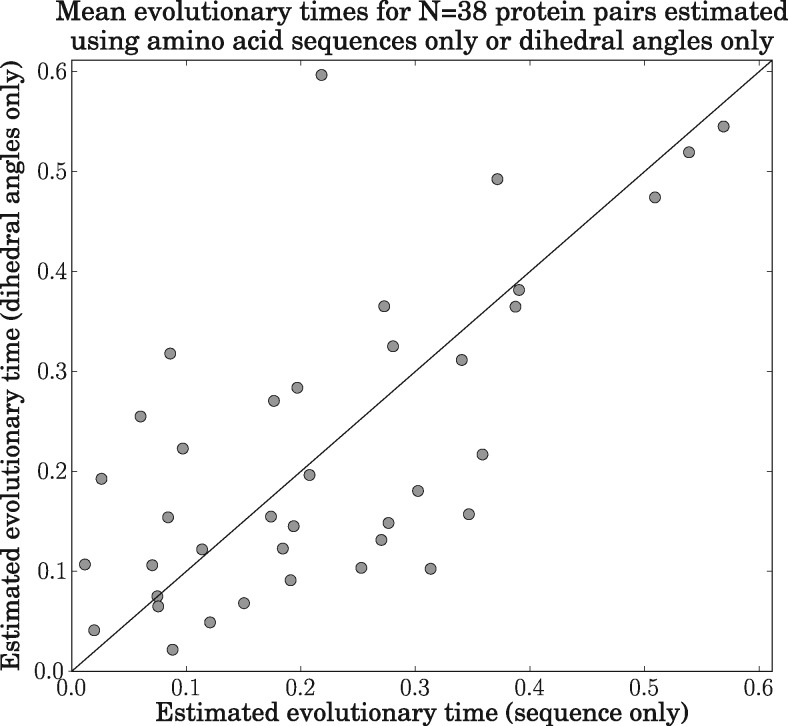
Scatterplot comparing evolutionary times estimated using pairs of homologous amino acid sequences only versus pairs of homologous sets of dihedral angles only for *N* = 38 proteins pairs in the test dataset. The *x*-coordinate of each point gives the estimated evolutionary time based only on the amino acid sequence, whereas the *y*-coordinate gives the estimated evolutionary time based only on the dihedral angles. The diagonal line represents *y *=* x*.

Figure 5 compares evolutionary times estimated using only pairs of homologous amino acid sequences versus only pairs of homologous dihedral angles. As desired, the two estimates of evolutionary time for each protein pair are similar, as can be seen by the proximity of the points to the identity line.

A paired *t*-test gave a *p*-value of 0.578, thus failing to reject the null hypothesis that there is no difference between branch lengths estimated using sequence only versus angles only. This indicates that there is sufficient evolutionary information in the dihedral angles to estimate the evolutionary times and that the model is consistent in its estimates, lacking a significant tendency to underestimate or overestimate the evolutionary times when either sequence or dihedral angles are used.

Interestingly, the variance in the sampled evolutionary times is higher when dihedral angles only are used, as compared with sequence only (see fig. S1, Supplementary Material online).

### The Relationship between Evolutionary Time and Angular Distance Is Adequately Modeled

**Fig. 6:**
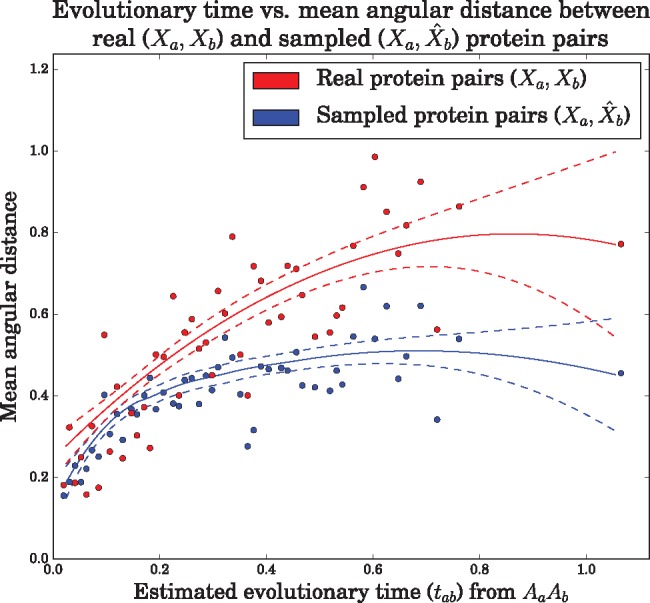
Evolutionary time versus angular distances between real and corresponding sampled proteins pairs in the training dataset at 50 representative evolutionary times. Mean angular distances (see ‘Calculation of angular distances’ in the supplementary Material, Supplementary Material online) between the real dihedral angles in a protein pair (red, *X_a_* and *X_b_*) and sampled dihedral angles in a sampled protein pair (blue, *X_a_* and ${\hat{X}}_{b}$) were compared with test how well the sampled dihedral angles reproduced the real angular distances. The dihedral angles (${\hat{X}}_{b}$) of each sampled protein pair were sampled by conditioning on both amino acid sequences and the homologous dihedral angles (*A_a_*, *A_b_*, *X_a_*), and the estimated evolutionary time (${\hat{t}}_{ab}$) for the real protein pair. The regression curves were obtained by a quadratic LOcally-weighted regrESSion (LOESS), with smoothing parameter chosen by leave-one-out cross-validation. The 95% confidence intervals for the mean assume error normality.

We investigated the relationship between evolutionary time and angular distance between real protein pairs and protein pairs where the dihedral angles of *p_b_* (*X_b_*) were treated as missing and hence sampled (fig. 6).

As expected, for both real and sampled pairs, angular distance tends to increase as a function evolutionary time. For larger evolutionary times a plateau begins to emerge, which is expected as the maximum possible theoretical angular distance is $\sqrt{8}\approx 2.828$.

When the evolutionary time is exactly zero (*t_ab_* = 0) under our model, the angular distance between sampled dihedral angles is exactly zero (not shown in fig. 6). However, this is not expected to be the case for real protein pairs when the two sequences are identical (due to the inherently flexible nature of proteins, different experimental conditions, experimental noise, etc.). It is therefore not surprising that the regression curve for the real protein pairs does not pass through zero.

For small evolutionary times (< 0.2) the curves for the real and sampled protein pairs show a good correspondence, however, for larger evolutionary times the model tends to underestimate angular distances. This may reflect the fact that the tpd of the WN diffusion specified is localized around its mean, even when the evolutionary time is large; therefore, dihedral angles distant from this mean are unlikely to be sampled. To a certain extent this is mitigated by the jump model, which occasionally allows for large changes in dihedral angle, but may still be somewhat limited in its flexibility, as jumps can only occur between two site-classes. The majority of protein pairs in our training dataset represent smaller evolutionary times (81.7% of evolutionary times are smaller than 0.4) and therefore protein pairs with larger evolutionary times and their associated jumps are underrepresented in our dataset, which may also explain the underestimation.

An additional possibility is that ETDBN does not attempt to model global dependencies. Echave and Fernández (2010) use a LFENM model (which does take into account global dependencies) and provide evidence showing that the majority of structural changes is due to collective global deformations rather than local deformations. A local model such as ETDBN, by definition, does not take into account global dependencies and therefore does not fully account for their contribution to structural divergence.

### Evaluation of the Model

The conditional independence structure in (eq. 1) enables computationally efficient sampling from the model under different combinations of observed or missing data. For example, ETDBN can be used to sample (i.e., predict) the dihedral angles of a protein from its corresponding amino acid sequence, a homologous amino acid sequence, a homologous set of dihedral angles, the corresponding secondary structure, a homologous secondary structure, or any combination of them.

Predictive accuracy was measured using 38 homologous protein pairs in the test dataset. For every protein pair (*p_a_*, *p_b_*), the dihedral angles of *p_b_* in each pair were treated as missing, and these missing dihedral angles were sampled under the model given a particular combination of observation types. The average angular distance between the sampled and known dihedral angles was used as the measure of predictive accuracy.

**Fig. 7:**
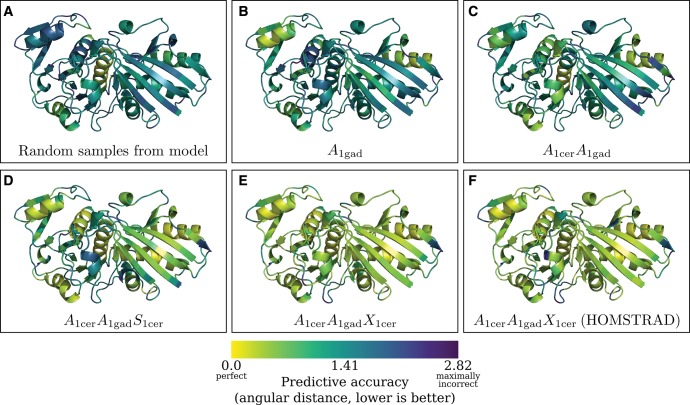
Cartoon structure representations of *E. coli* glyceraldehyde-3-phosphate dehydrogenase structure (PDB 1gad) are depicted in each panel, overlaid with predictive accuracy when using different combinations of observed data to predict missing dihedral angles in 1gad. *Thermus aquaticus* glyceraldehyde-3-phosphate dehydrogenase (PDB 1cer) was used as a homolog for the purposes of prediction. Predictive accuracy is indicated using a color gradient depicting the mean angular distance between the true dihedral angle ($X_{1\text{gad}}^{i}$) and the predicted (sampled) dihedral angles (${\hat{X}}_{1\text{gad}}^{i}$) at each amino acid position. The label at the bottom of each panel indicates the data combination used. In (*A*), no data was used for prediction. In (*B*), only the amino acid sequence corresponding to 1gad (*A*_1gad_) was used. In (*C*), the amino acid sequence of 1gad (*A*_1gad_) and the amino acid sequence of the homologous protein (*A*_1cer_) were used. In (*D*), both amino acids sequences (*A*_1cer_ and *A*_1gad_) and the secondary structure of the homologous protein (*S*_1cer_) were used. In (*E*), both the amino acid sequences (*A*_1cer_ and *A*_1gad_) and the dihedral angles of the homologous protein (*X*_1cer_) were used. Finally, in panel (*F*) the same combination of observations was used as in (*E*), but the alignment was treated as known a priori.

**Fig. 8:**
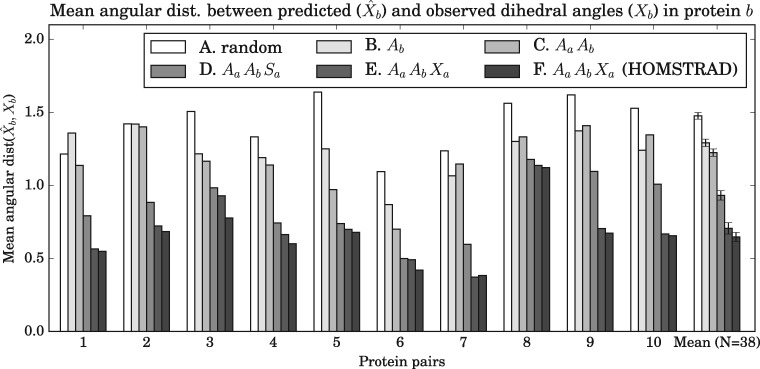
Benchmarks of predictive accuracy (measured using angular distance, lower is better) on a random subset of ten protein pairs in the test dataset, giving a representative view of predictive accuracy under six different combinations of observations. The dihedral angles *X_b_* of *p_b_* were treated as missing and were sampled under the model, whereas *p_a_* was a homologous protein used for the purposes of prediction. See the legend of figure 7 for a description of each combination (*A*–*F*). The final set of bars, denoted ‘Mean (*N* = 38)’, are the mean values for the entire test dataset of *N* = 38 protein pairs. The error bars are the standard errors.

Figure 7 gives an example of predictive accuracy under different combinations of observations types overlaid on a cartoon structure of the protein structure being predicted, whereas figure 8 provides a representative view of predictive accuracy across 10 different protein pairs in the test dataset for different combinations of observations types. We highlight some of the key patterns identified in figures 7 and 8 as follows.

Combination 1 refers to random sampling from the model, implying no data observations were conditioned on besides the respective lengths of proteins *p_a_* and *p_b_*. The average angular distance between the true and predicted dihedral angles was 1.6. Random sampling acts as a baseline for predictive accuracy. It is apparent from figure 7 that the model has a propensity to predict right-handed *α*-helices, which is the most populated region in the Ramachandran plot.

Under combination 2, only the amino acid sequence corresponding to *p_b_* is observed. As expected in figures 7 and 8 there is an increase in predictive accuracy with the addition of the amino acid sequence relative to combination 1.

Under combination 3, we add in the amino acid sequence of a homologous protein (*p_a_*). In all ten cases there is an improvement in predictive accuracy. The improvement in predictive accuracy is reasonable, as knowledge of the sequence evolutionary trajectory is expected to encode information about structure evolution and hence will inform the dihedral angle conformational possibilities.

Under combination 4, in addition to the two amino acid sequences we treat the homologous secondary structure as observed. This results in a substantial improvement in predictive accuracy as one would expect. Knowledge of the amino acid sequence and a homologous secondary structure strongly informs regions of the Ramachandran plot that are likely to be occupied.

Under combination 5 (which we consider the *canonical* combination—the standard homology modeling scenario), we treat both amino acid sequences as observed, as well as the dihedral angles of the homologous protein (*p_a_*)—in all cases the predictive accuracy improves over combination 4. This is anticipated as the homologous dihedral angles are expected to be the best proxy for missing dihedral angles and are therefore expected to be more informative than secondary structure alone. Note that the availability of a homologous amino acid sequence pair here and in combination 4 is consequential as it informs the evolutionary time *t_ab_* parameter, which will typically constrain the distribution over dihedral angles and reduce the associated uncertainty.

Finally, in combination 6, the same data observations as in combination 5 are used, except the alignment is treated as given a priori (by the HOMSTRAD alignment) rather than as unobserved. The HOMSTRAD alignment is based on a structural and sequence alignment of *p_a_* and *p_b_* and therefore is expected to encode a higher degree of homology and structural information than combination 5 (where the alignment is treated as unobserved and therefore a marginalization over alignments is performed). On average, there is a slight improvement in predictive accuracy when fixing the alignment, albeit the magnitude of improvement is not substantial. This demonstrates the accuracy of the alignment HMM.

The alignment HMM accounts for alignment uncertainty in a principled manner, which is particularly useful when an appropriate alignment is unavailable. However, it should be noted that inference scales $O(|p_{a}||p_{b}|h^{2})$ when treating the alignment as unobserved. Inference scales $O(mh^{2})$ when the alignment is fixed a priori, where *m* is the length of alignment *M_ab_* and is typically much smaller than $\left|p_{a}||p_{b}\right|$.

It should be emphasized that we do not expect ETDBN to compete with structure prediction packages such as Rosetta (Rohl et al. 2004) or homology modeling software such as Arnold et al. (2006) in terms of predictive accuracy. Our current model is a local model of structure evolution—it is not even expected capture fundamental constraints such as the radius of gyration of a protein or other global features typical of proteins.

### Evolutionary Hidden States Reveal a Common Evolutionary Motif

One benefit of ETDBN is that the 64 evolutionary hidden states learned during the training phase are interpretable. We give an example of a hidden state encoding a jump event that was subsequently found to represent an *evolutionary motif* present in a large number of protein pairs in our test and training datasets.

**Fig. 9:**
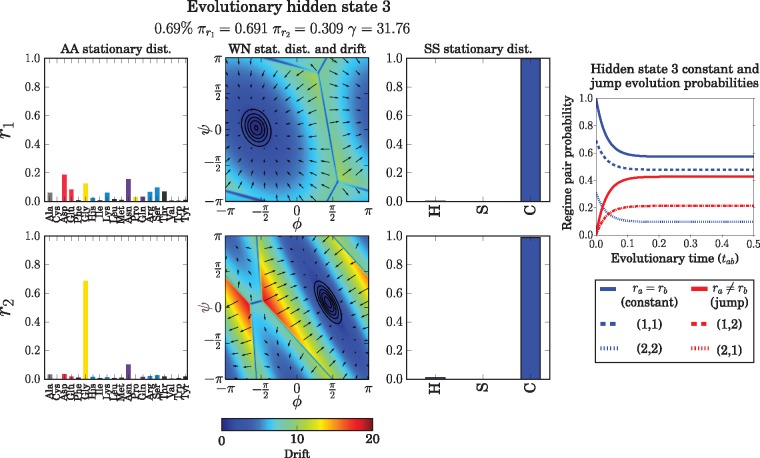
Depiction of evolutionary hidden state 3. This hidden state was sampled at 0.69% of sites (the average was 1.56%). The equilibrium frequencies of *r*_1_ and *r*_2_ were $\pi_{1}=0.691$ and $\pi_{2}=0.309$, respectively. The jump rate was *γ* = 31.76. The corresponding site-class pair probabilities are depicted to the right as a function of evolutionary time. Note that the dashed red lines depicting the probabilities for (1, 2) and (2, 1) superimpose exactly, because the probabilities are equal—this holds for the jump probabilities of all hidden states as it is required for time-reversibility. In the main figure, the two rows depict the parameters encoded by the two site-classes, respectively. Columns 1 and 3 depict the parameters governing the amino acid and secondary structure stochastic processes, respectively. The secondary structure classes correspond to H = helix, S = sheet, and C = coil. Column 2 depicts the WN diffusions. The stationary distributions of the WN diffusions are shown using black contour lines, the direction of the drifts are indicated by the arrows and the magnitude of the drifts at each position indicated using the color gradient.

Evolutionary hidden state 3 (fig. 9) was selected from the 64 hidden states as an example of a hidden state encoding a jump event and capturing angular shift (a large change in dihedral angle). A notable feature of this hidden state is that the change in dihedral angles between site-classes *r*_1_ and *r*_2_ is associated with specific amino acid changes. In site-class *r*_1_ the amino acid frequencies are relatively spread out amongst a number of amino acids, whereas in site-class *r*_2_ the frequencies are particularly concentrated in favor of glycine (Gly) and asparagine (Asp), with glycine being significantly more probable in site-class *r*_2_ than *r*_1_. This suggests that, conditioned on hidden state 3, an exchange between a glycine to another amino acid is likely indicative of a jump and hence a corresponding change in dihedral angle. This is consistent with what we find in a subsequent analysis of evolutionary motifs. This particular jump occurs in coil regions.

Having selected hidden state 3, positions in 238 protein pairs were analyzed for evidence of the corresponding evolutionary motif. 38 protein pairs in the test dataset and a further 200 from the training dataset were analyzed using the criteria described in the Methods section. Using the first criterion, 84 protein sites in 59 protein pairs corresponding to $H^{i}=3$ (evolutionary hidden state 3) were identified. Of the 84 protein sites, 34 protein sites met the second criterion.

**Fig. 10:**
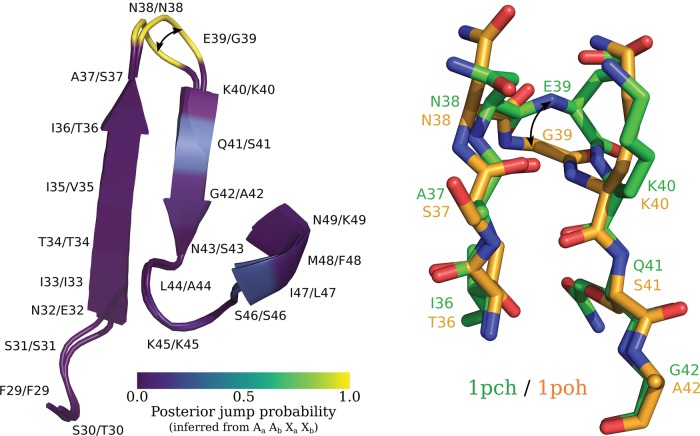
Depiction of two histidine-containing phosphocarriers, PDB 1pch and 1poh, superimposed. On the left is a cartoon representation of the two proteins corresponding to regions F29-K49 and F29-A42, respectively, with posterior jump probabilities at each position overlaid. On the right is a ball-and-stick representation giving atomic detail for a smaller region (I36-G42 and T36-A42, respectively). The exchange between a glutamate (E39 in 1poh) and a glycine (G39 in 1pch) is associated with a large change in dihedral angle as indicated by the curved arrows.

We give an example of a homologous protein pair illustrating the identified evolutionary motif. Two histidine-containing phosphocarriers, 1pch (*Mycoplasma capricolum*) and 1poh (*Escherichia coli*), were identified as having the evolutionary motif (fig. 10) at homologous site E39/G39.

Most positions in the homologous pair have low posterior jump probabilities (≈ 0.0), with the exception of positions N38/N38 and E39/G39, which both have high posterior jump probabilities (≈ 1.0). The exchange between a glutamate (at position 39 in 1poh) and a glycine (at position 39 in 1pch) appears to be responsible for the shift in dihedral angle. This exchange corresponds to a significant jump in dihedral angle: $\langle \varphi_{1\text{poh},E39},\psi_{1\text{pch},E39}\rangle =\langle -1.63,-0.06\rangle \to \langle \varphi_{1\text{poh},G39},\psi_{1\text{pch},G39}\rangle =\langle 1.40,0.22\rangle$. The angular distance between the two dihedral angles is 2.01. This is consistent with the amino acid frequency parameters specified by the two site-classes for hidden state 3 (fig. 9).

Site-class *r*_1_ indicates that a number of amino acids (alanine, aspartic acid, glycine, histidine, lysine, asparagine, proline, glutamine, arginine, serine, and theorine) other than glutamate plausibly coincide with the particular dihedral angle conformation specified by site-class *r*_1_. The involvement of glycine in a jump is not surprising as it is a small and flexible amino acid, whereas the role of asparagine is less clear. In our analysis of 238 protein pairs we found that of the seven positions meeting the criteria for hidden state 3 and involving an exchange with asparagine (Asn), four were an exchange between an asparagine and a glycine, whereas the remaining three were between asparagine and one of lysine, histidine, or serine.

### Using Dihedral Angles for Alignment

A valuable feature of our model is its ability to account for alignment uncertainty by summing over possible pairwise alignments using the TKF92 model as a prior distribution over indel histories, whilst simultaneously taking into account neighboring dependencies amongst aligned sites. Doing so results in a sample of alignments rather than a single alignment. Nevertheless, a single Maximum A Posteriori (MAP) pairwise alignment may be obtained from the alignment samples and used for downstream analysis.

ETDBN and several other alignment methods (namely StatAlign, BAli-Phy, MUSCLE, and MAFFT) were used to infer pairwise alignments from simulated and real data under various combinations of data observations, for example: an amino acid sequence pair (*A_a_*, *A_b_*), a secondary structure sequence pair (*S_a_*, *S_b_*), a dihedral angle sequence pair (*X_a_*, *X_b_*) and combinations thereof.

**Fig. 11:**
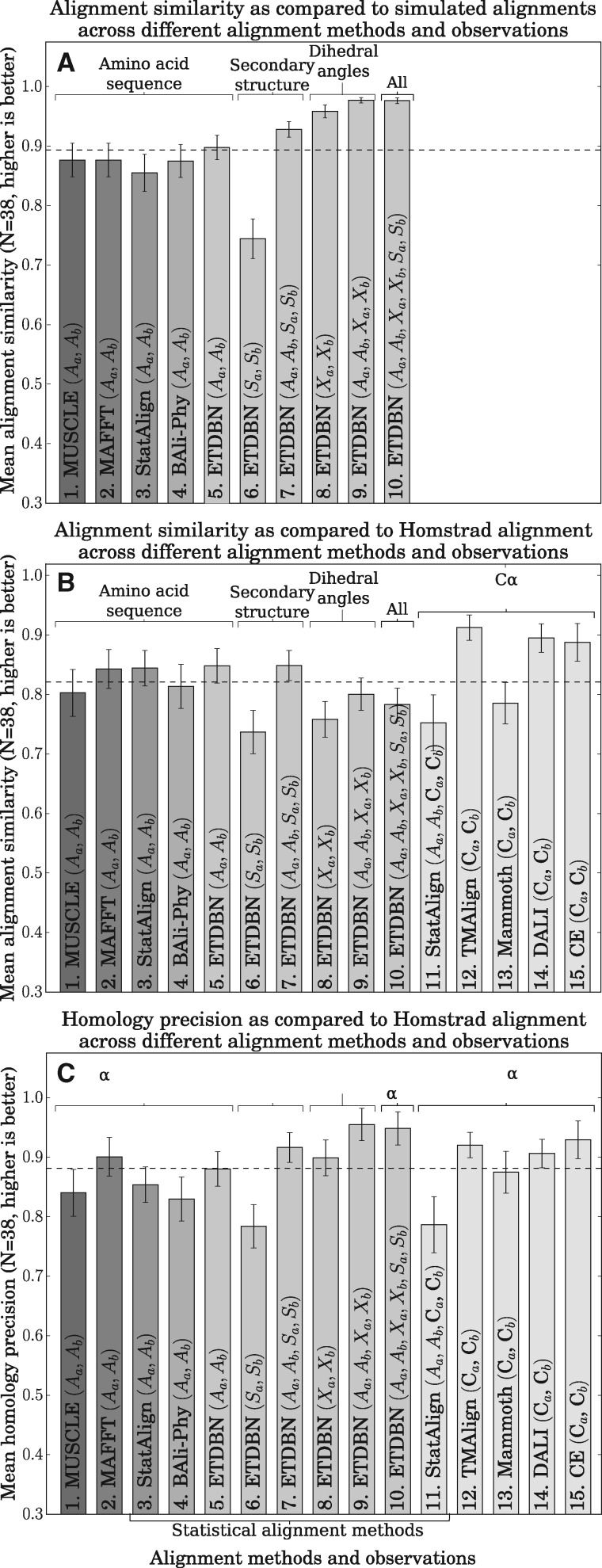
Alignment benchmarks. (*A*): averages of alignment similarity across different methods and combinations of data observations, where the observations were simulated from the ETDBN model conditioned on a single known alignment. (*B*): averages of alignment similarity, where the 38 HOMSTRAD alignments in the test dataset were taken as the true alignments. (*C*): averages of precision in predicting homologous site pairs in 38 sequence pairs from the test dataset, where homologous site pairs in the HOMSTRAD alignments were taken to be the true homologous site pairs.

In the first set of benchmarks (fig. 11*A*), pairs of proteins were simulated from the ETDBN model conditioned on 38 different pairwise alignments and corresponding evolutionary times. This resulted in a set of 38 simulated pairwise alignments together with corresponding observations, implying that the true underlying alignments were known for each of the simulated protein pairs. ETDBN and a number other alignment methods were used to infer pairwise alignments for each. The alignment similarity metric (Schwartz et al. 2005) was used to measure the similarity between the inferred alignments and the true alignments, where higher similarity indicates better predictions. It was found that, when using the simulated amino acid sequences alone, ETDBN (11A.5) outperformed all four other methods tested (11A.1 MUSCLE, 11A.2 MAFFT, 11A.3 StatAlign and 11A.4 BAli-Phy). However, the greater performance of ETDBN compared with other methods cannot be considered a fair comparison, as the data were simulated under the ETDBN model.

More revealing in figure 11*A* was the alignment similarity under ETDBN when using different combinations of simulated data observations. It was found that secondary structure alone (11A.6) performed the worst, which is unsurprising given that only three states were available to align the proteins. The second worst in terms of alignment similarity was amino acid sequences alone (11A.5), followed by amino acid sequences and secondary structures (11A.7). Interestingly, using dihedral angles only (11A.8) outperformed both 11A.5 (sequences only) and 11A.6 (secondary structures only). Finally, using amino acid sequence together with dihedral angles (11A.9) or all three data types combined (11A.10) outperformed all other combinations. This illustrates that, at least under simulation conditions, increasing the number of data observations results in better alignment accuracy.

Following that, the various alignment methods were benchmarked against 38 pairwise alignments consisting of real sequence and structure observations in the test dataset. These pairwise alignments were obtained from the HOMSTRAD alignments. The sequence identity of these pairwise alignments ranged from 10% to 93%, with an average sequence identity of 39%. In addition to methods 1–10 in figure 11*A*, five structural alignment methods were also used: 11. StatAlign (Herman et al. 2014), 12. TMAlign (Zhang and Skolnick 2005), 13. Mammoth (Ortiz et al. 2002), 14. Dali (Holm and Rosenström 2010), and 15. CE (Shindyalov and Bourne 1998). These methods were not used in 11A due to the lack of an appropriate model for simulating the evolution of three-dimensional protein structures.

When benchmarking the MAP estimated alignments against the HOMSTRAD alignments (fig. 11*B*), using real sequences alone for inference (*A_a_*, *A_b_*), ETDBN (11B.5) had a similar degree of accuracy when compared with several other sequence-based methods (11B.1 StatAlign, 11B.2 BaliPhy, 11B.3 MUSCLE, and 11B.4 MAFFT). This demonstrates that ETDBN has performance comparable to that of other commonly-used sequence alignment methods.

Using (*S_a_*, *S_b_*) alone, ETDBN (11B.6) had substantially lower alignment similarity compared with sequence only, which was expected given that a similar result was obtained for the simulated data (11A.6). However, when including the real sequences (11B.7) the predictive accuracy was once again comparable to sequence only inferences (11B.1–11B.5).

When using (*X_a_*, *X_b_*) alone (11B.8), the alignment similarity was found to be somewhat worse than the sequence only cases. Furthermore, when introducing the sequences (11.9) and secondary structures (11B.10) in addition to the dihedral angles, the similarity remained worse than the sequence only methods (11B.1–11B.5), despite the additional information. These results are in contrast to the results we obtained for simulated data (11A.8–11A.10).

The non-statistical structural alignment methods (11B.12–11B.15) faired the best, likely because they use a criteria similar to that used to align the HOMSTRAD alignments. When interpreting these results it is important to note the HOMSTRAD alignments should not be considered the true underlying alignments and may even be strongly biased. For example, they may favor the closest structural superimposition of structures or the most parsimonious alignments, with the fewest number of indels. In the evolutionary modeling context our goal is to distinguish between homologous sites (sites that have evolved via mutation alone) and indels. In practice, it is extremely difficult to obtain the true underlying alignment (sets of homologies and indels), because it would require an experiment where every indel event since the common ancestor is observed, a seeming impossible task outside of simulation or laboratory conditions.

After further investigation, the trend of lower alignment similarity seen in figure 11B.6–11B.10 when using ETDBN with structural observations compared with sequence only or non-statistical structural alignment methods was found to reverse (11C.6–11C.10) upon calculating the precision of predicting homologous sites (the fraction of sites which were predicted as homologous and were correctly predicted as such). Therefore when only dihedral angle observations are used, ETDBN underpredicts the number of homologous sites, however, when a homologous site is predicted, it is correctly predicted more often than when using only amino acid sequences. In particular, ETDBN predicted fewer homologous sites with coiled secondary structure compared with homologous sites with helical or sheet secondary structure. This pattern of results may be in part due to the WN diffusion used to model evolution of dihedral angles. The WN diffusion is suitable for modeling angular drift (small changes in angles localized around a region of the Ramachandran plot) but does not sufficiently capture angular shift (large changes in angles between regions of the Ramachandran plot, which are more likely in coiled regions) due to stationarity. As noted before, the jump model is an abstraction intended to capture the end-points of evolution by allowing a jump between two regions of the Ramachandran plot, abstracting a potential intermediate evolutionary trajectory for the sake of computational tractability. Note that the jump model accurately captures the common cases where a single mutation induces a large conformational shift.

## Concluding Remarks

The main achievement of this work is a computationally tractable, generative and interpretable probabilistic model of protein sequence and structure evolution on a local scale.

Previous stochastic models of protein sequence and structure evolution emphasized estimation of evolutionary parameters (Challis and Schmidler 2012; Herman et al. 2014). ETDBN is somewhat of a departure from these previous models, but is likewise capable of estimating evolutionary parameters. We show that estimates of evolutionary times inferred under ETDBN are consistent regardless of whether amino acid sequence or dihedral angle observations are used. In addition, the relationship between evolutionary time and angular distance in real proteins is adequately recapitulated in protein pairs sampled under the model, albeit the angular distance is underestimated for larger evolutionary times, which might be explained by the limited flexibility of the jump model and the lack of taking into account global dependencies.

Like previous models, ETDBN is capable of dealing with alignment uncertainty by marginalizing over indel histories; it predicts pairwise MAP consensus alignments with accuracy similar to that of score-based and statistical alignment methods.

The generative nature of ETDBN allows us to demonstrate that the underlying empirical distributions over dihedral angles (depicted using Ramachandran plots) are captured and that the model is capable of predicting missing observations, such as dihedral angles, from a variety of different data types. For example, an amino acid sequence, a homologous amino acid sequence, a homologous secondary structure, a homologous set of dihedral angles, or any combination thereof.

Based on its local nature, ETDBN does not constitute a homology modeling method in itself. Rather, it can be used as a building block, much like fragment libraries model local structure in protein structure prediction methods. ETDBN places the homology modeling problem on a statistical footing, enabling a number of approaches to later be used, such as multi-level modeling, that is, combining fine-grained distributions (for example, distributions over dihedral angles, such as ETDBN) and coarse-grained distributions (for example, distributions describing the global properties of proteins, such as compactness). In particular, a method referred to as ‘the Reference Ratio method’ can be used to combine fine-grained and coarse-grained distributions in a statistically principled manner (see Frellsen et al. 2012; Hamelryck et al. 2010). However, we have shown that the current model can already be used for the inference of evolutionary parameters in its present form.

In addition to multi-level modeling, probabilistic models such as ETDBN allow one to account for and to make statements about uncertainty (e.g. with respect to evolutionary time, alignment, etc.) in a rigorous manner. In principle, ETDBN, like TorusDBN (Boomsma et al. 2008), could be used as a proposal distribution. In other words, ETDBN could be used to sample protein structures (possibly conditioned on various data observations) in a computationally efficient manner, such that the resulting samples are expected to be located in regions of high probability density with respect to the true underlying distribution.

A final key feature of our evolutionary model is its interpretable nature. This interpretability enables the identification of potential evolutionary motifs—common patterns of sequence–structure evolution. We identify one such evolutionary motif in 34 different homologous protein pairs. A major direction for future research is the further identification of such evolutionary motifs. Understanding these evolutionary motifs, may 1) improve homology modeling predictions; 2) provide more accurate estimates of evolutionary parameters; and 3) produce better models of protein evolution that more realistically capture evolutionary trajectories through sequence and structure space, which may help identify functionally relevant positions that are potential drug targets.

## Future Challenges

### Pairwise to Phylogeny

For reasons of computational tractability the implemented model is pairwise, but it is theoretically possible to generalize it to a phylogeny, such as in Herman et al. (2014). In practice, for three or more sequences on a phylogeny it is necessary to marginalize out the unobserved ancestral protein states in order to compute likelihoods. Felsenstein’s algorithm can be used to marginalize over discrete ancestral states, such as amino acids in a computationally efficient manner. However, we do not know whether a similar efficient algorithm exists for marginalizing the continuous ancestral dihedral angle states under the WN diffusion, thereby necessitating a more expensive MCMC algorithm. A possibly greater computational hindrance to considering a phylogeny is the alignment problem, which scales $O(l_{1}\times l_{2}\times \ldots \times l_{N})$, where *l_i_* is the length of sequence *i* and *N* is the number of sequences, although MCMC approaches are possible (Herman et al. 2014).

### Context-Dependence

Although we believe our model provides a substantial improvement over current stochastic models of sequence and structural evolution, there is still scope for improvement. The WN diffusions used to model dihedral angle evolution adequately capture angular drift (small local changes in dihedral angle), but are less capable of capturing angular shift (large changes in dihedral angle). This is to a considerable extent mitigated by the introduction of jump events, as discussed before. A more realistic model would model the entire evolutionary trajectory, allowing an arbitrary number of switches between site-classes together with neighboring dependencies amongst adjacent sites along the evolutionary trajectory. Similar context-dependent models are typically computationally expensive and require sophisticated inference procedures (Robinson et al. 2003; Yu and Thorne 2006).

## Software Availability

Julia code (tested on both Windows and Linux platforms) is available at: http://www.computingforbiology.org/software/etdbn.

## Supplementary Material

Supplementary material are available at *Molecular Biology and Evolution* online.

## Supplementary Material

Supplementary Data
