# Supplementary material for: A Generative Angular Model of Protein Structure Evolution
Source: Mol Biol Evol. 2017 Apr 27;34(8):2085–100. doi: 10.1093/molbev/msx137 (PMC5850488; doi:10.1093/molbev/msx137)
Supplement: Supplementary Data [file msx137_Supp.zip › hiddenstates64.pdf]

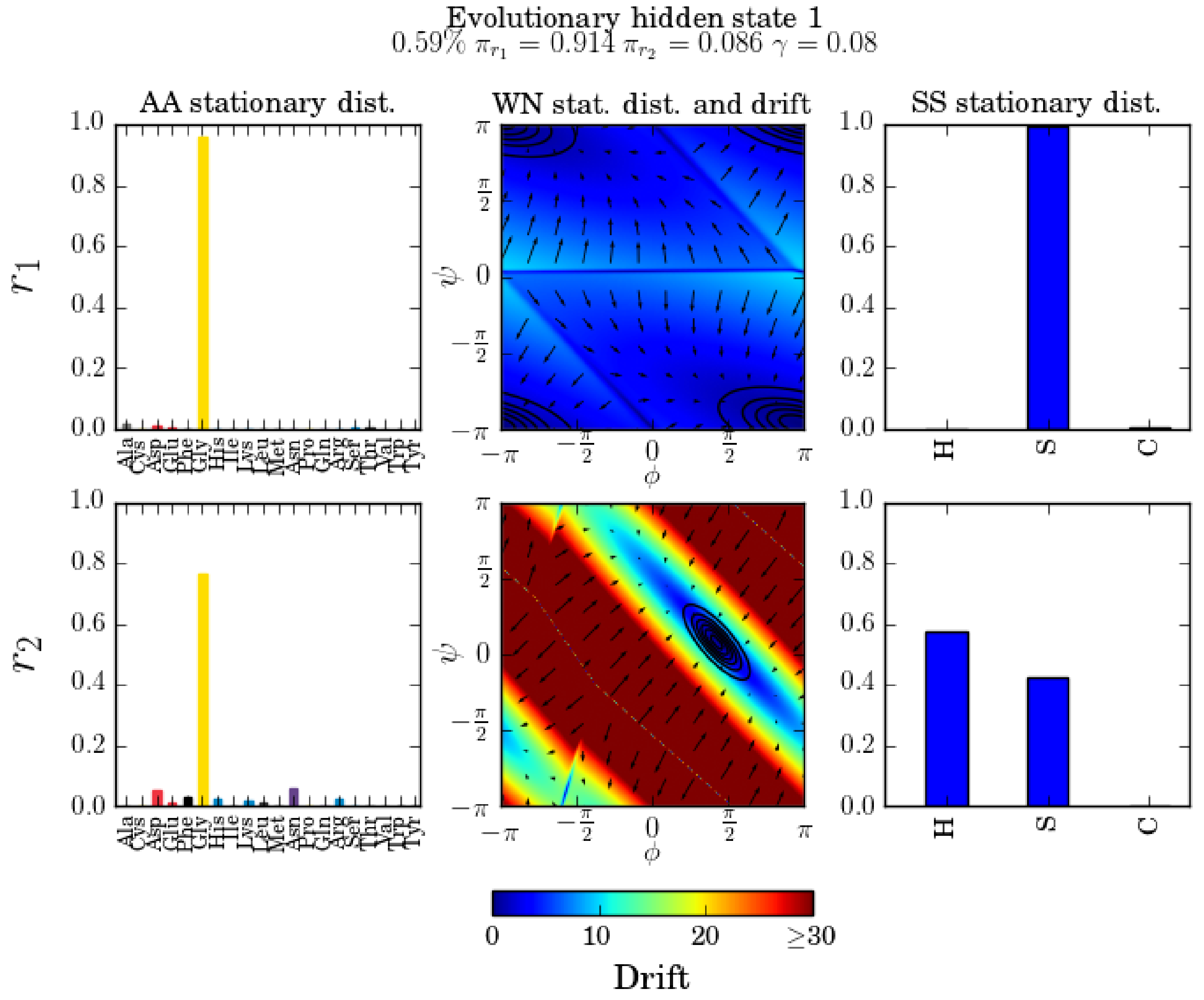

Evolutionary hidden state 1
0.59% $\pi_{r_1} = 0.914$ $\pi_{r_2} = 0.086$ $\gamma = 0.08$
AA stationary dist.
WN stat. dist. and drift
SS stationary dist.
r_1
r_2
1.0
0.8
0.6
0.4
0.2
0.0
Ala Cys Asp Glu Phe Gly His Ile Lys Leu Met Asn Pro Gln Arg Ser Thr Val Trp Tyr
ψ
ϕ
$-\pi$ $-\frac{\pi}{2}$ 0 $\frac{\pi}{2}$ π
H S C
0 10 20 ≥30
Drift

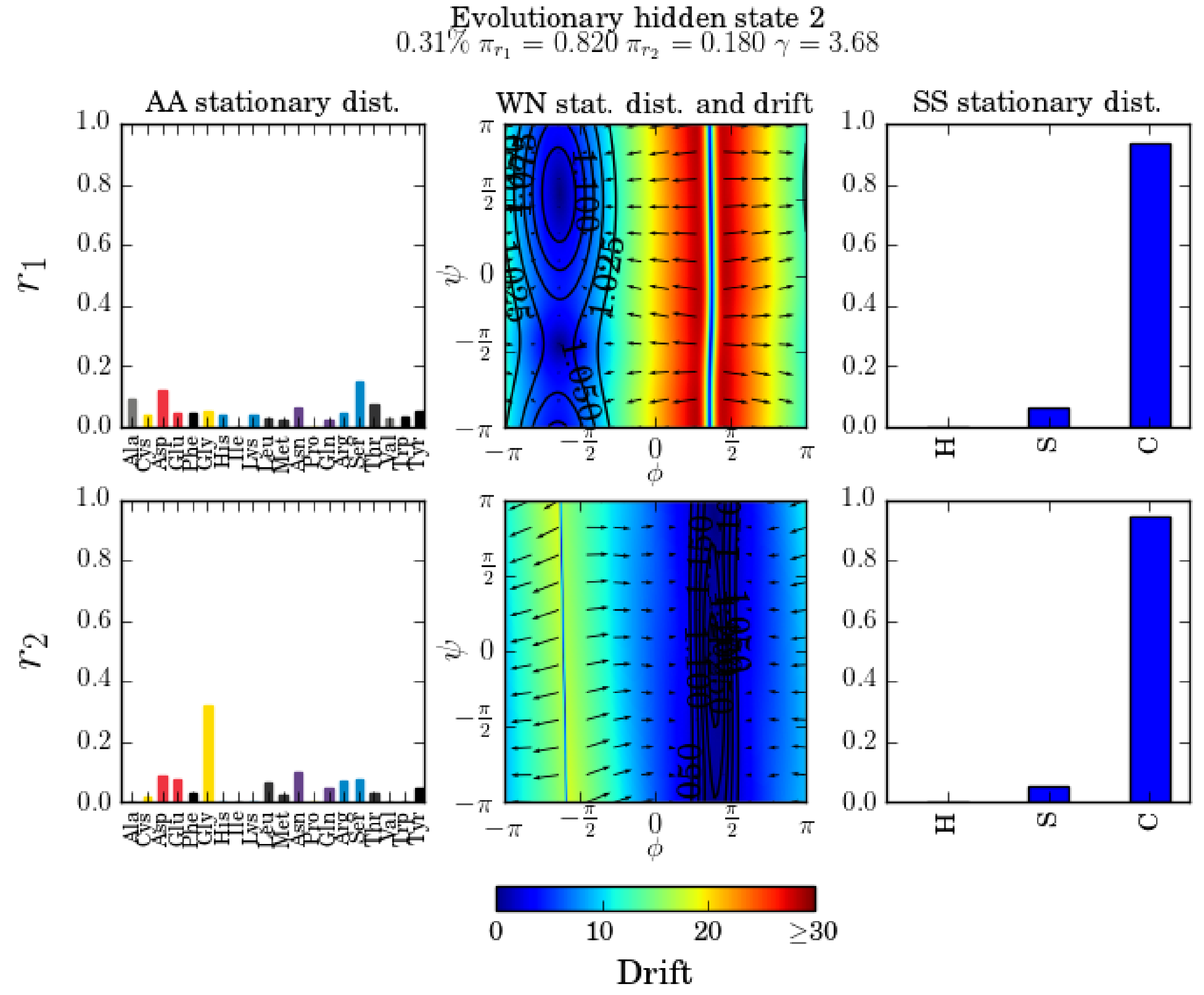
Evolutionary hidden state 2
0.31% $\pi_{r_1} = 0.820$ $\pi_{r_2} = 0.180$ $\gamma = 3.68$
AA stationary dist.
WN stat. dist. and drift
SS stationary dist.
r_1
r_2
Ala Cys Asp Glu Phe Gly His Ile Lys Leu Met Asn Pro Gln Arg Ser Thr Val Trp Tyr
H S C
ϕ
ψ
Drift
0 10 20 ≥30

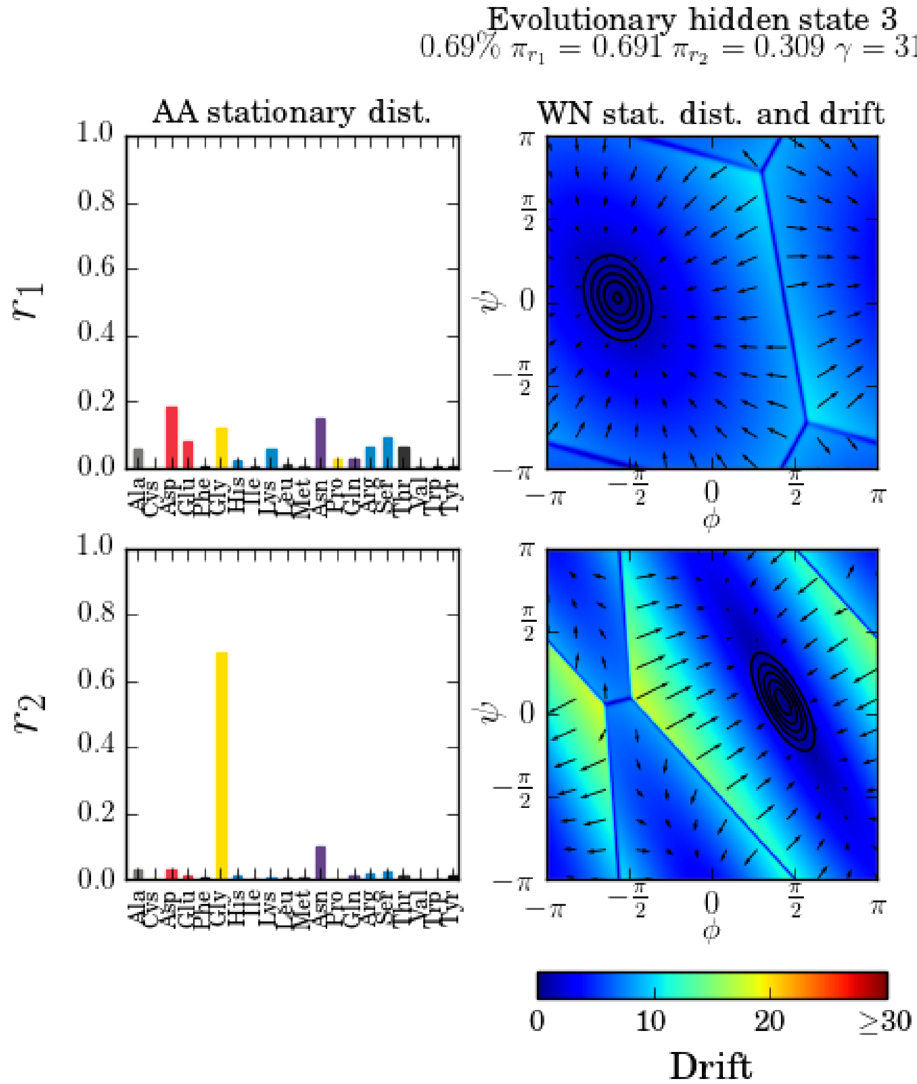
Evolutionary hidden state 3
0.69% $\pi_{r_1} = 0.691$ $\pi_{r_2} = 0.309$ $\gamma = 31.76$
AA stationary dist.
WN stat. dist. and drift
SS stationary dist.
r_1
r_2
1.0
0.8
0.6
0.4
0.2
0.0
Ala Cys Asp Glu Phe Gly His Ile Lys Leu Met Asn Pro Gln Arg Ser Thr Val Trp Tyr
ψ
ϕ
$-\pi$ $-\frac{\pi}{2}$ 0 $\frac{\pi}{2}$ π
H S C
0 10 20 ≥30
Drift

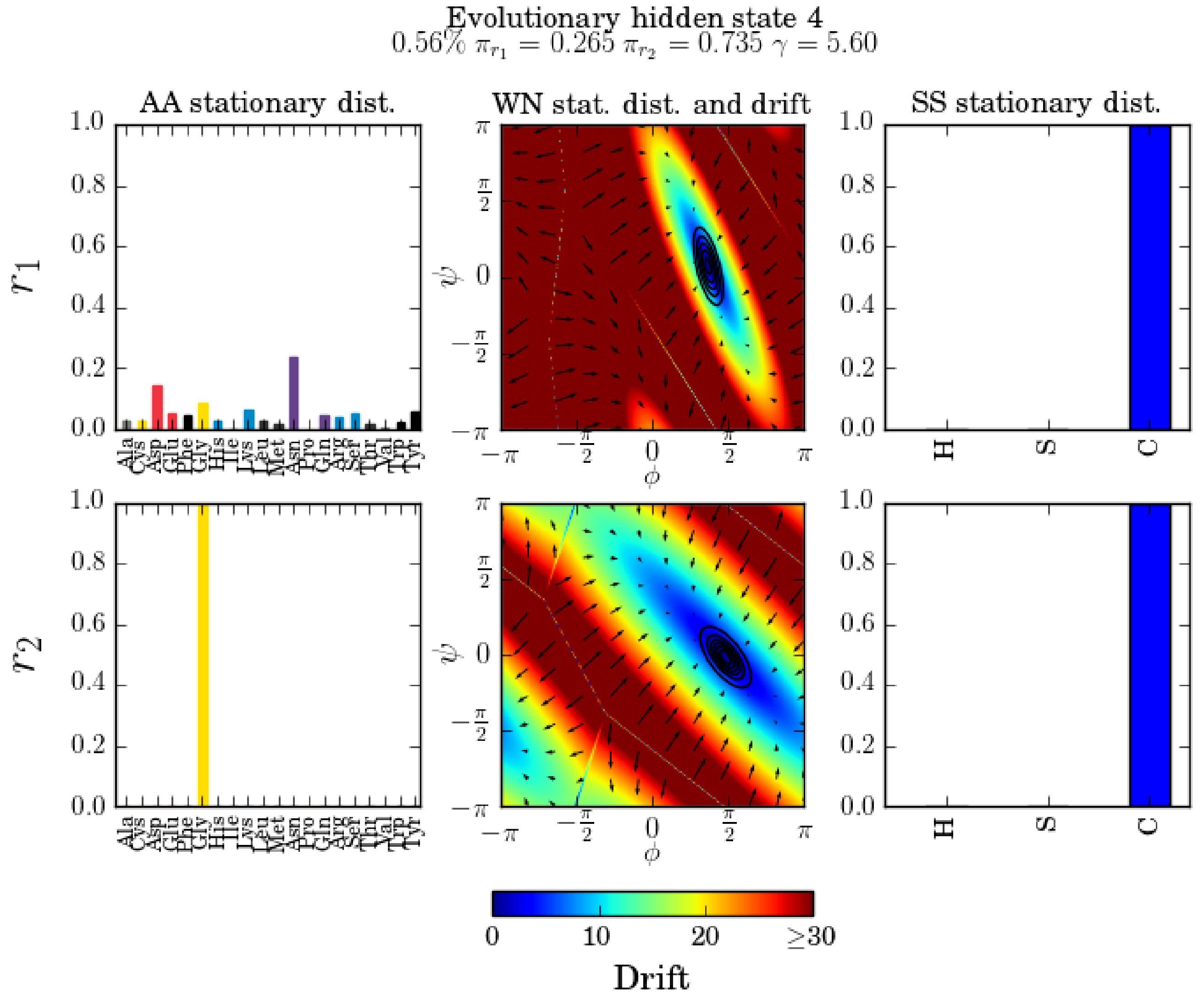

Evolutionary hidden state 4
0.56% $\pi_{r_1} = 0.265$ $\pi_{r_2} = 0.735$ $\gamma = 5.60$
AA stationary dist.
WN stat. dist. and drift
SS stationary dist.
r_1
r_2
Ala Cys Asp Glu Phe Gly His Ile Lys Leu Met Asn Pro Gln Arg Ser Thr Val Trp Tyr
ψ
ϕ
H S C
Drift
0 10 20 ≥30

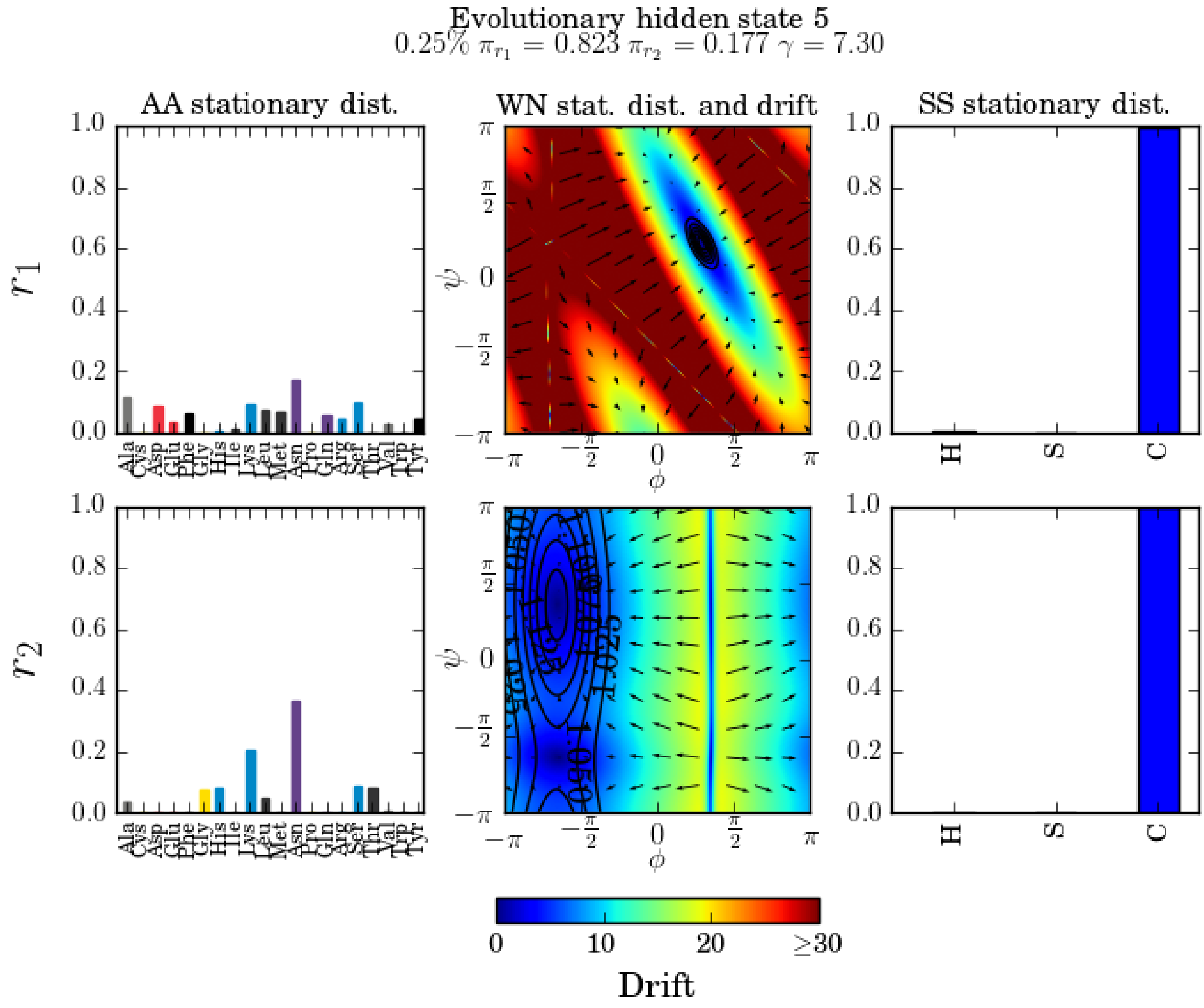
Evolutionary hidden state 5
0.25% $\pi_{r_1} = 0.823$ $\pi_{r_2} = 0.177$ $\gamma = 7.30$
AA stationary dist.
WN stat. dist. and drift
SS stationary dist.
r_1
r_2
Ala Cys Asp Glu Phe Gly His Ile Lys Leu Met Asn Pro Gln Arg Ser Thr Val Trp Tyr
ϕ
ψ
H S C
Drift
0 10 20 ≥30

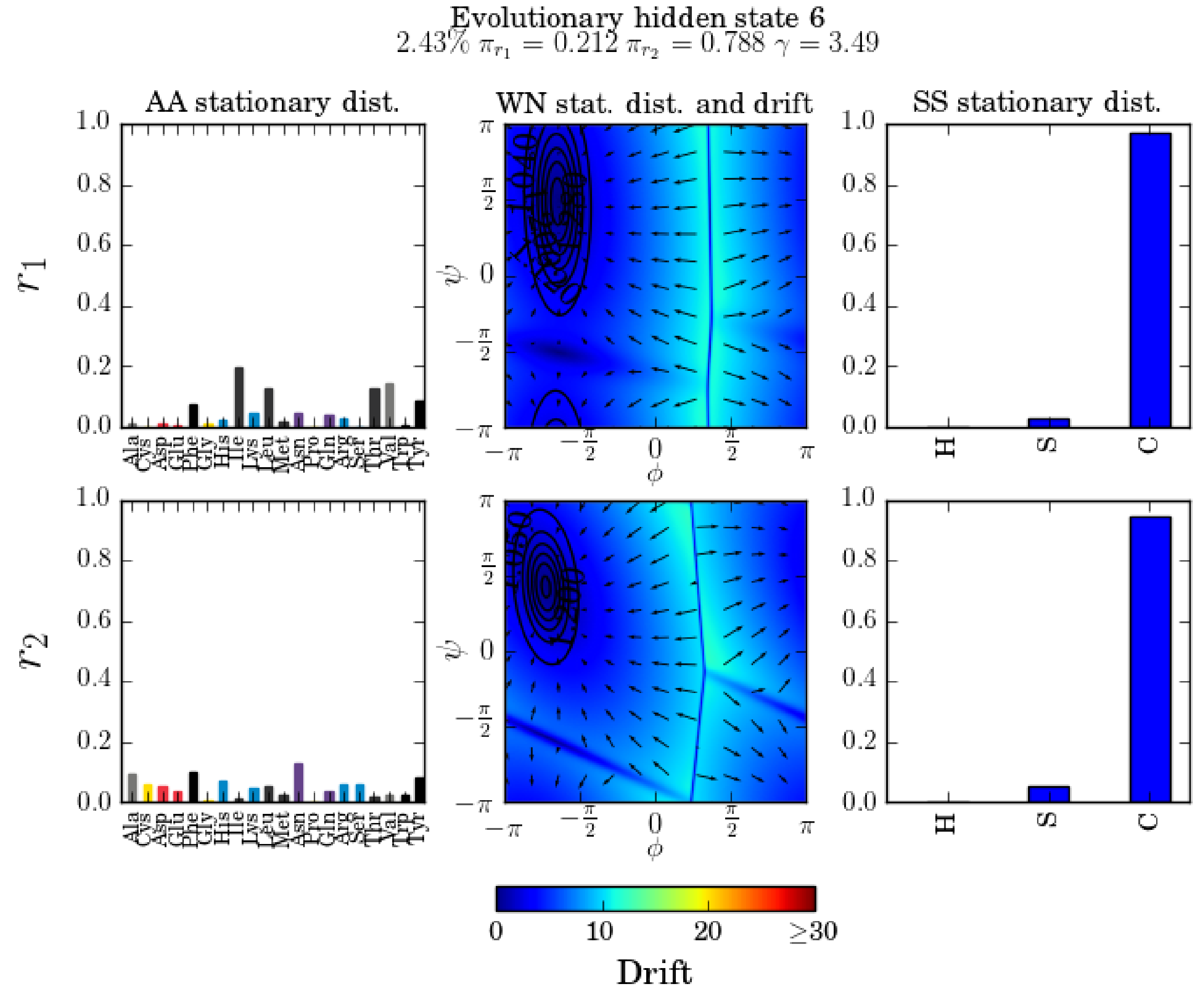

Evolutionary hidden state 6
2.43% $\pi_{r_1} = 0.212$ $\pi_{r_2} = 0.788$ $\gamma = 3.49$
AA stationary dist.
WN stat. dist. and drift
SS stationary dist.
r_1
r_2
ψ
ϕ
Ala Cys Asp Glu Phe Gly His Ile Lys Leu Met Asn Pro Gln Arg Ser Thr Val Trp Tyr
H S C
0 10 20 ≥30
Drift

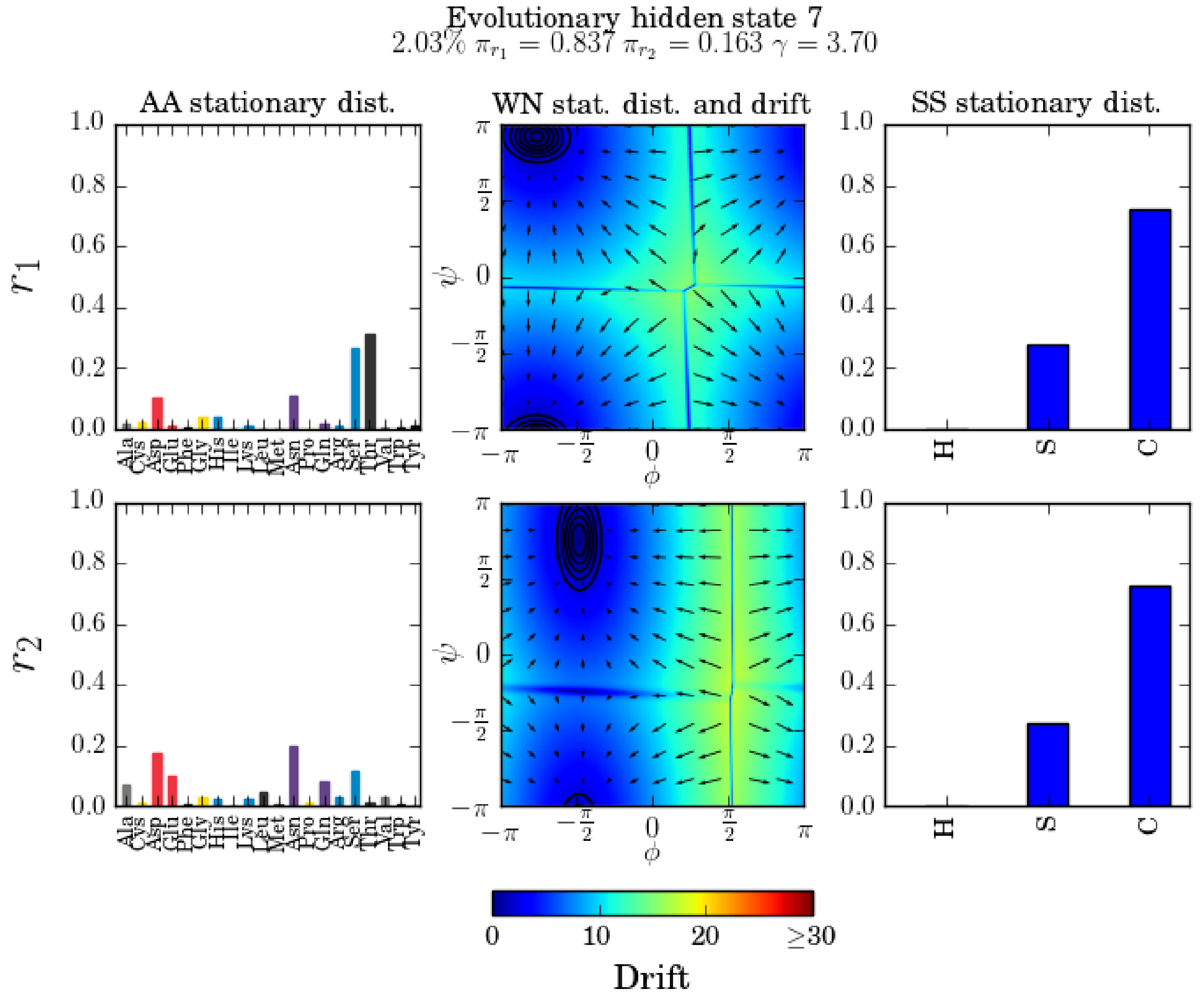

Evolutionary hidden state 7
2.03% $\pi_{r_1} = 0.837$ $\pi_{r_2} = 0.163$ $\gamma = 3.70$
AA stationary dist.
WN stat. dist. and drift
SS stationary dist.
r_1
r_2
Ala
Cys
Asp
Glu
Phe
Gly
His
Ile
Lys
Leu
Met
Asn
Pro
Gln
Arg
Ser
Thr
Val
Trp
Tyr
ψ
ϕ
H
S
C
Drift
0
10
20
≥30

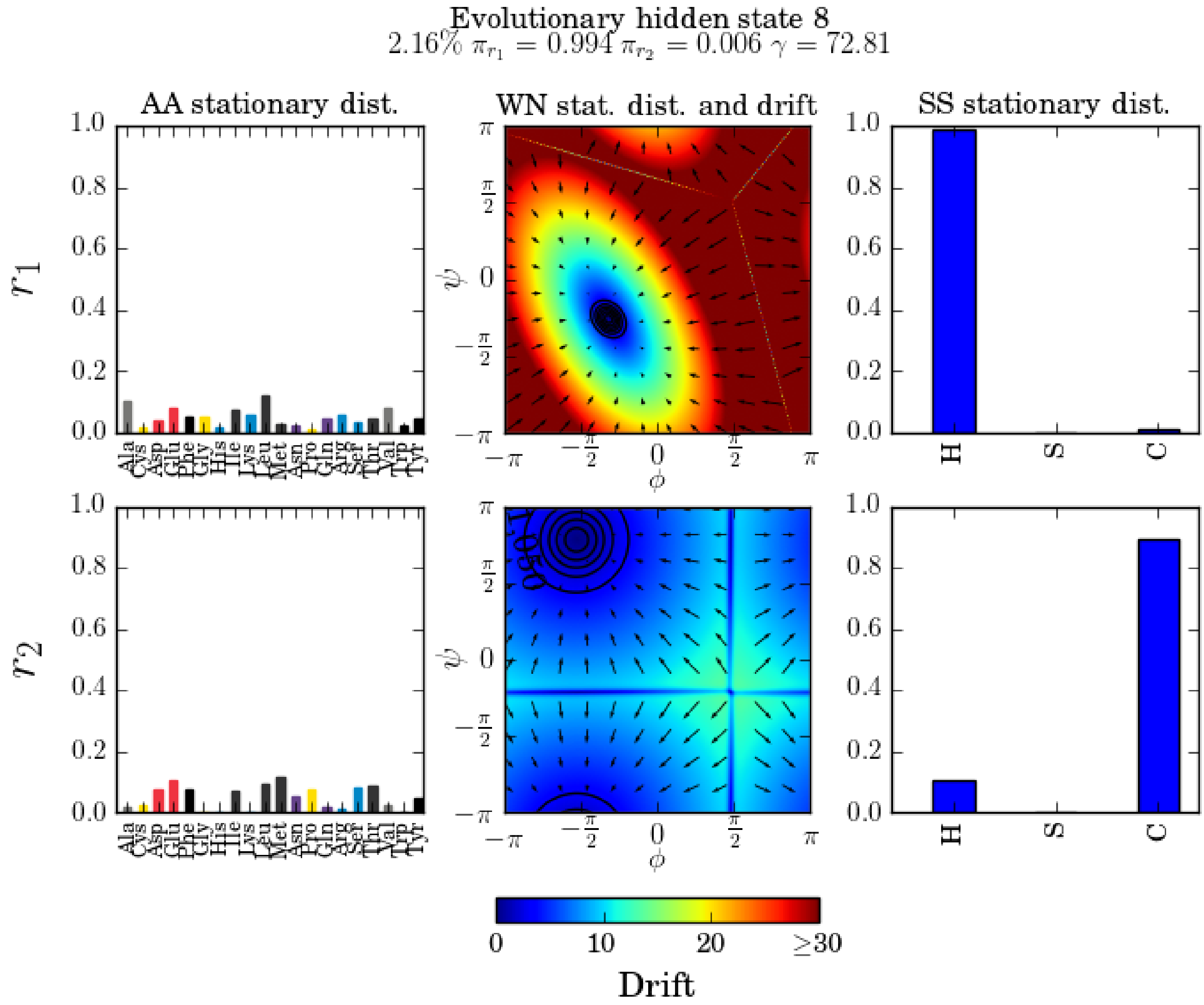
Evolutionary hidden state 8
2.16% $\pi_{r_1} = 0.994$ $\pi_{r_2} = 0.006$ $\gamma = 72.81$
AA stationary dist.
WN stat. dist. and drift
SS stationary dist.
r_1
r_2
1.0
0.8
0.6
0.4
0.2
0.0
Ala Cys Asp Glu Phe Gly His Ile Lys Leu Met Asn Pro Gln Arg Ser Thr Val Trp Tyr
ψ
ϕ
π
$\frac{\pi}{2}$
0
$-\frac{\pi}{2}$
$-\pi$
H
S
C
0
10
20
≥30
Drift

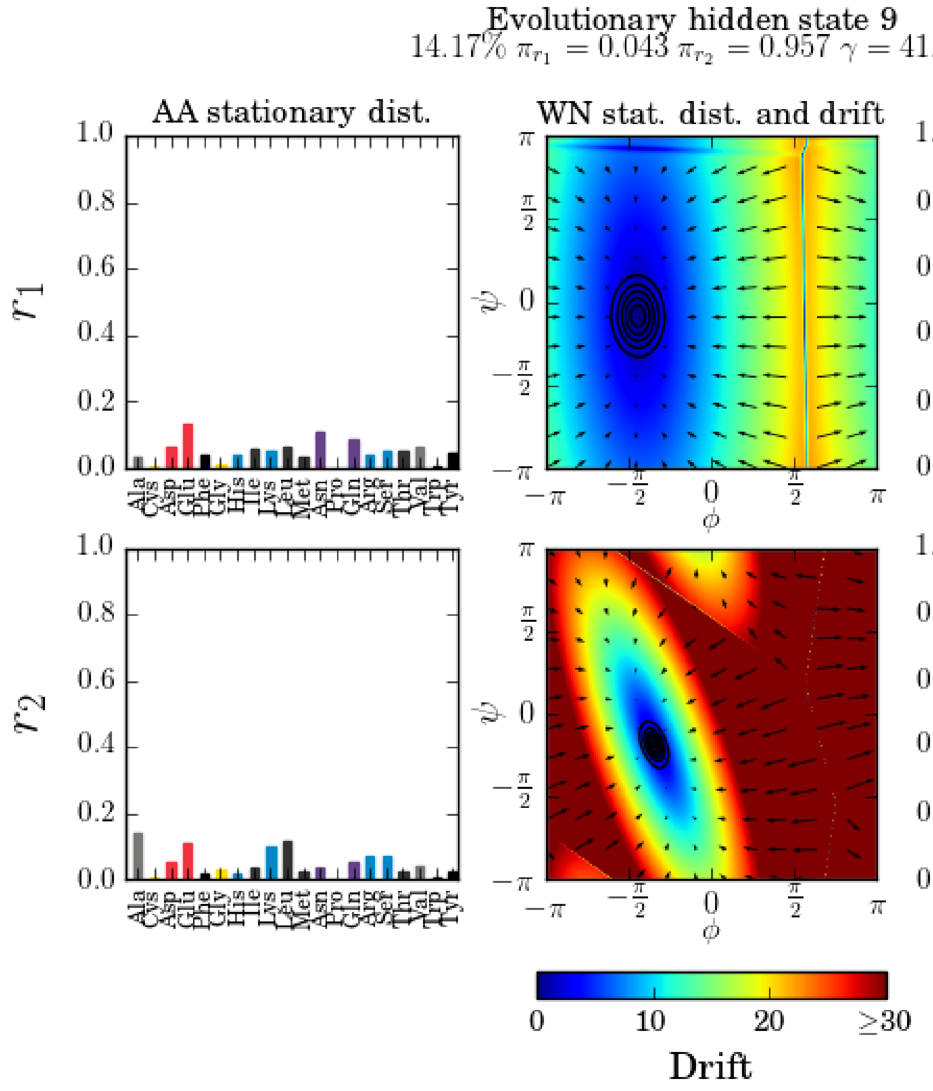

Evolutionary hidden state 9
14.17% $\pi_{r_1} = 0.043$ $\pi_{r_2} = 0.957$ $\gamma = 41.52$
AA stationary dist.
WN stat. dist. and drift
SS stationary dist.
r_1
r_2
Ala Cys Asp Glu Phe Gly His Ile Lys Leu Met Asn Pro Gln Arg Ser Thr Val Trp Tyr
ϕ
ψ
H
S
C
Drift
0
10
20
≥30

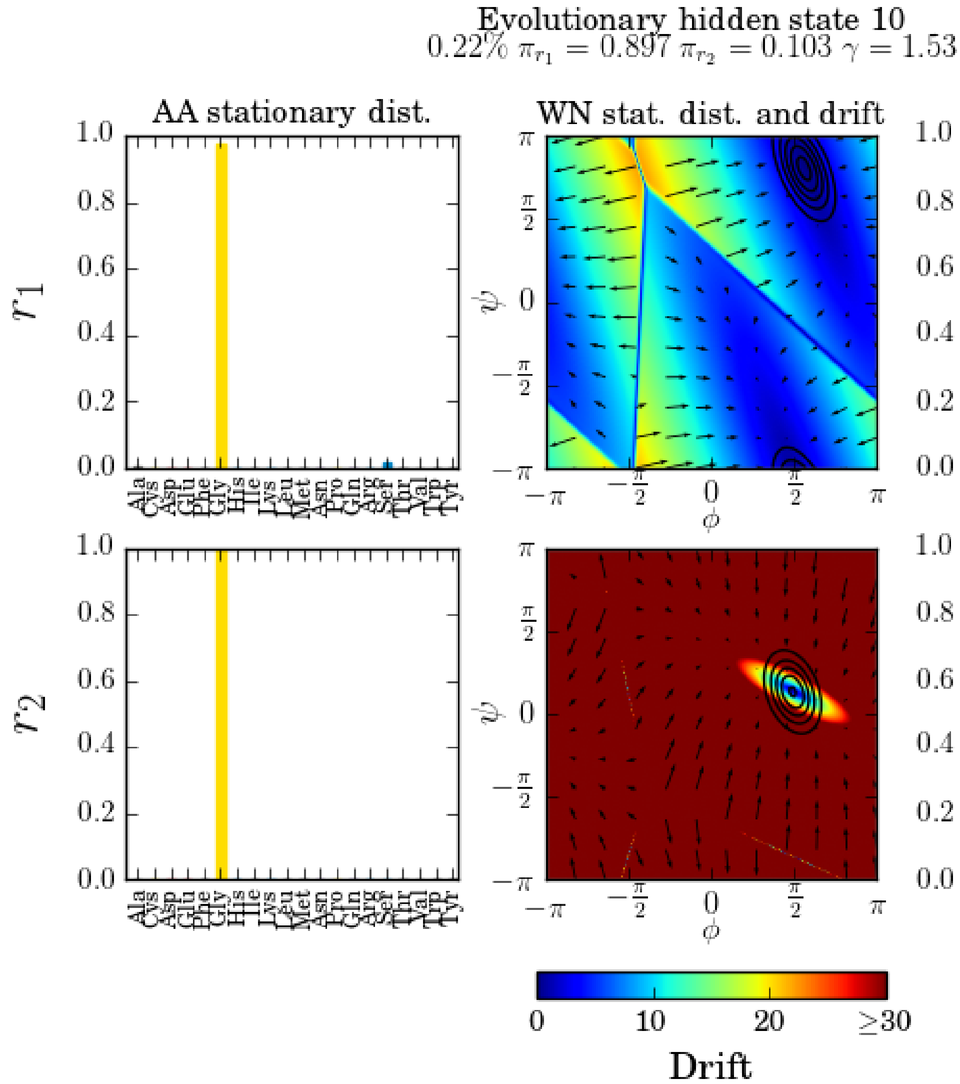

Evolutionary hidden state 10
0.22% $\pi_{r_1} = 0.897$ $\pi_{r_2} = 0.103$ $\gamma = 1.53$
AA stationary dist.
WN stat. dist. and drift
SS stationary dist.
r_1
r_2
Ala Cys Asp Glu Phe Gly His Ile Lys Leu Met Asn Pro Gln Arg Ser Thr Val Trp Tyr
ϕ
ψ
H S C
Drift
0 10 20 ≥30

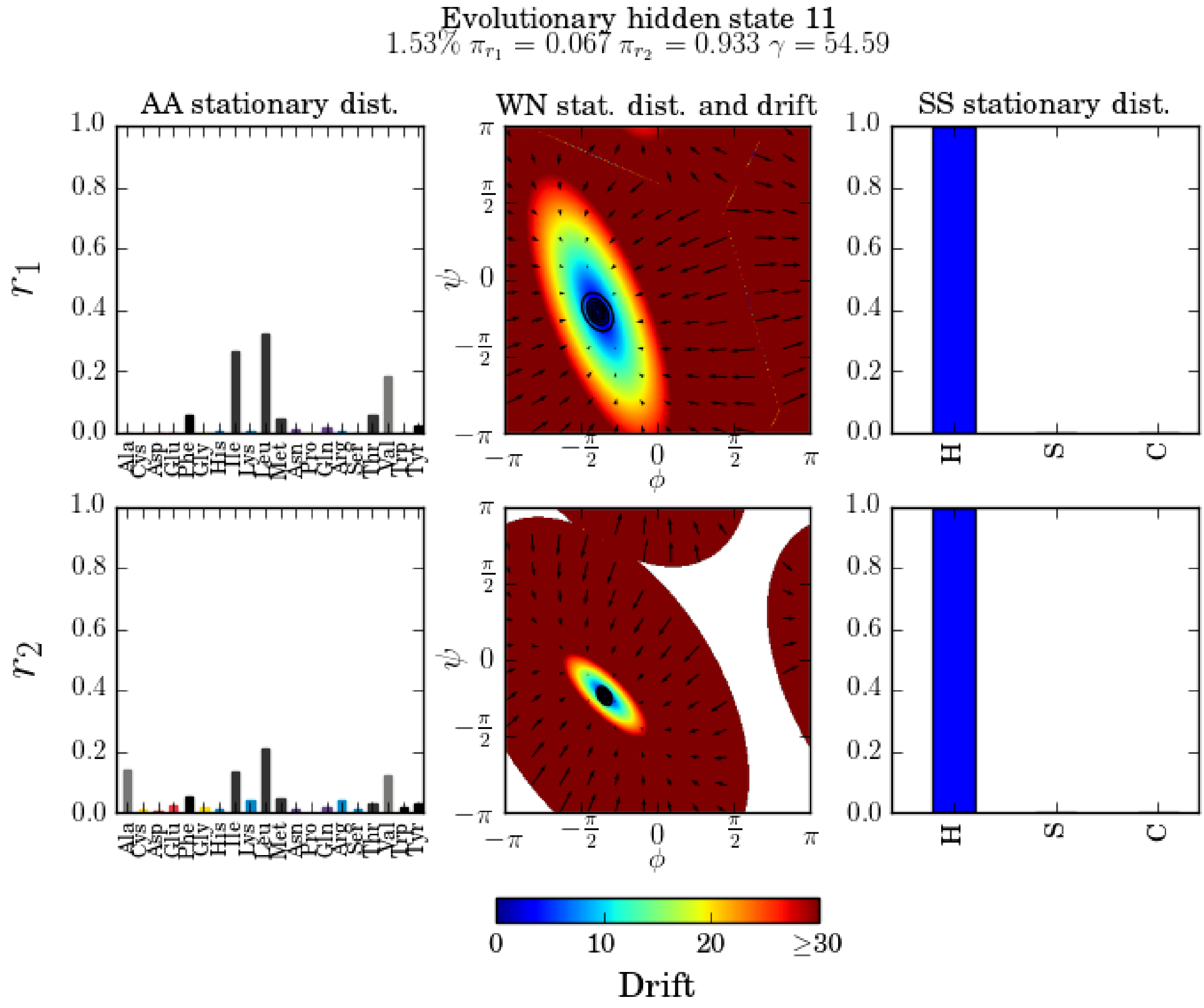

Evolutionary hidden state 11
1.53% $\pi_{r_1} = 0.067$ $\pi_{r_2} = 0.933$ $\gamma = 54.59$
AA stationary dist.
WN stat. dist. and drift
SS stationary dist.
r_1
r_2
Ala Cys Asp Glu Phe Gly His Ile Lys Leu Met Asn Pro Gln Arg Ser Thr Val Trp Tyr
H S C
ϕ
ψ
Drift
0 10 20 ≥30

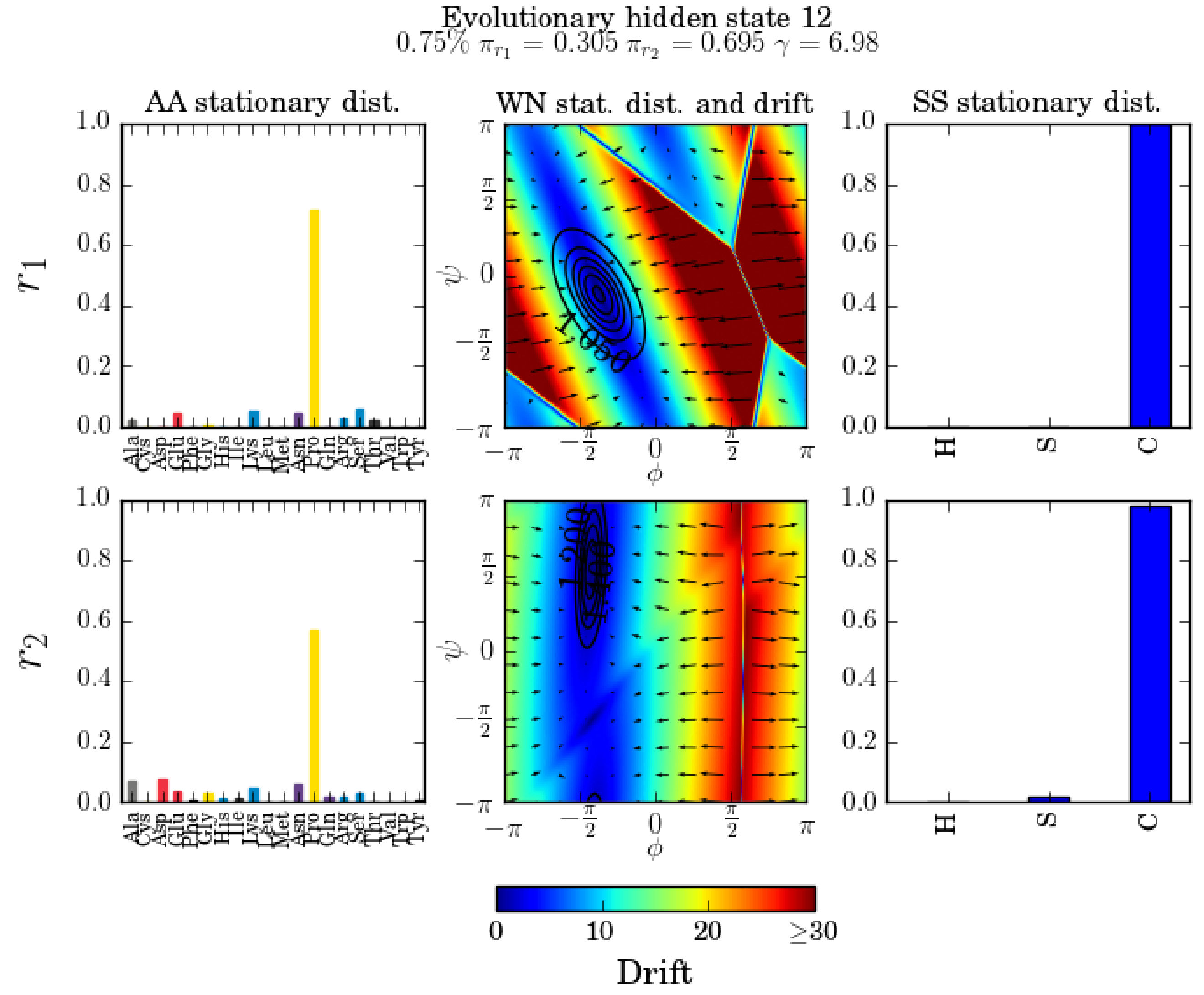

Evolutionary hidden state 12
0.75% $\pi_{r_1} = 0.305$ $\pi_{r_2} = 0.695$ $\gamma = 6.98$
AA stationary dist.
WN stat. dist. and drift
SS stationary dist.
r_1
r_2
Ala Cys Asp Glu Phe Gly His Ile Lys Leu Met Asn Pro Gln Arg Ser Thr Val Trp Tyr
ψ
ϕ
H
S
C
1.050
1.200
1.400
Drift
0
10
20
≥30

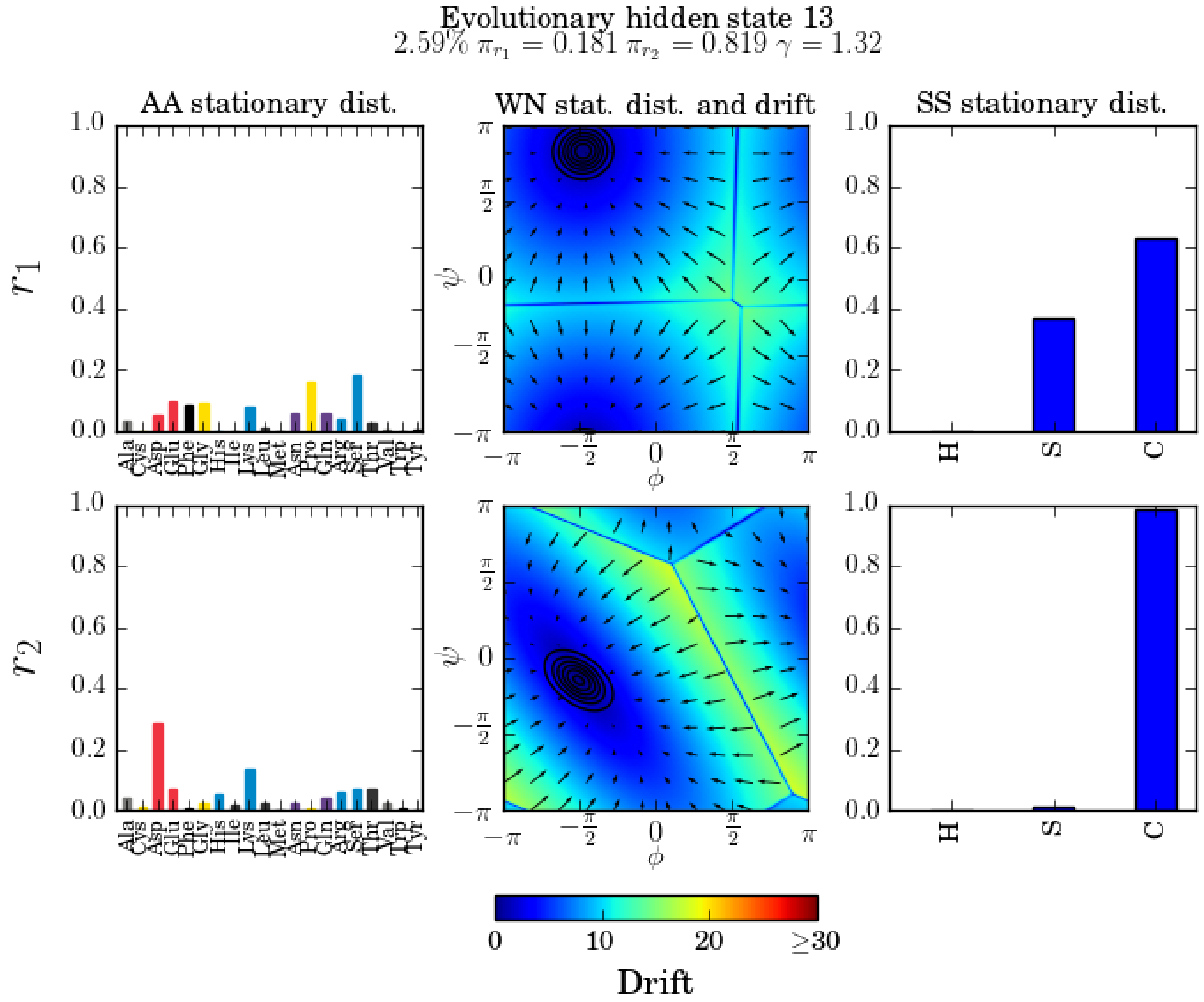
Evolutionary hidden state 13
2.59% $\pi_{r_1} = 0.181$ $\pi_{r_2} = 0.819$ $\gamma = 1.32$
AA stationary dist.
WN stat. dist. and drift
SS stationary dist.
r_1
r_2
Ala Cys Asp Glu Phe Gly His Ile Lys Leu Met Asn Pro Gln Arg Ser Thr Val Trp Tyr
H S C
ϕ
ψ
$-\pi$ $-\frac{\pi}{2}$ 0 $\frac{\pi}{2}$ π
0.0 0.2 0.4 0.6 0.8 1.0
0 10 20 ≥30
Drift

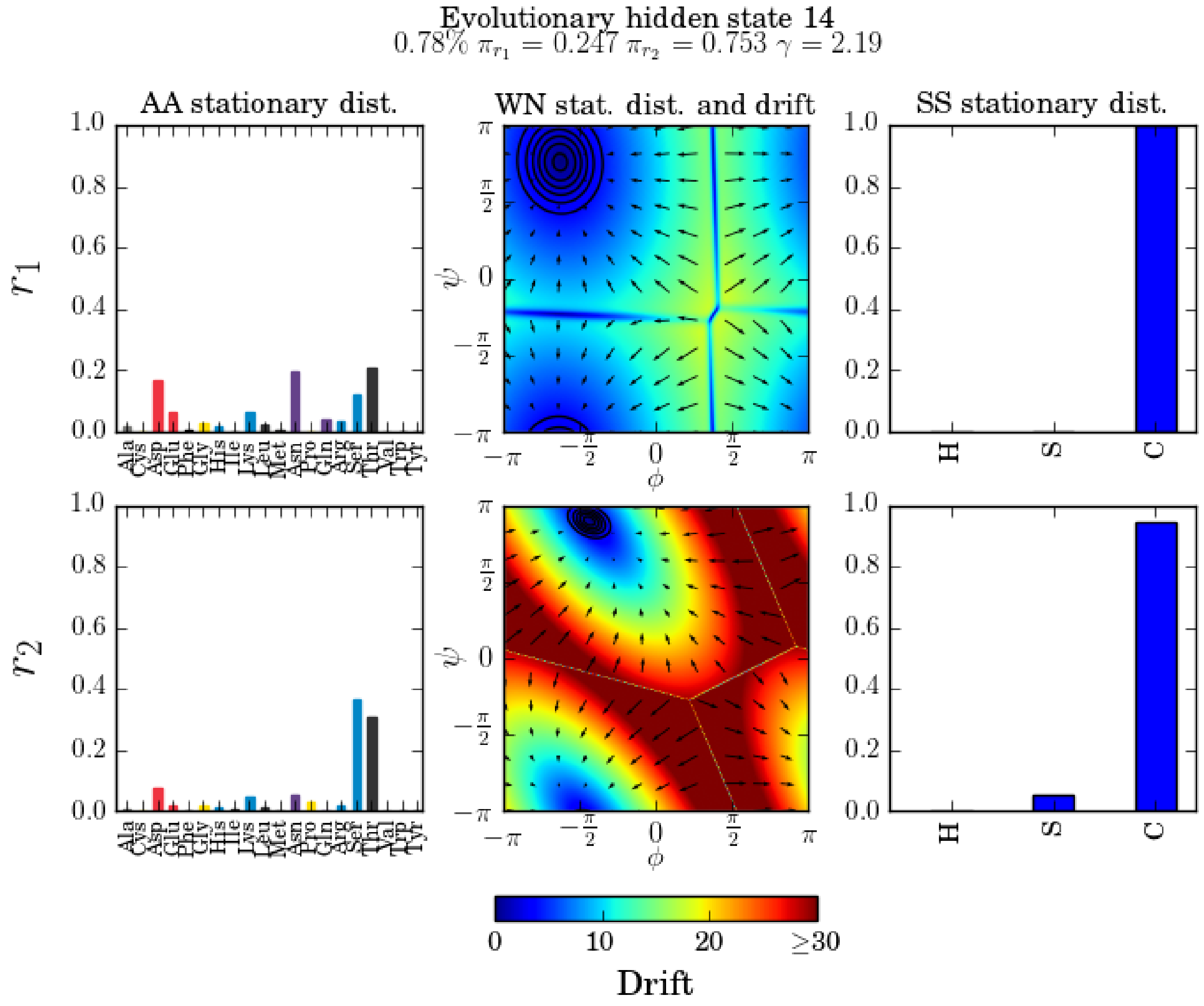

Evolutionary hidden state 14
0.78% $\pi_{r_1} = 0.247$ $\pi_{r_2} = 0.753$ $\gamma = 2.19$
AA stationary dist.
WN stat. dist. and drift
SS stationary dist.
r_1
r_2
1.0
0.8
0.6
0.4
0.2
0.0
Ala Cys Asp Glu Phe Gly His Ile Lys Leu Met Asn Pro Gln Arg Ser Thr Val Trp Tyr
π
$\frac{\pi}{2}$
0
$-\frac{\pi}{2}$
$-\pi$
ψ
ϕ
H
S
C
0
10
20
≥30
Drift

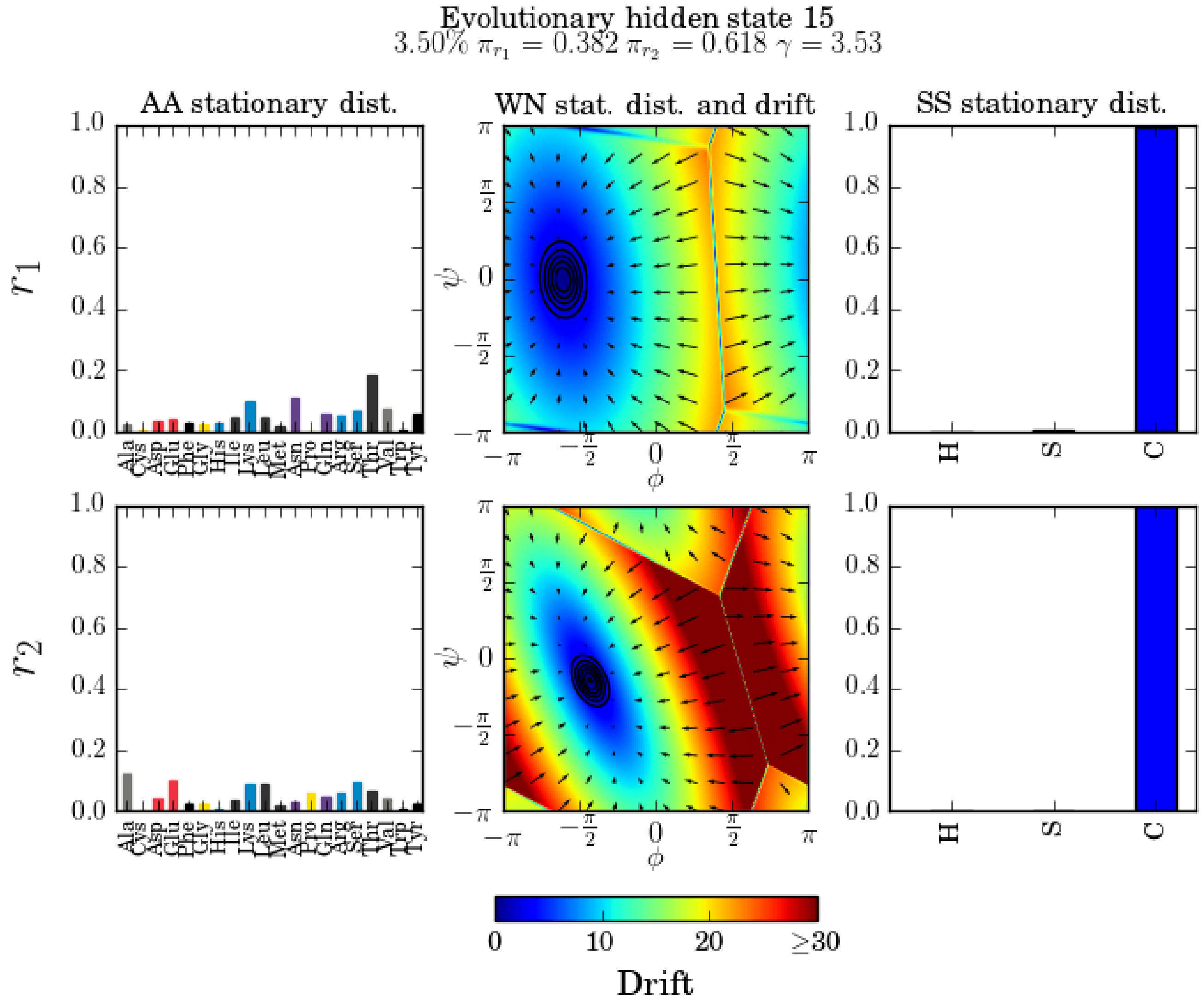

Evolutionary hidden state 15
3.50% $\pi_{r_1} = 0.382$ $\pi_{r_2} = 0.618$ $\gamma = 3.53$
AA stationary dist.
WN stat. dist. and drift
SS stationary dist.
r_1
r_2
Ala Cys Asp Glu Phe Gly His Ile Lys Leu Met Asn Pro Gln Arg Ser Thr Val Trp Tyr
H S C
ψ
ϕ
Drift
0 10 20 ≥30

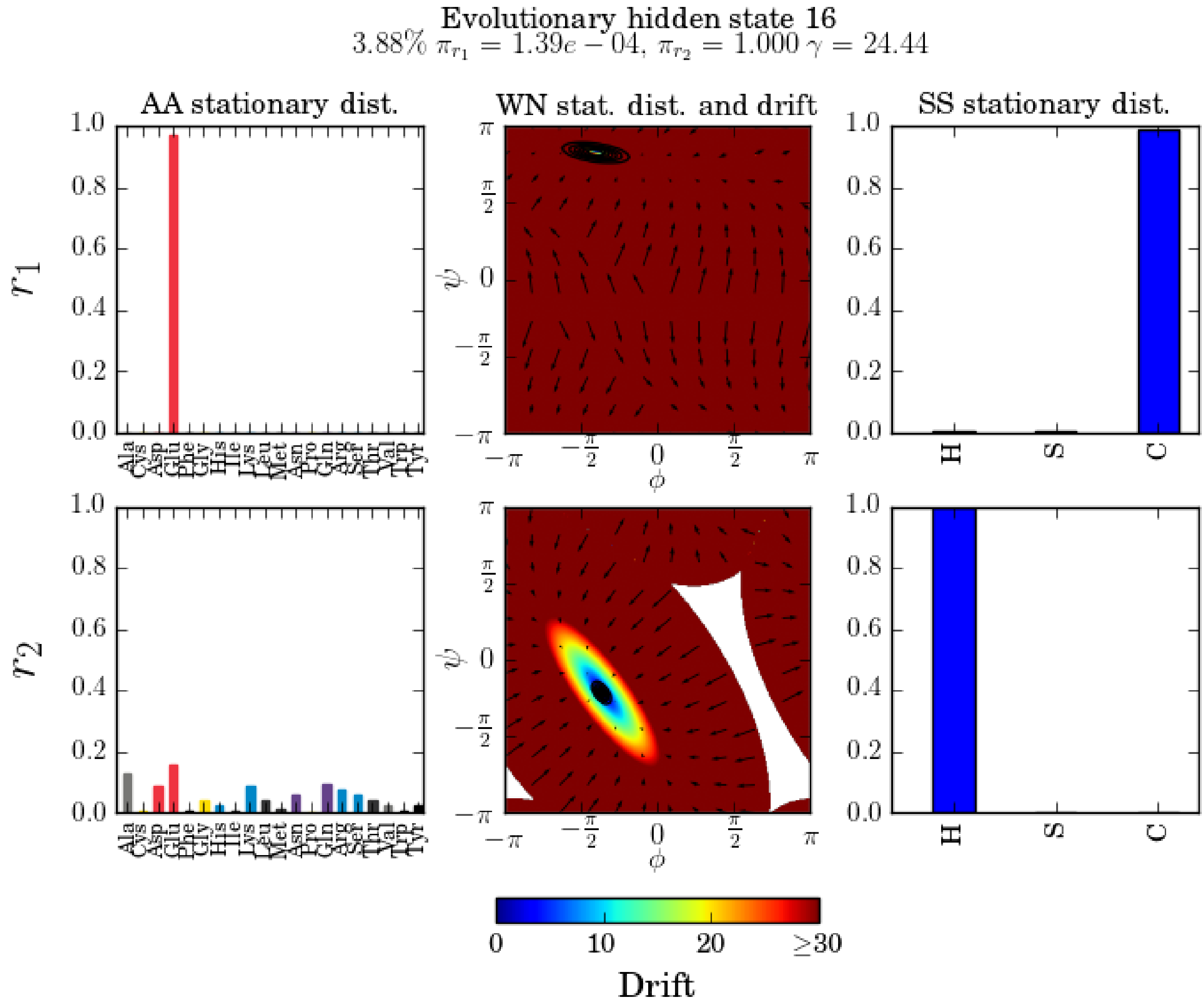
Evolutionary hidden state 16
3.88% $\pi_{r_1} = 1.39e-04$, $\pi_{r_2} = 1.000$ $\gamma = 24.44$
AA stationary dist.
WN stat. dist. and drift
SS stationary dist.
r_1
r_2
Ala Cys Asp Glu Phe Gly His Ile Lys Leu Met Asn Pro Gln Arg Ser Thr Val Trp Tyr
ψ
ϕ
H S C
Drift
0 10 20 ≥30

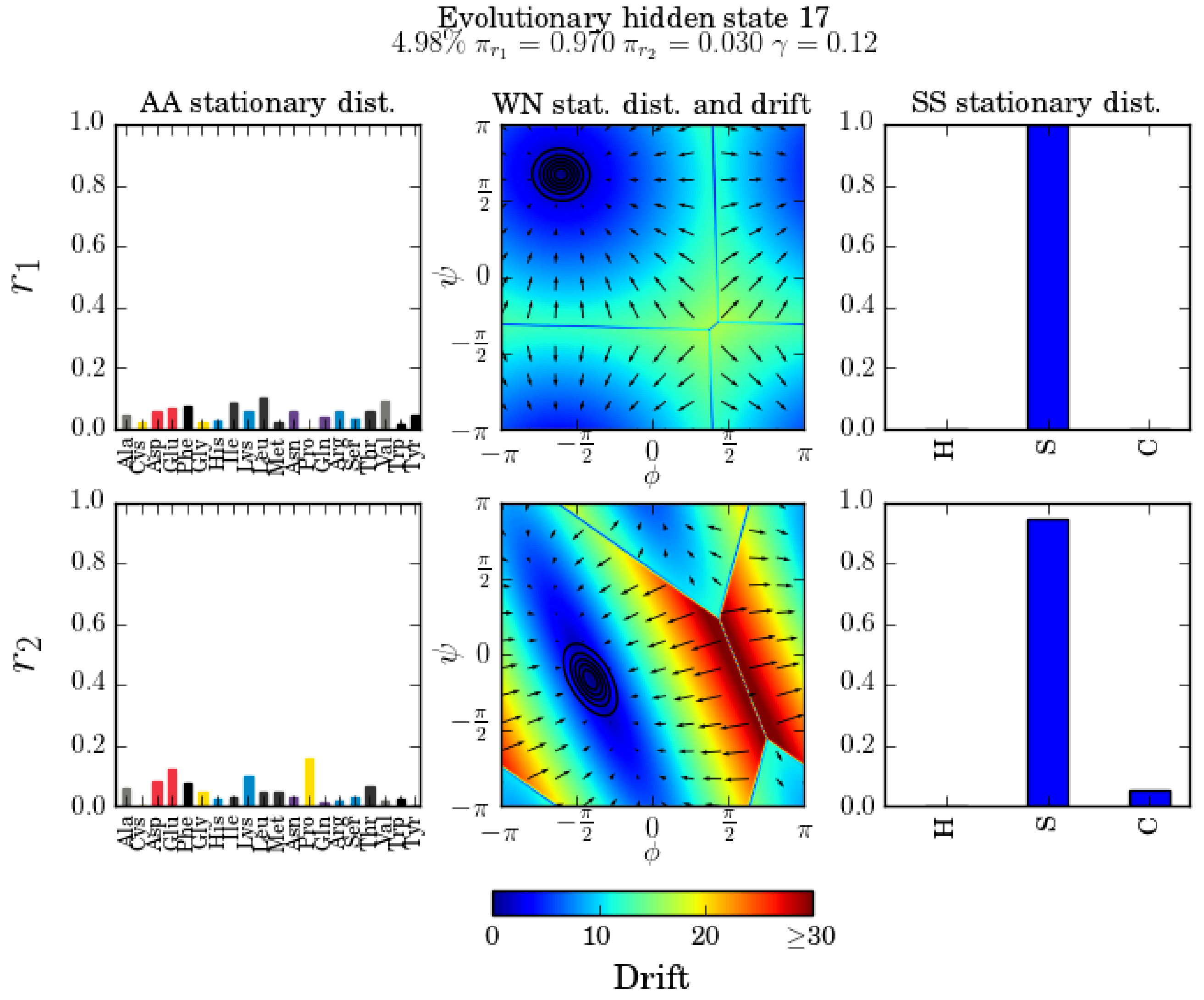

Evolutionary hidden state 17
4.98% $\pi_{r_1} = 0.970$ $\pi_{r_2} = 0.030$ $\gamma = 0.12$
AA stationary dist.
WN stat. dist. and drift
SS stationary dist.
r_1
r_2
Ala Cys Asp Glu Phe Gly His Ile Lys Leu Met Asn Pro Gln Arg Ser Thr Val Trp Tyr
ψ
ϕ
H S C
Drift
0 10 20 ≥30

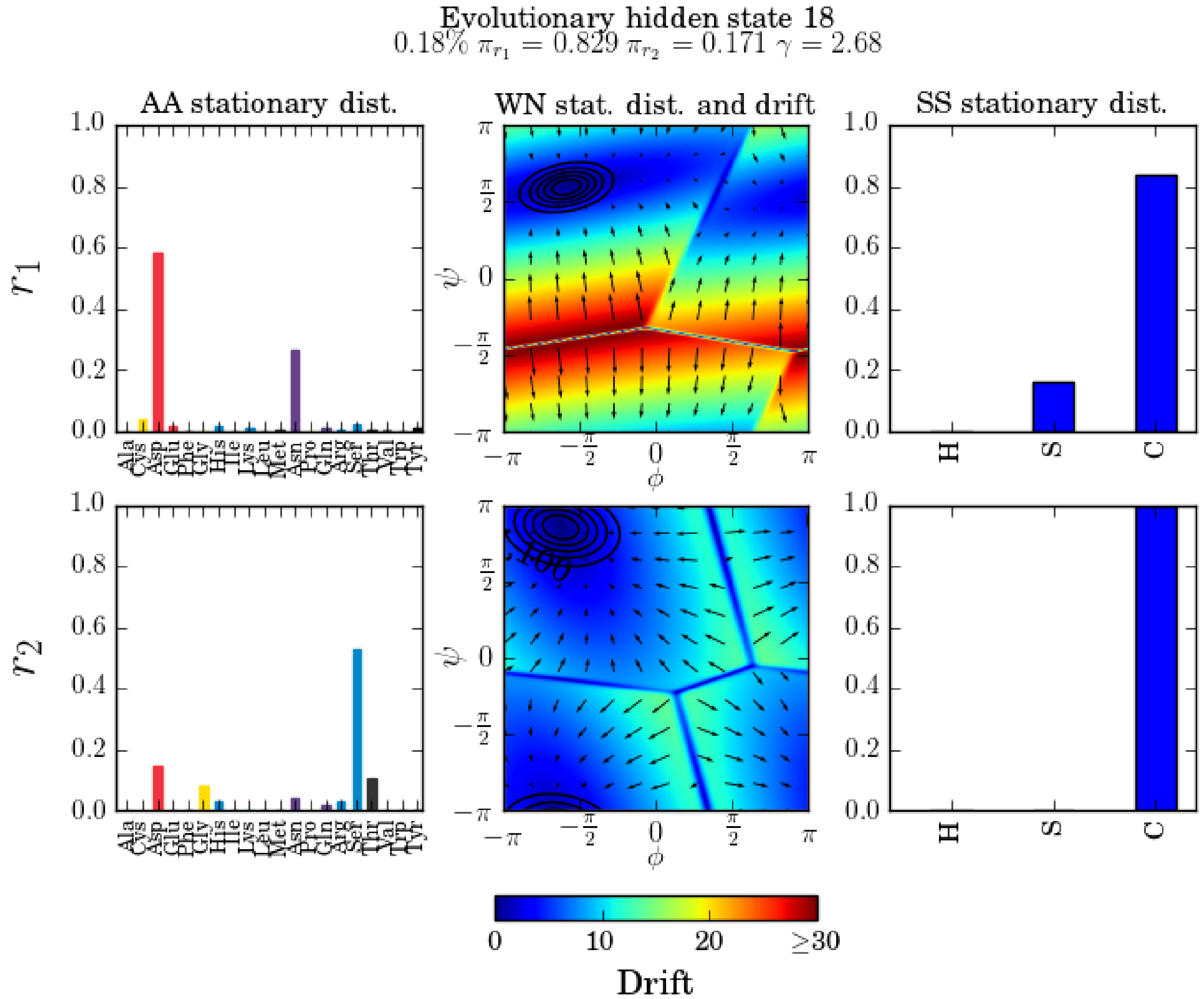

Evolutionary hidden state 18
$0.18\%\ \pi_{r_1} = 0.829\ \pi_{r_2} = 0.171\ \gamma = 2.68$
AA stationary dist.
WN stat. dist. and drift
SS stationary dist.
r_1
r_2
Ala
Cys
Asp
Glu
Phe
Gly
His
Ile
Lys
Leu
Met
Asn
Pro
Gln
Arg
Ser
Thr
Val
Trp
Tyr
ψ
ϕ
H
S
C
Drift
0
10
20
≥30

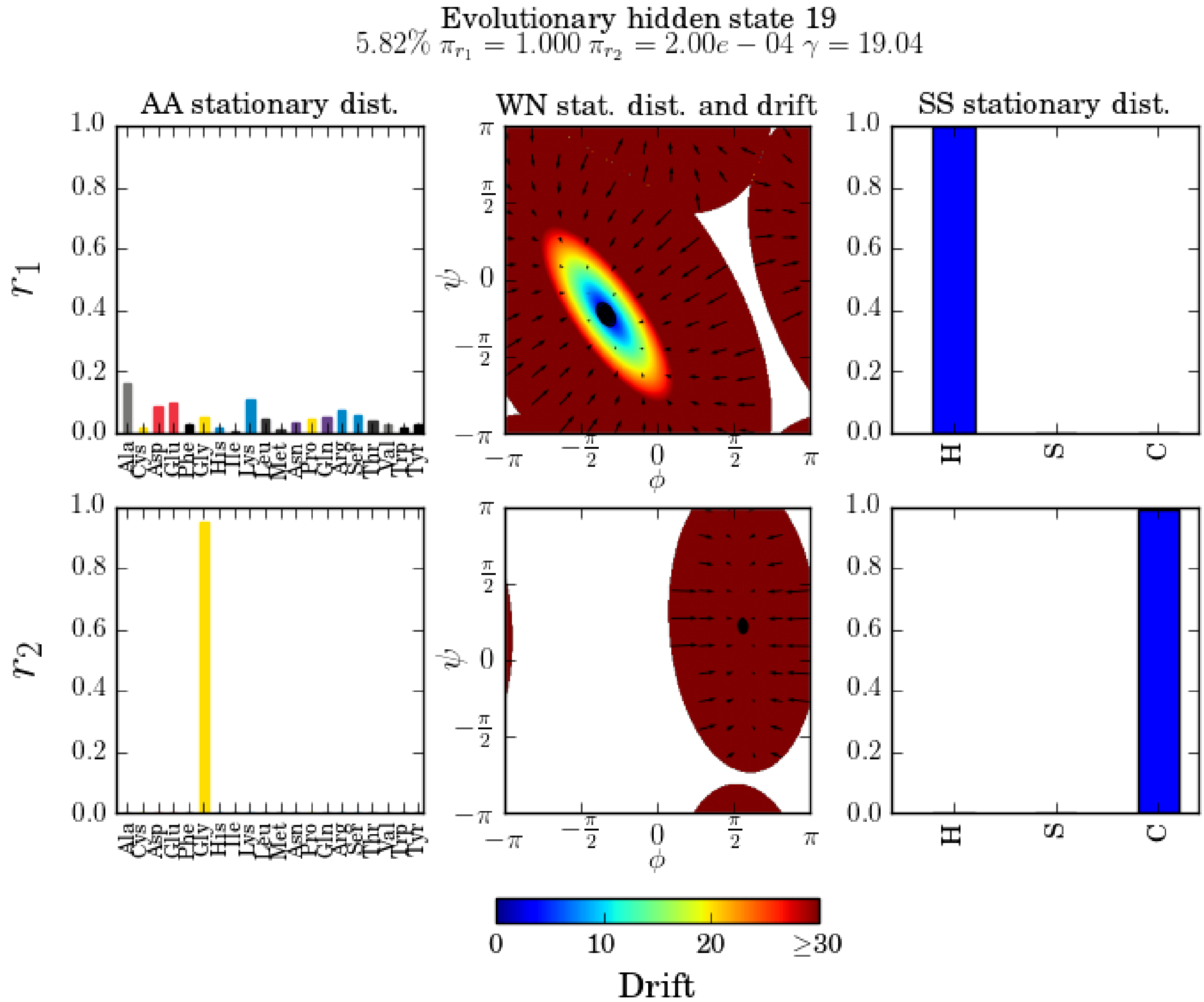

Evolutionary hidden state 19
5.82% $\pi_{r_1} = 1.000$ $\pi_{r_2} = 2.00e-04$ $\gamma = 19.04$
AA stationary dist.
WN stat. dist. and drift
SS stationary dist.
r_1
r_2
Ala Cys Asp Glu Phe Gly His Ile Lys Leu Met Asn Pro Gln Arg Ser Thr Val Trp Tyr
ψ
ϕ
H S C
Drift
0 10 20 ≥30

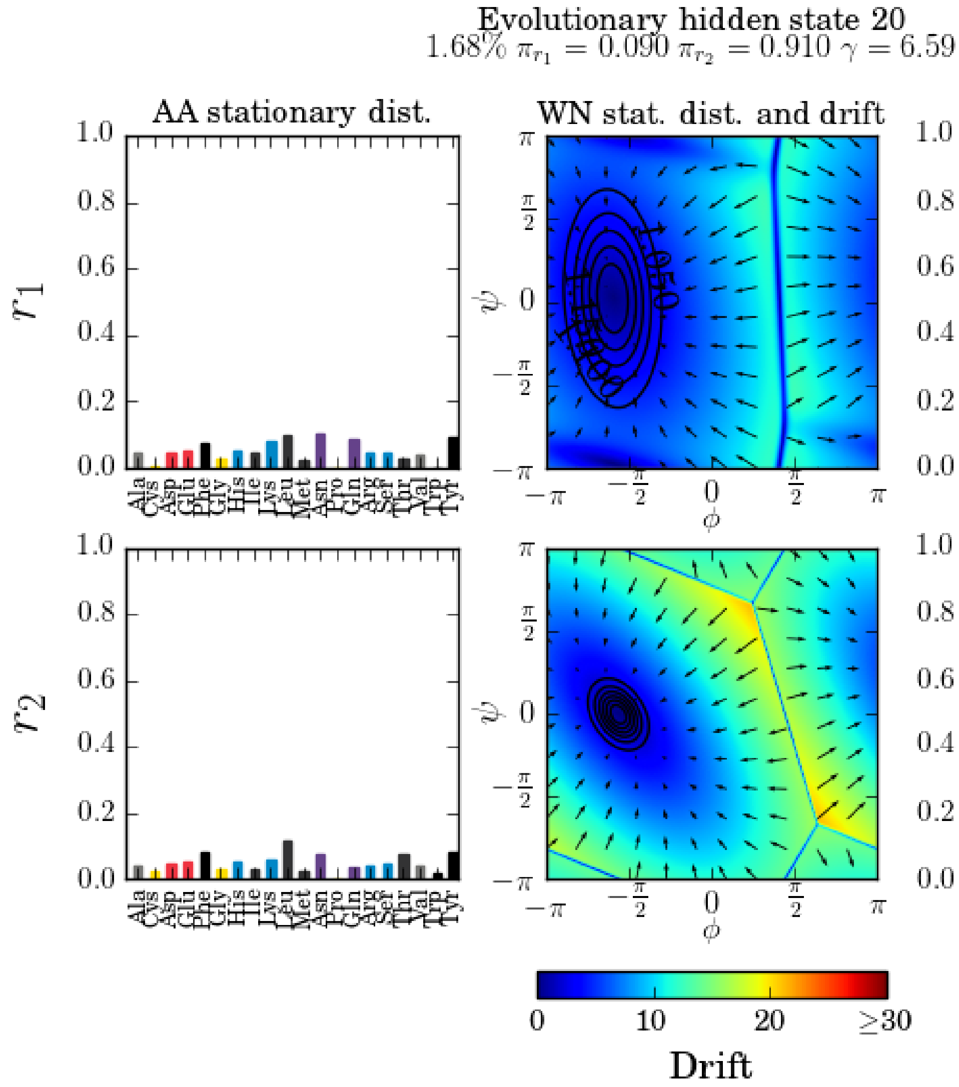

Evolutionary hidden state 20
1.68% $\pi_{r_1} = 0.090$ $\pi_{r_2} = 0.910$ $\gamma = 6.59$
AA stationary dist.
WN stat. dist. and drift
SS stationary dist.
r_1
r_2
Ala Cys Asp Glu Phe Gly His Ile Lys Leu Met Asn Pro Gln Arg Ser Thr Val Trp Tyr
ϕ
ψ
H S C
Drift
0 10 20 ≥30

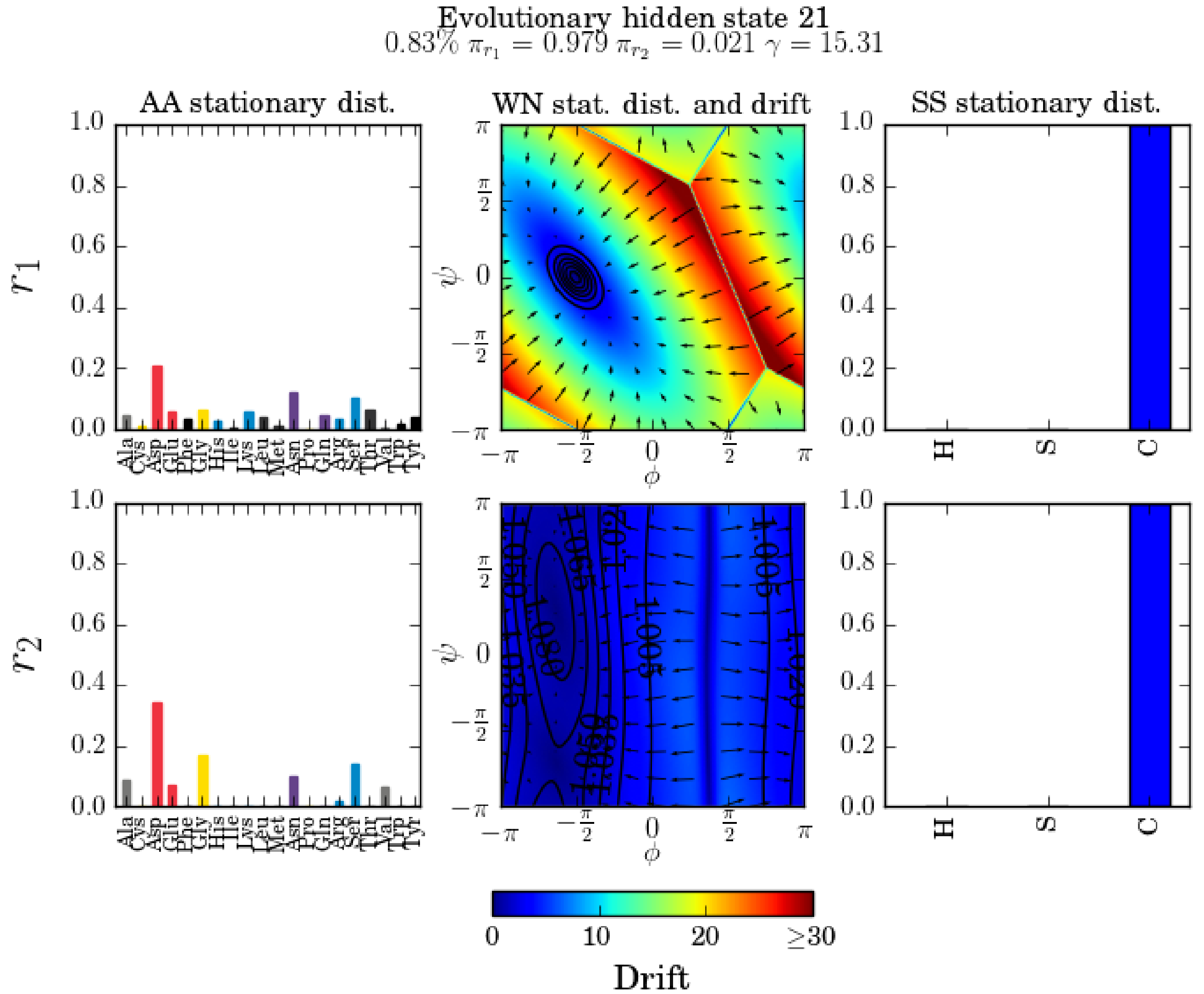

Evolutionary hidden state 21
0.83% $\pi_{r_1} = 0.979$ $\pi_{r_2} = 0.021$ $\gamma = 15.31$
AA stationary dist.
WN stat. dist. and drift
SS stationary dist.
r_1
r_2
Ala Cys Asp Glu Phe Gly His Ile Lys Leu Met Asn Pro Gln Arg Ser Thr Val Trp Tyr
H S C
ψ
ϕ
1.050
1.065
1.080
1.035
1.005
1.020
Drift
0 10 20 ≥30

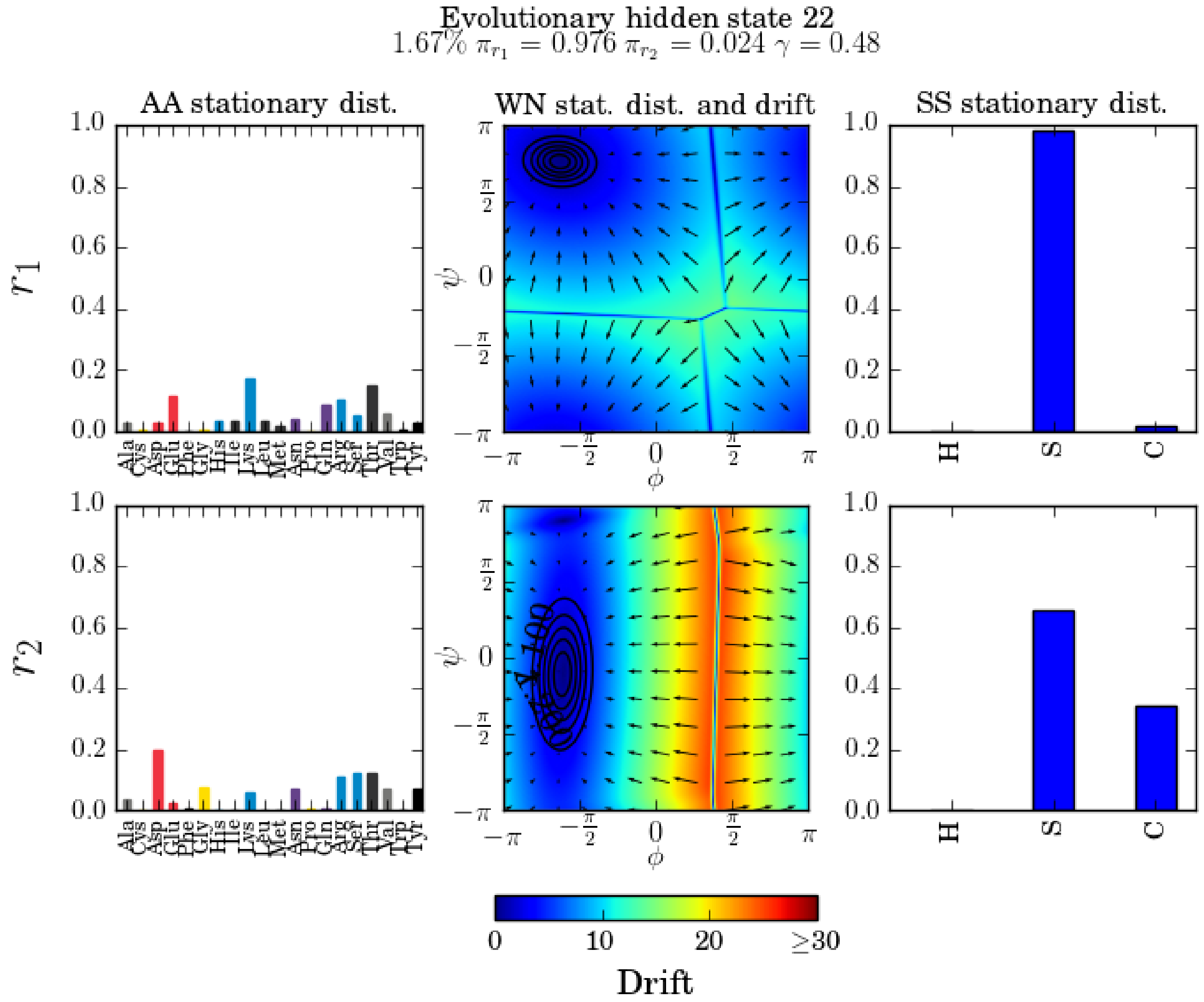

Evolutionary hidden state 22
1.67% $\pi_{r_1} = 0.976$ $\pi_{r_2} = 0.024$ $\gamma = 0.48$
AA stationary dist.
WN stat. dist. and drift
SS stationary dist.
r_1
r_2
1.0
0.8
0.6
0.4
0.2
0.0
Ala
Cys
Asp
Glu
Phe
Gly
His
Ile
Lys
Leu
Met
Asn
Pro
Gln
Arg
Ser
Thr
Val
Trp
Tyr
π
$\frac{\pi}{2}$
0
$-\frac{\pi}{2}$
$-\pi$
ψ
ϕ
H
S
C
0
10
20
≥30
Drift

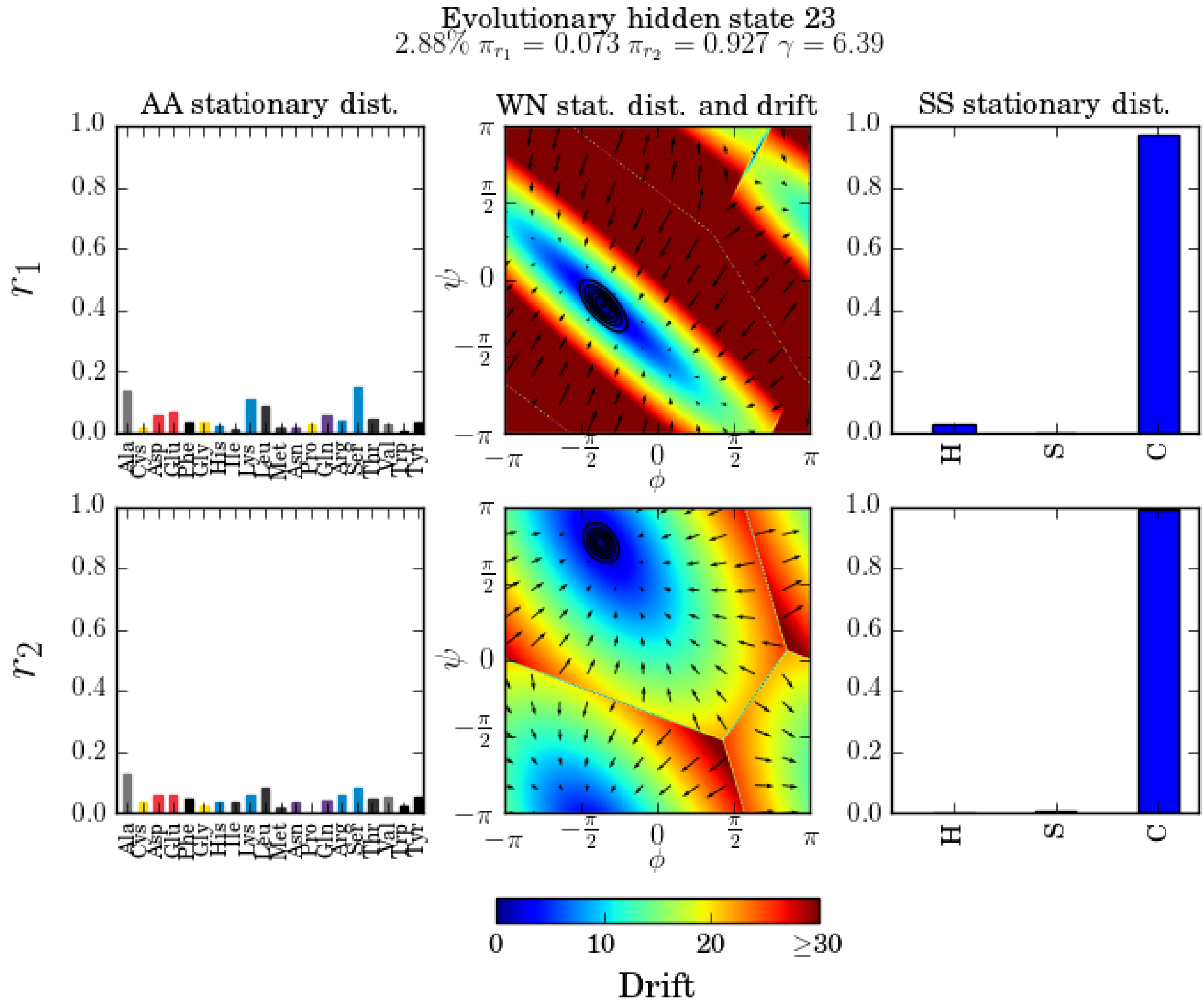

Evolutionary hidden state 23
2.88% $\pi_{r_1} = 0.073$ $\pi_{r_2} = 0.927$ $\gamma = 6.39$
AA stationary dist.
WN stat. dist. and drift
SS stationary dist.
r_1
r_2
Ala Cys Asp Glu Phe Gly His Ile Lys Leu Met Asn Pro Gln Arg Ser Thr Val Trp Tyr
ϕ
ψ
H
S
C
Drift
0
10
20
≥30

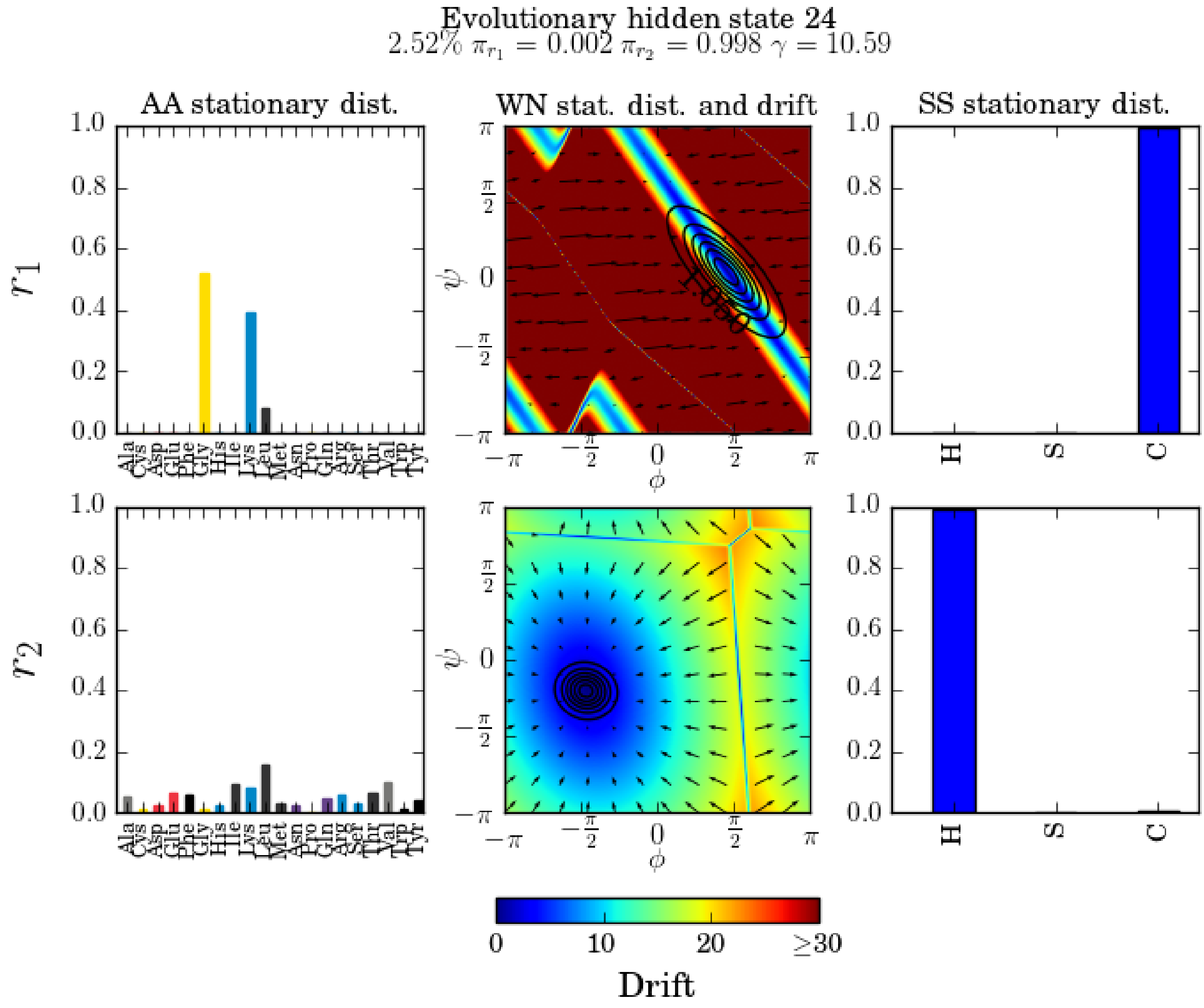
Evolutionary hidden state 24
2.52% $\pi_{r_1} = 0.002$ $\pi_{r_2} = 0.998$ $\gamma = 10.59$
AA stationary dist.
WN stat. dist. and drift
SS stationary dist.
r_1
r_2
Ala Cys Asp Glu Phe Gly His Ile Lys Leu Met Asn Pro Gln Arg Ser Thr Val Trp Tyr
ϕ
ψ
H S C
Drift
0 10 20 ≥30

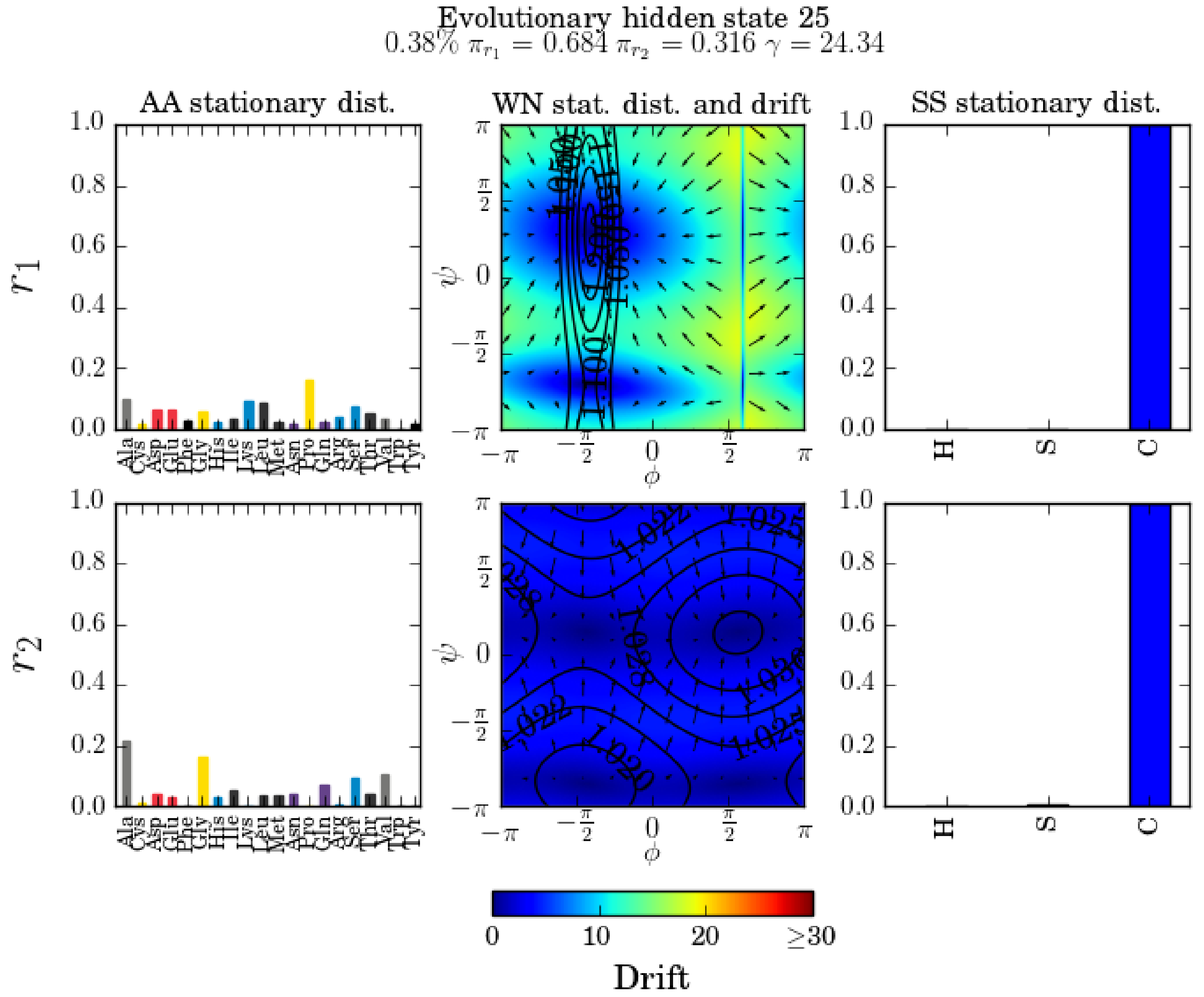
Evolutionary hidden state 25
0.38% $\pi_{r_1} = 0.684$ $\pi_{r_2} = 0.316$ $\gamma = 24.34$
AA stationary dist.
WN stat. dist. and drift
SS stationary dist.
r_1
r_2
1.0
0.8
0.6
0.4
0.2
0.0
Ala
Cys
Asp
Glu
Phe
Gly
His
Ile
Lys
Leu
Met
Asn
Pro
Gln
Arg
Ser
Thr
Val
Trp
Tyr
ψ
ϕ
π
$\frac{\pi}{2}$
0
$-\frac{\pi}{2}$
$-\pi$
H
S
C
1.022
1.025
1.020
1.030
0
10
20
≥30
Drift

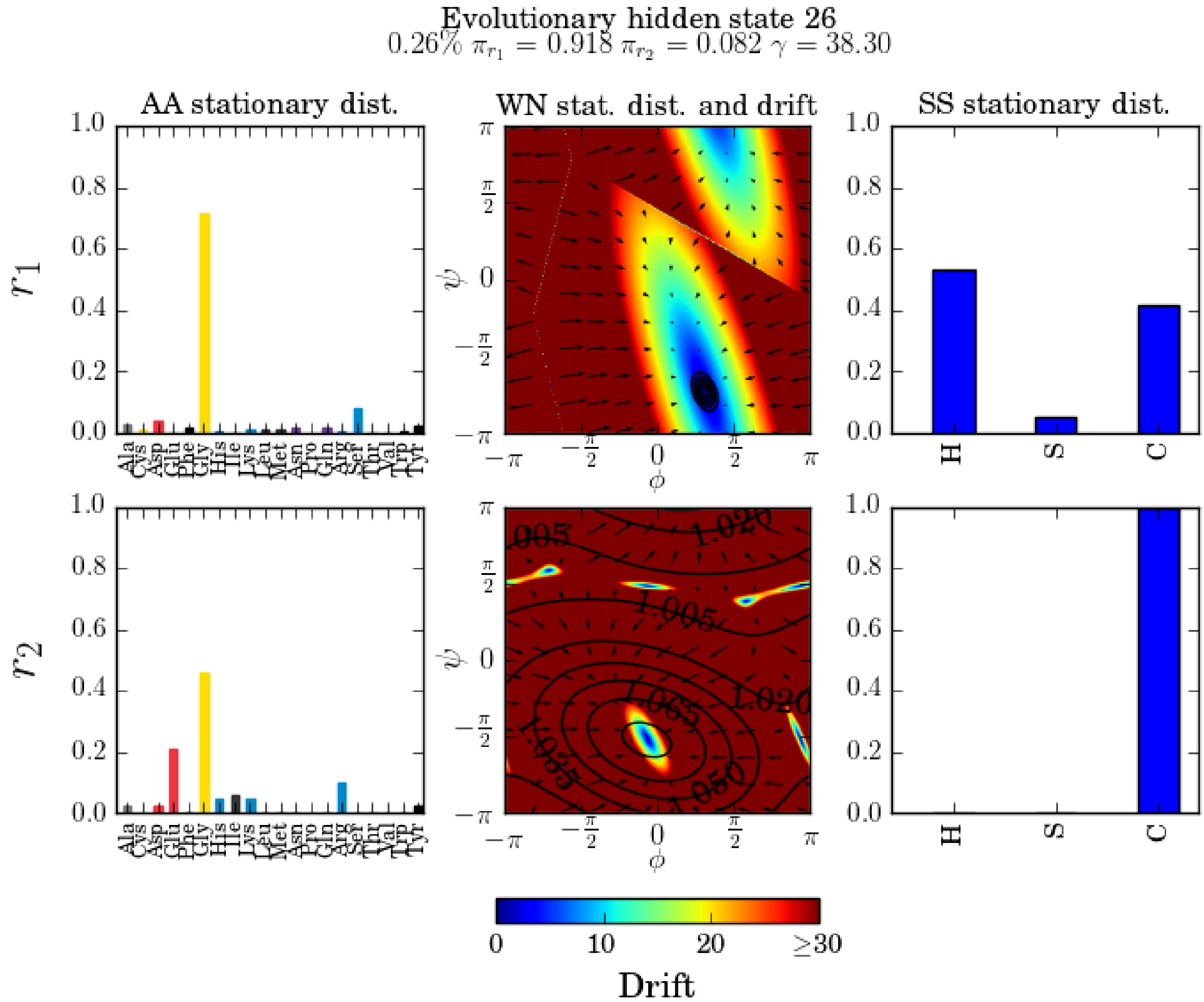
Evolutionary hidden state 26
0.26% $\pi_{r_1} = 0.918$ $\pi_{r_2} = 0.082$ $\gamma = 38.30$
AA stationary dist.
WN stat. dist. and drift
SS stationary dist.
r_1
r_2
ψ
ϕ
Ala Cys Asp Glu Phe Gly His Ile Lys Leu Met Asn Pro Gln Arg Ser Thr Val Trp Tyr
H S C
Drift
0 10 20 ≥30

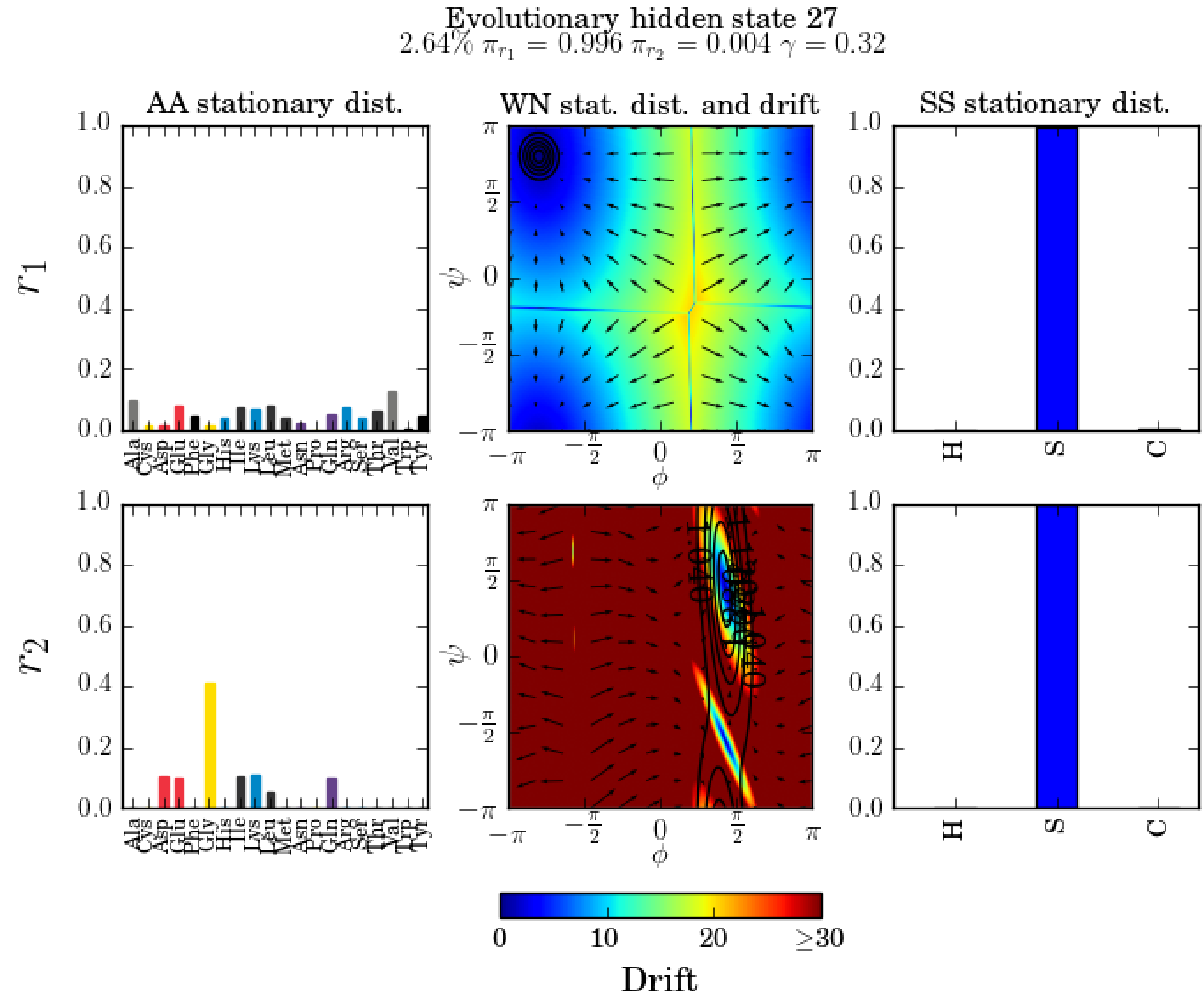

Evolutionary hidden state 27
2.64% $\pi_{r_1} = 0.996$ $\pi_{r_2} = 0.004$ $\gamma = 0.32$
AA stationary dist.
WN stat. dist. and drift
SS stationary dist.
r_1
r_2
Ala Cys Asp Glu Phe Gly His Ile Lys Leu Met Asn Pro Gln Arg Ser Thr Val Trp Tyr
H S C
ϕ
ψ
Drift
0 10 20 ≥30

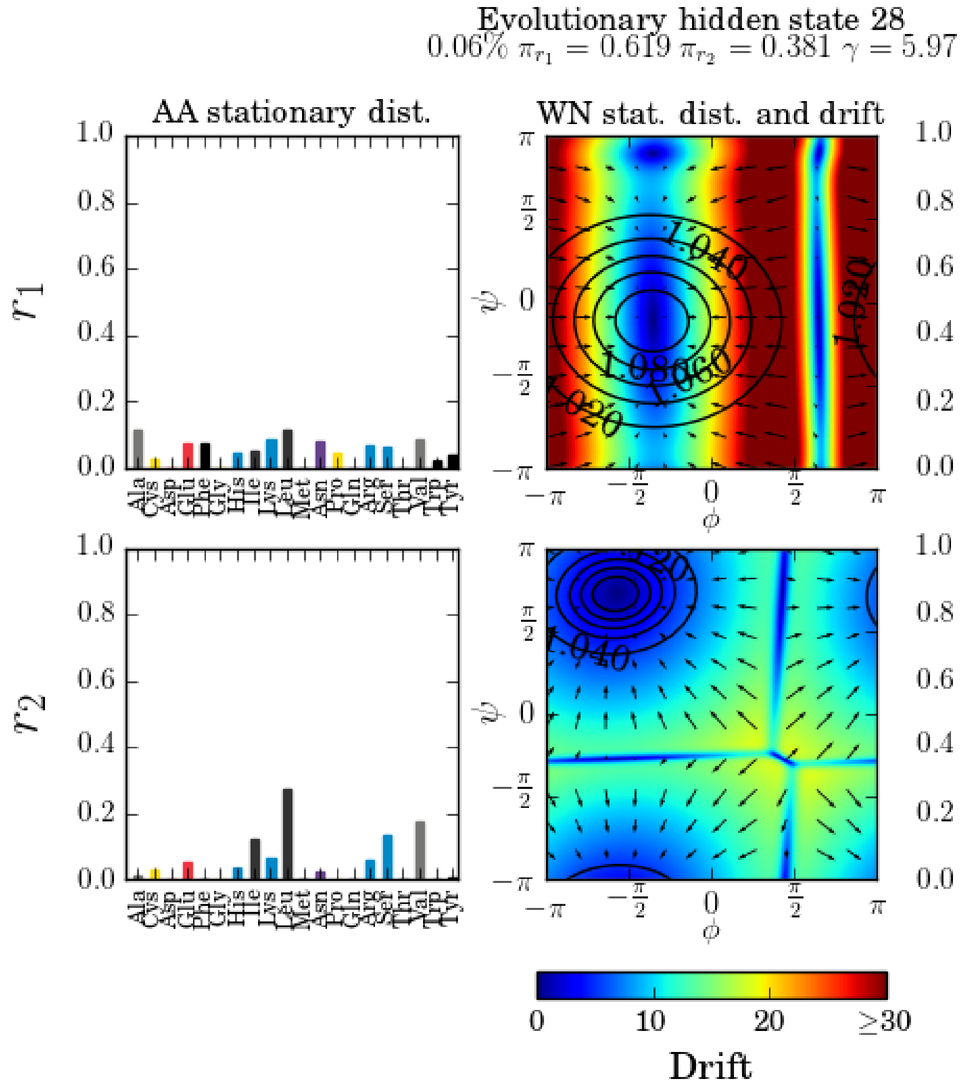

Evolutionary hidden state 28
0.06% $\pi_{r_1} = 0.619$ $\pi_{r_2} = 0.381$ $\gamma = 5.97$
AA stationary dist.
WN stat. dist. and drift
SS stationary dist.
r_1
r_2
Ala Cys Asp Glu Phe Gly His Ile Lys Leu Met Asn Pro Gln Arg Ser Thr Val Trp Tyr
ψ
ϕ
H
S
C
Drift
0
10
20
≥30

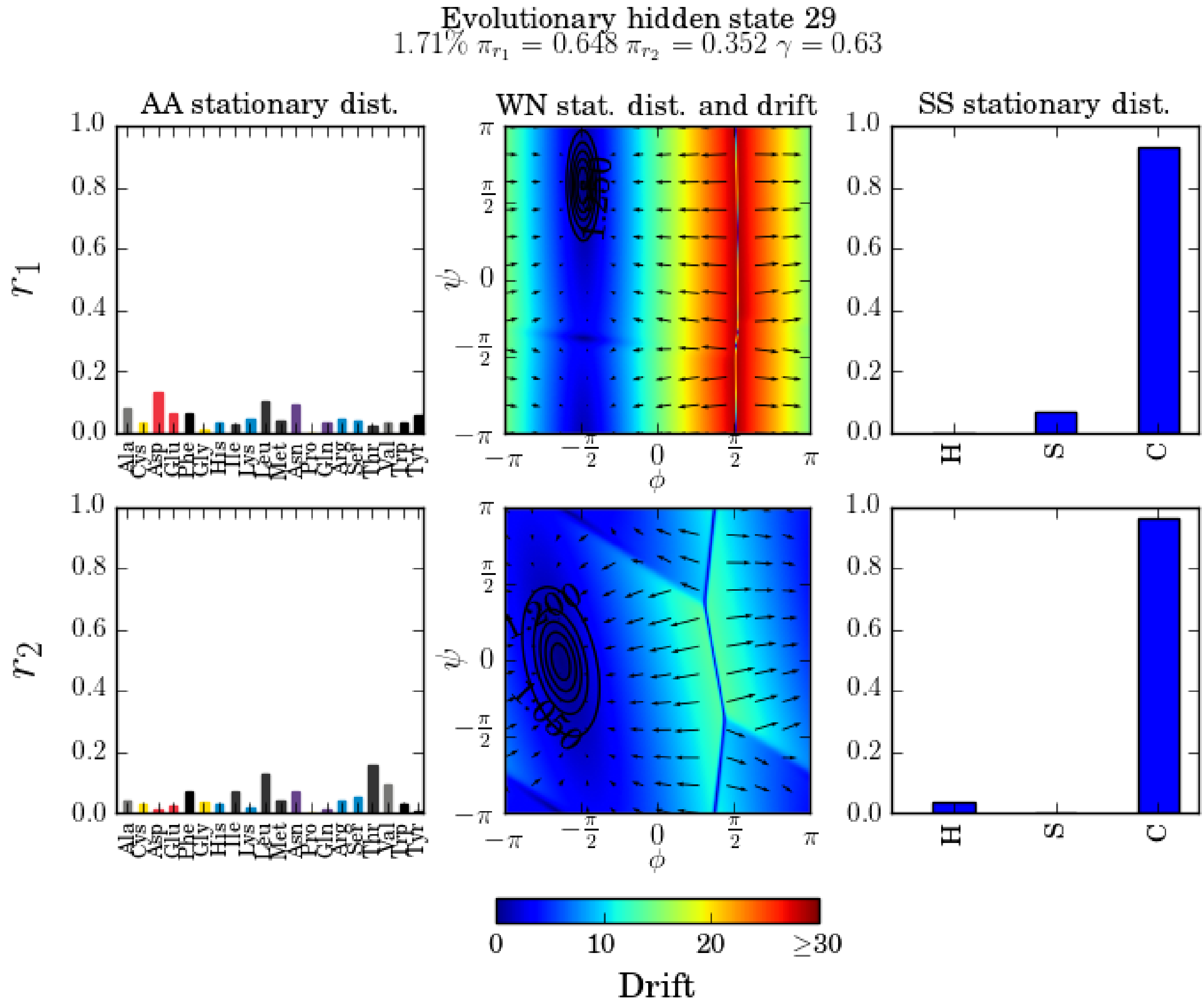

Evolutionary hidden state 29
1.71% $\pi_{r_1} = 0.648$ $\pi_{r_2} = 0.352$ $\gamma = 0.63$
AA stationary dist.
WN stat. dist. and drift
SS stationary dist.
r_1
r_2
Ala Cys Asp Glu Phe Gly His Ile Lys Leu Met Asn Pro Gln Arg Ser Thr Val Trp Tyr
H S C
ϕ
ψ
Drift
0 10 20 ≥30

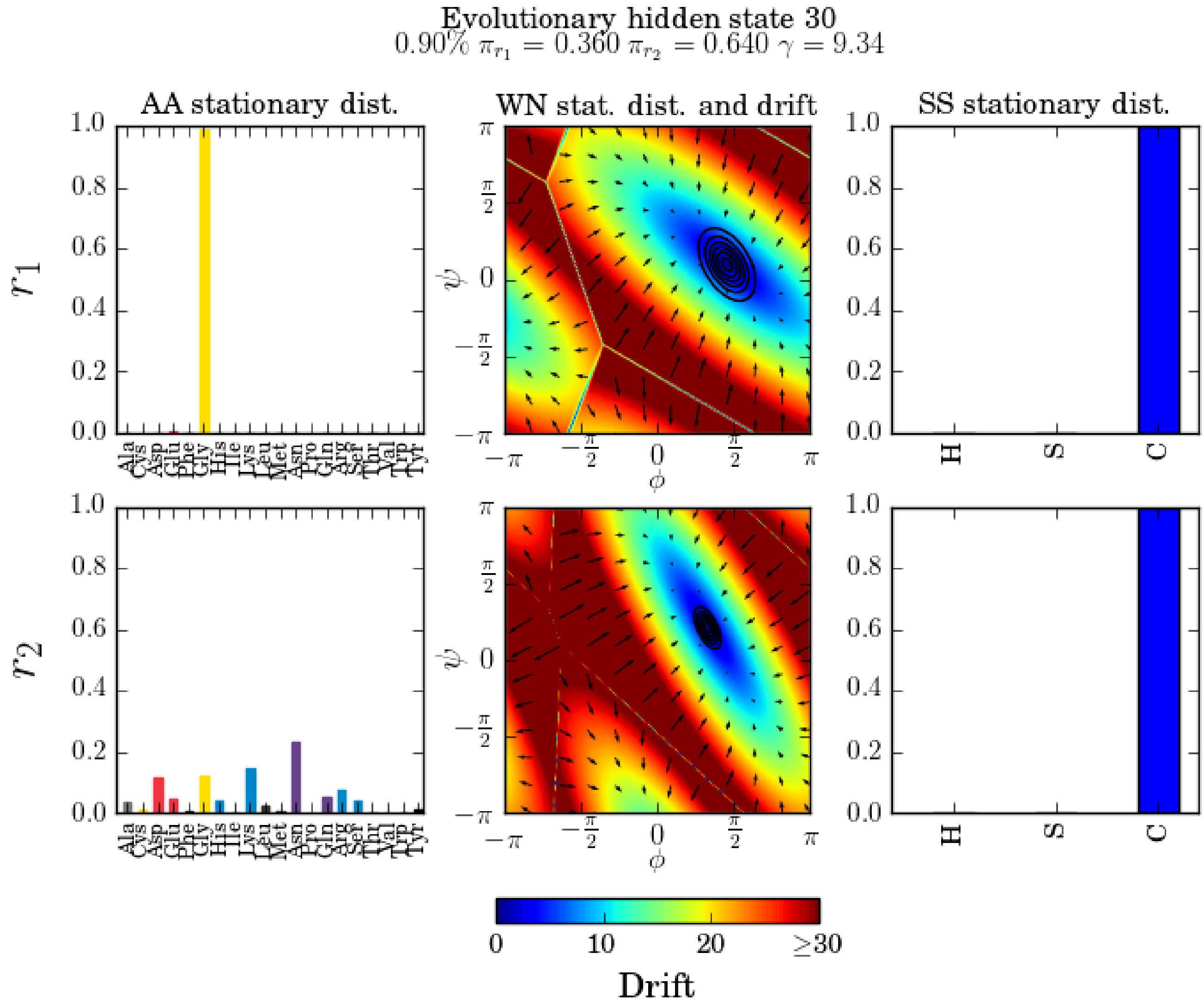
Evolutionary hidden state 30
0.90% $\pi_{r_1} = 0.360$ $\pi_{r_2} = 0.640$ $\gamma = 9.34$
AA stationary dist.
WN stat. dist. and drift
SS stationary dist.
r_1
r_2
Ala Cys Asp Glu Phe Gly His Ile Lys Leu Met Asn Pro Gln Arg Ser Thr Val Trp Tyr
H S C
ϕ
ψ
Drift
0 10 20 ≥30

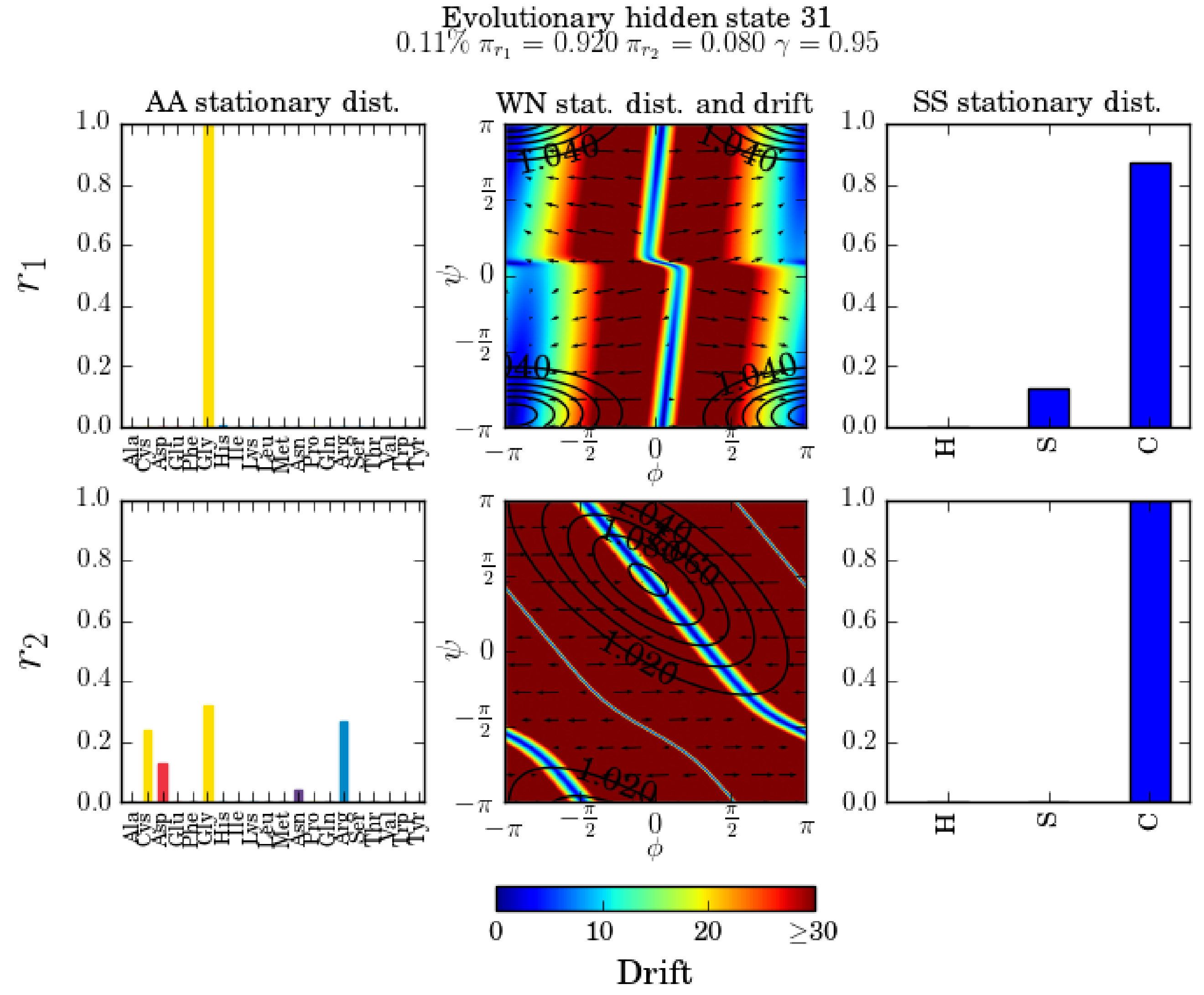

Evolutionary hidden state 31
0.11% $\pi_{r_1} = 0.920$ $\pi_{r_2} = 0.080$ $\gamma = 0.95$
AA stationary dist.
WN stat. dist. and drift
SS stationary dist.
r_1
r_2
Ala Cys Asp Glu Phe Gly His Ile Lys Leu Met Asn Pro Gln Arg Ser Thr Val Trp Tyr
H S C
ϕ
ψ
1.040
1.080
1.060
1.020
Drift
0 10 20 ≥30

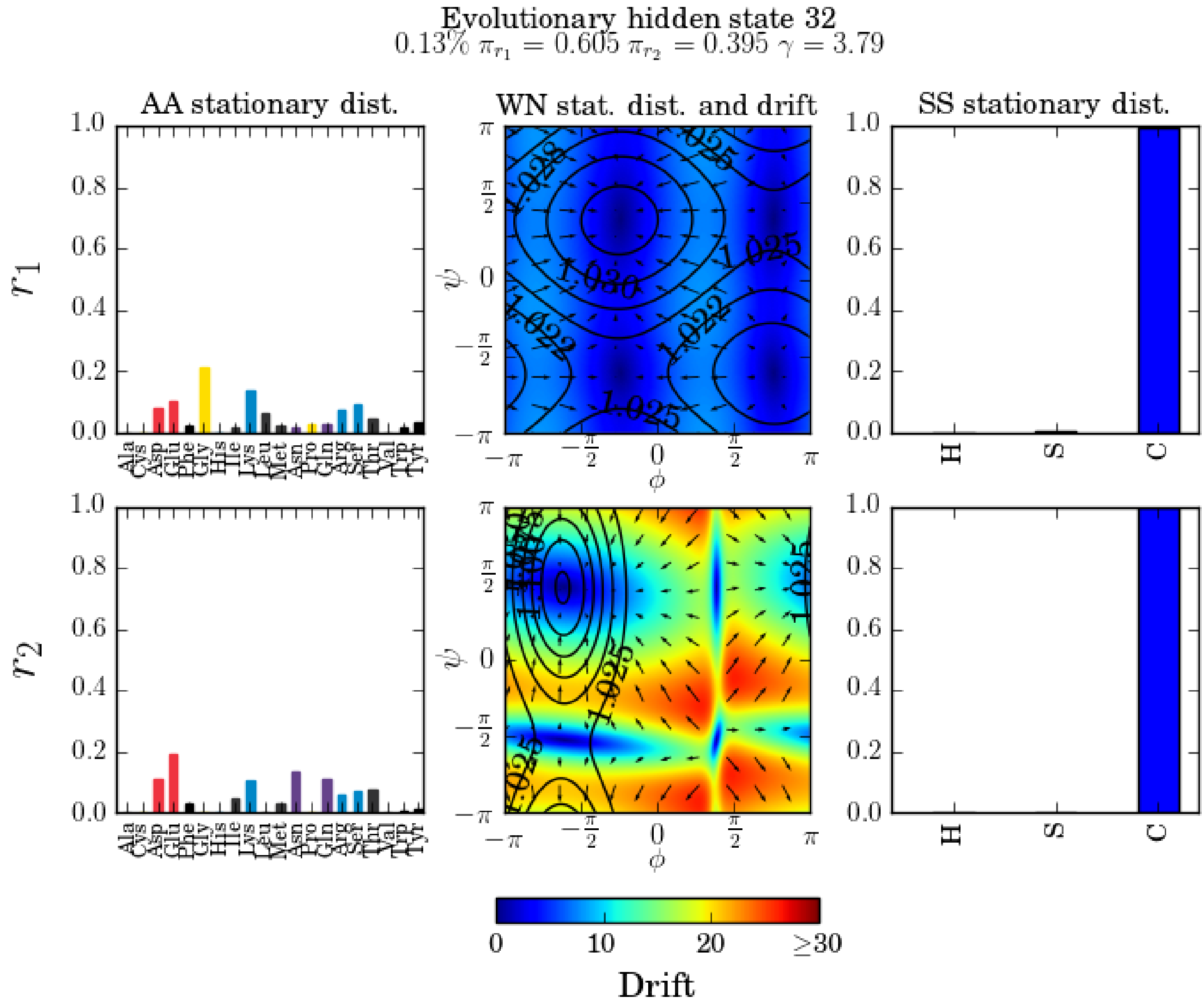
Evolutionary hidden state 32
0.13% $\pi_{r_1} = 0.605$ $\pi_{r_2} = 0.395$ $\gamma = 3.79$
AA stationary dist.
WN stat. dist. and drift
SS stationary dist.
r_1
r_2
Ala Cys Asp Glu Phe Gly His Ile Lys Leu Met Asn Pro Gln Arg Ser Thr Val Trp Tyr
H S C
ψ
ϕ
Drift
0 10 20 ≥30

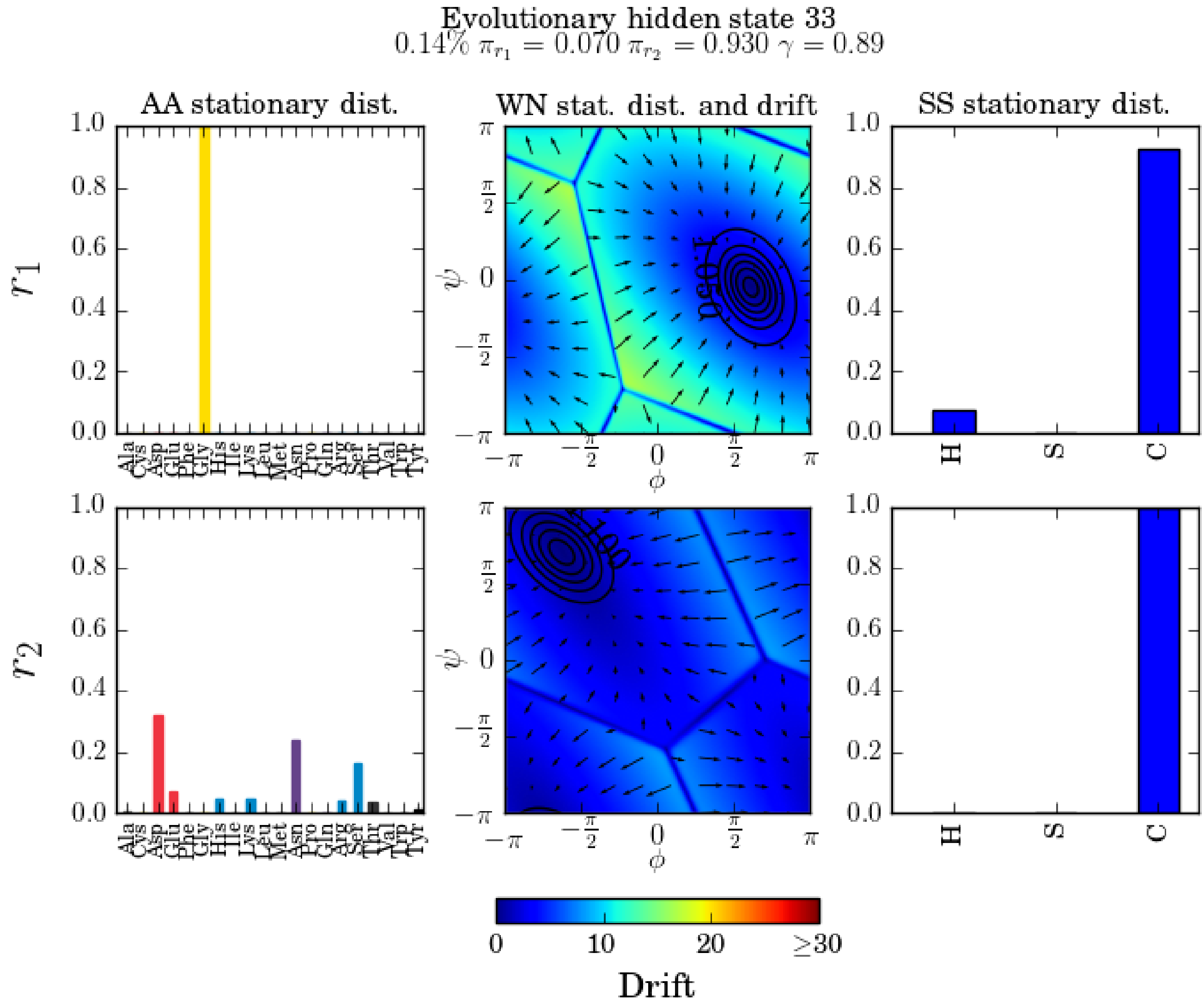

Evolutionary hidden state 33
0.14% $\pi_{r_1} = 0.070$ $\pi_{r_2} = 0.930$ $\gamma = 0.89$
AA stationary dist.
WN stat. dist. and drift
SS stationary dist.
r_1
r_2
Ala Cys Asp Glu Phe Gly His Ile Lys Leu Met Asn Pro Gln Arg Ser Thr Val Trp Tyr
ψ
ϕ
H S C
Drift
0 10 20 ≥30

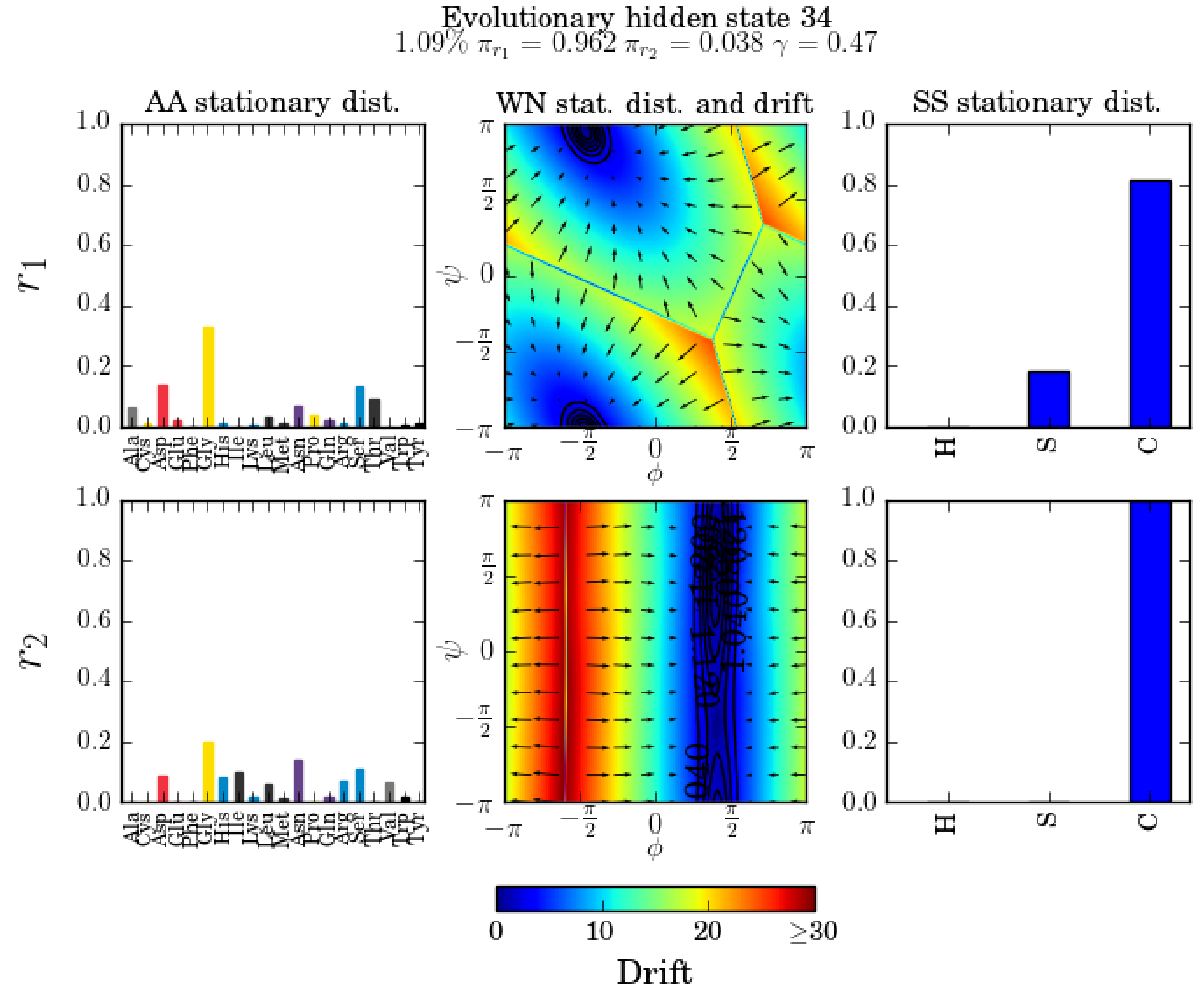

Evolutionary hidden state 34
1.09% $\pi_{r_1} = 0.962$ $\pi_{r_2} = 0.038$ $\gamma = 0.47$
AA stationary dist.
WN stat. dist. and drift
SS stationary dist.
r_1
r_2
Ala Cys Asp Glu Phe Gly His Ile Lys Leu Met Asn Pro Gln Arg Ser Thr Val Trp Tyr
ψ
ϕ
H
S
C
Drift
0
10
20
≥30

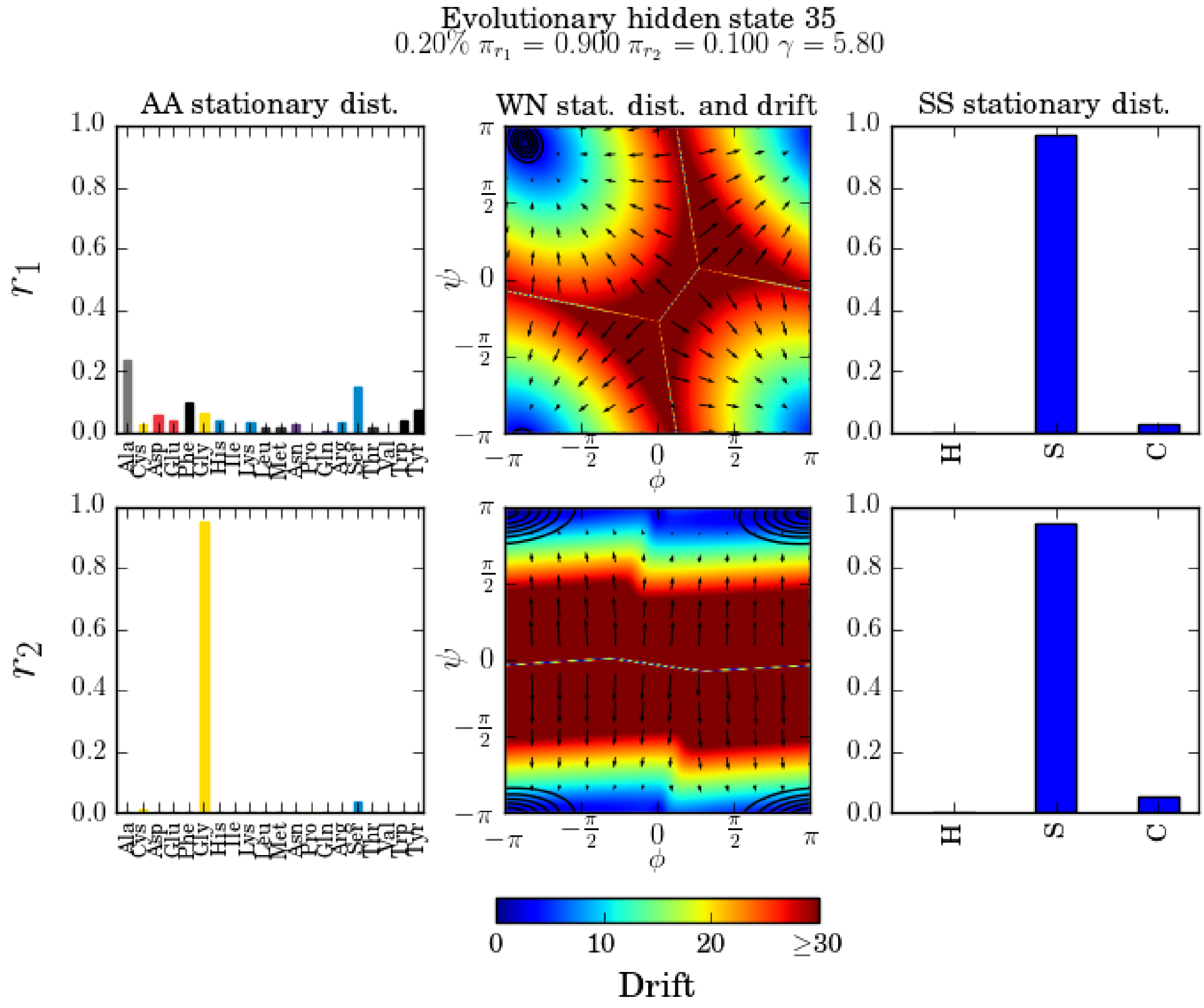

Evolutionary hidden state 35
0.20% $\pi_{r_1} = 0.900$ $\pi_{r_2} = 0.100$ $\gamma = 5.80$
AA stationary dist.
WN stat. dist. and drift
SS stationary dist.
r_1
r_2
ψ
ϕ
Ala Cys Asp Glu Phe Gly His Ile Lys Leu Met Asn Pro Gln Arg Ser Thr Val Trp Tyr
H S C
Drift
0 10 20 ≥30

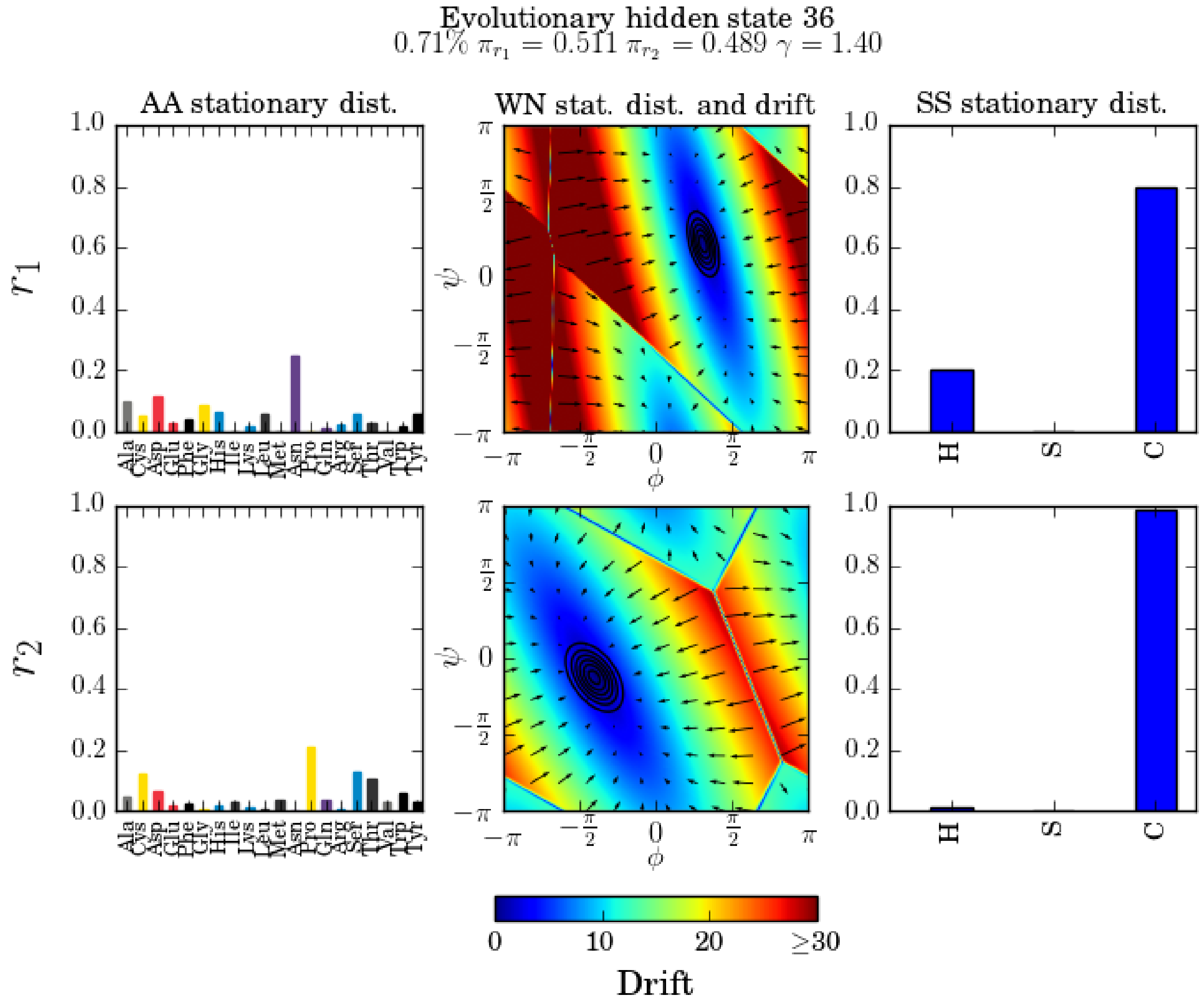

Evolutionary hidden state 36
0.71% $\pi_{r_1} = 0.511$ $\pi_{r_2} = 0.489$ $\gamma = 1.40$
AA stationary dist.
WN stat. dist. and drift
SS stationary dist.
r_1
r_2
1.0
0.8
0.6
0.4
0.2
0.0
Ala Cys Asp Glu Phe Gly His Ile Lys Leu Met Asn Pro Gln Arg Ser Thr Val Trp Tyr
ψ
ϕ
$-\pi$ $-\frac{\pi}{2}$ 0 $\frac{\pi}{2}$ π
H S C
0 10 20 ≥30
Drift

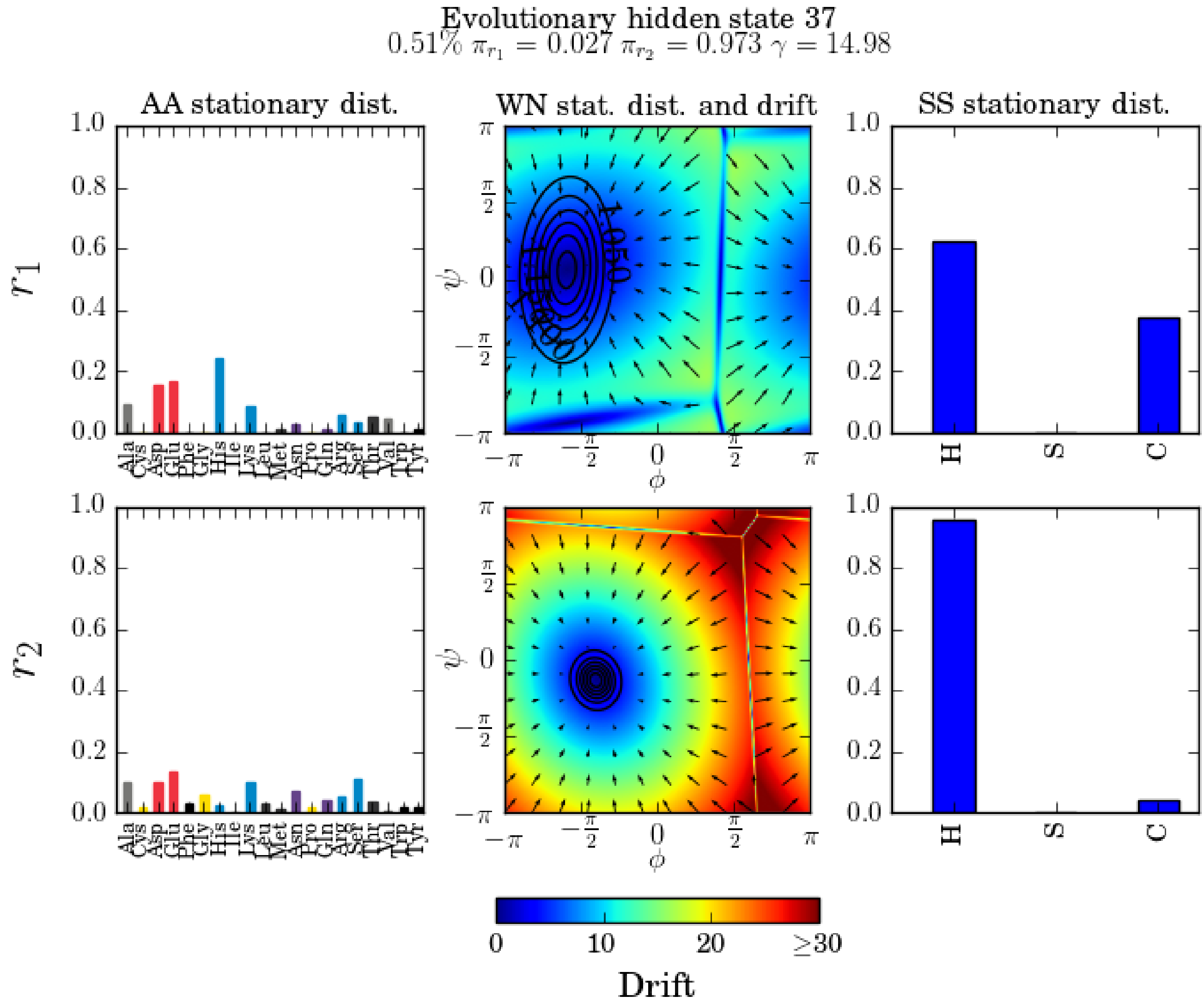

Evolutionary hidden state 37
0.51% $\pi_{r_1} = 0.027$ $\pi_{r_2} = 0.973$ $\gamma = 14.98$
AA stationary dist.
WN stat. dist. and drift
SS stationary dist.
r_1
r_2
Ala Cys Asp Glu Phe Gly His Ile Lys Leu Met Asn Pro Gln Arg Ser Thr Val Trp Tyr
H S C
ϕ
ψ
Drift
0 10 20 ≥30

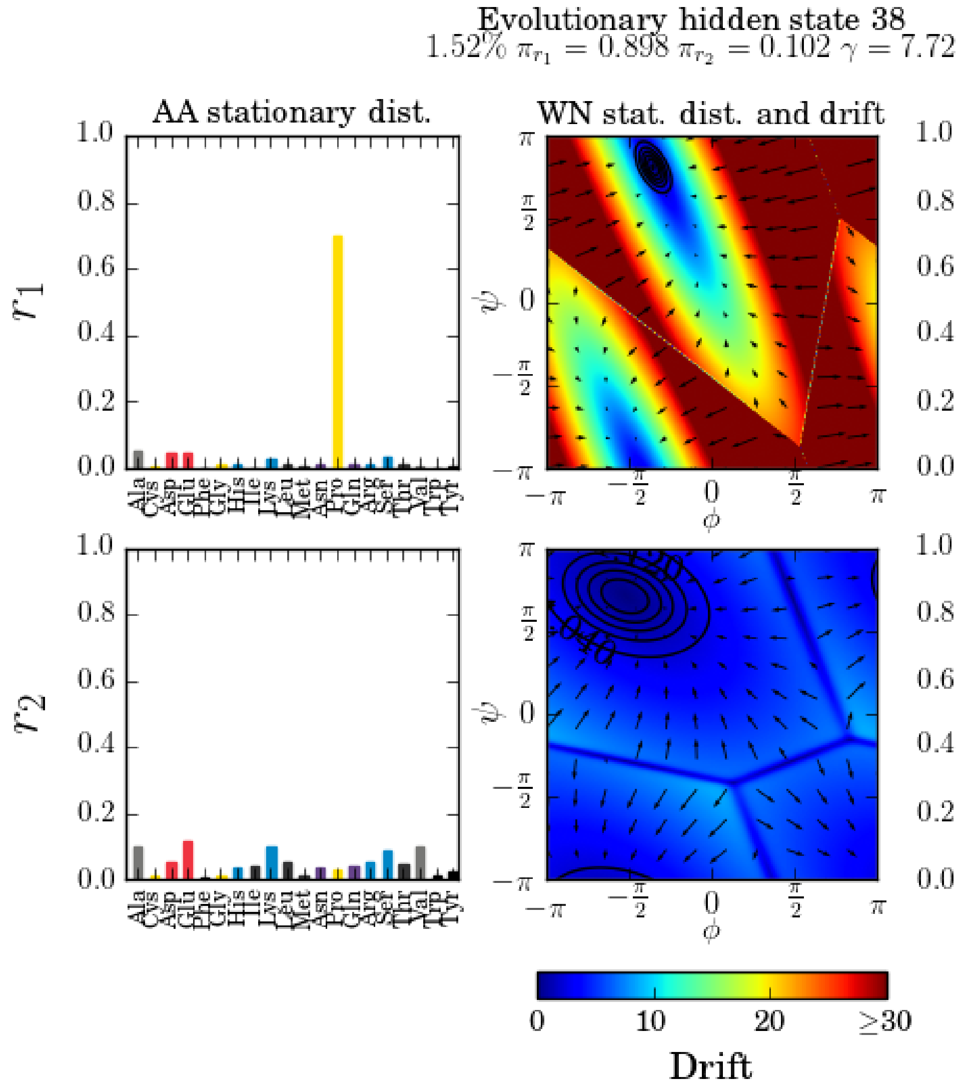

Evolutionary hidden state 38
1.52% $\pi_{r_1} = 0.898$ $\pi_{r_2} = 0.102$ $\gamma = 7.72$
AA stationary dist.
WN stat. dist. and drift
SS stationary dist.
r_1
r_2
Ala Cys Asp Glu Phe Gly His Ile Lys Leu Met Asn Pro Gln Arg Ser Thr Val Trp Tyr
H S C
ψ
ϕ
Drift
0 10 20 ≥30

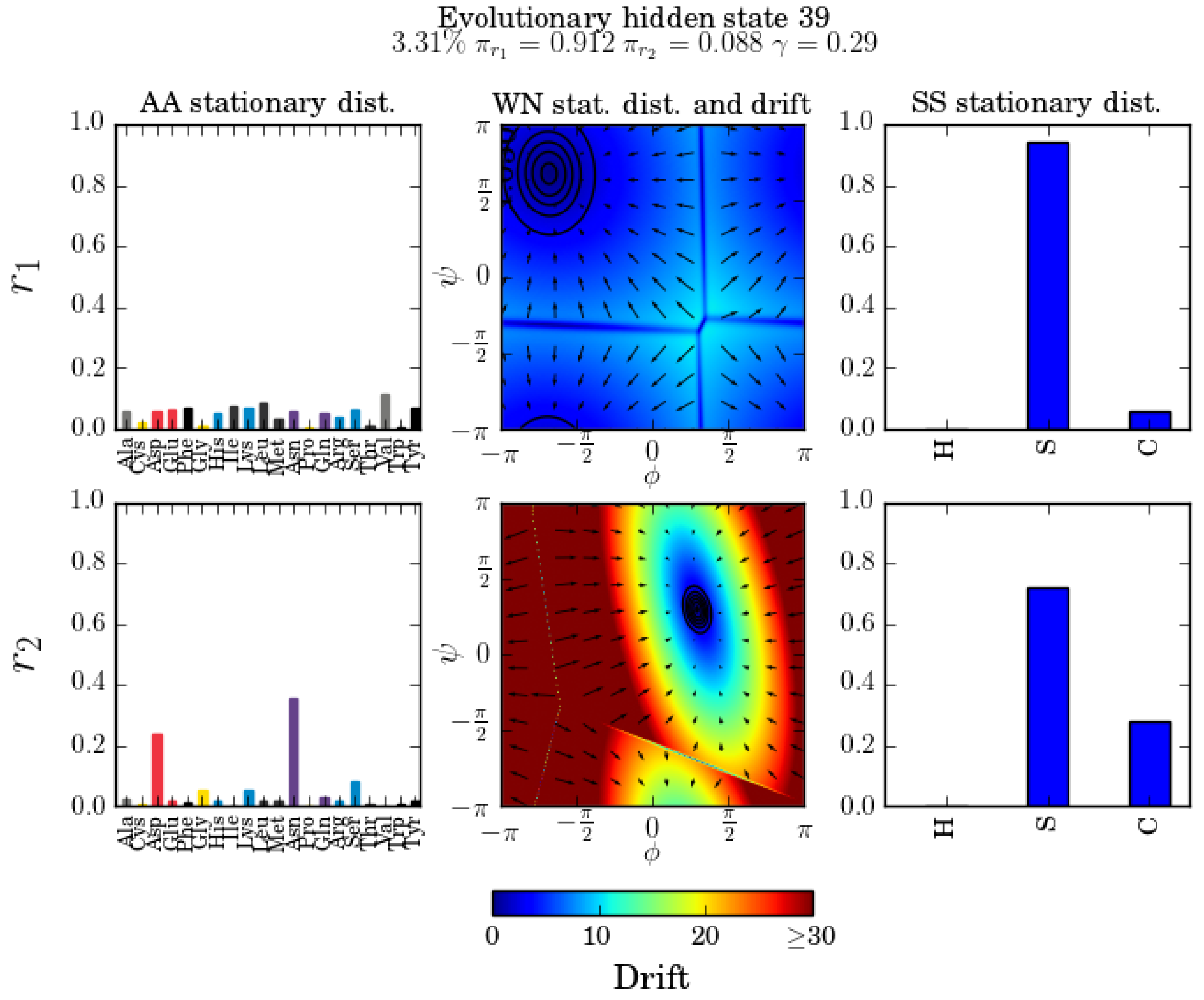
Evolutionary hidden state 39
3.31% $\pi_{r_1} = 0.912$ $\pi_{r_2} = 0.088$ $\gamma = 0.29$
AA stationary dist.
WN stat. dist. and drift
SS stationary dist.
r_1
r_2
Ala Cys Asp Glu Phe Gly His Ile Lys Leu Met Asn Pro Gln Arg Ser Thr Val Trp Tyr
ϕ
ψ
H
S
C
Drift
0
10
20
≥30

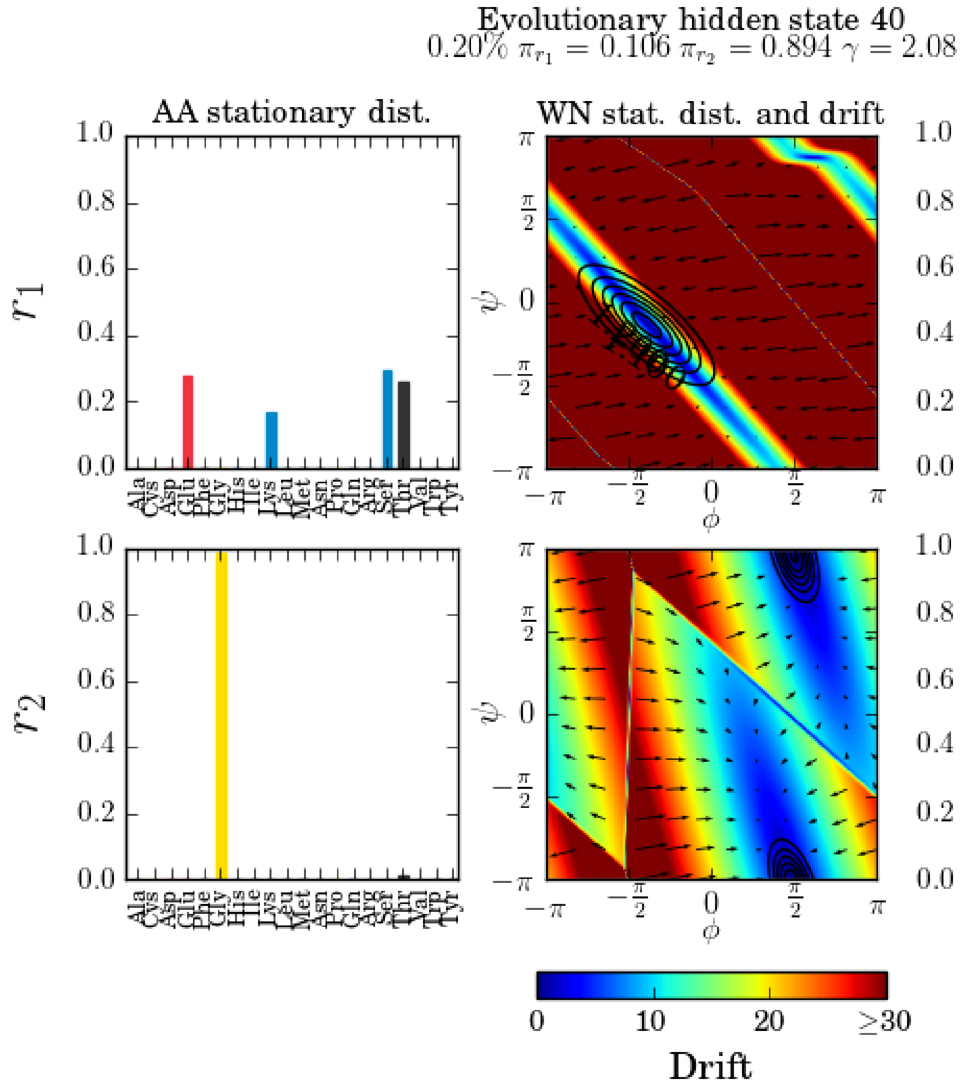

Evolutionary hidden state 40
0.20% $\pi_{r_1} = 0.106$ $\pi_{r_2} = 0.894$ $\gamma = 2.08$
AA stationary dist.
WN stat. dist. and drift
SS stationary dist.
r_1
r_2
Ala Cys Asp Glu Phe Gly His Ile Lys Leu Met Asn Pro Gln Arg Ser Thr Val Trp Tyr
H S C
ϕ
ψ
Drift
0 10 20 ≥30

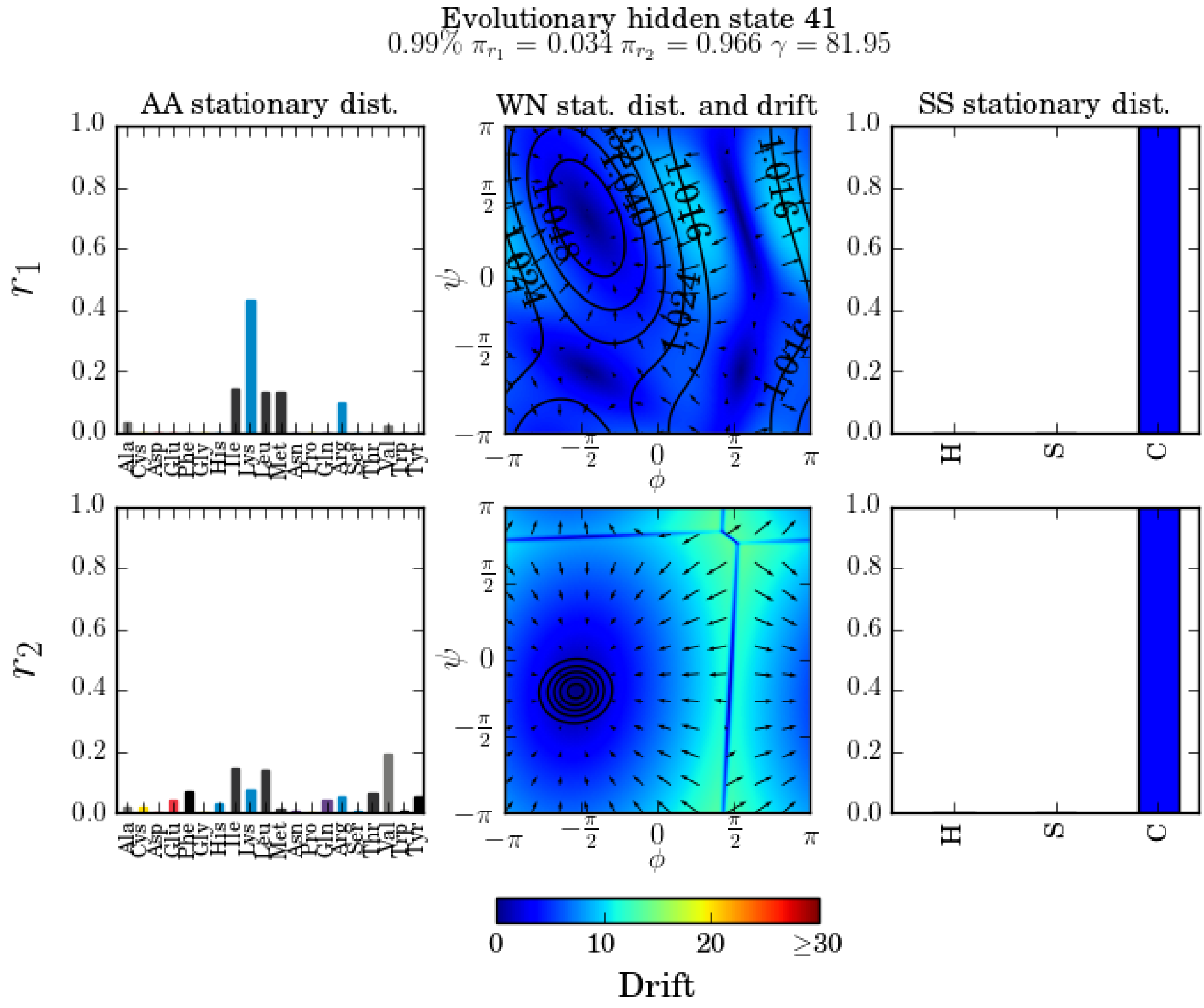

Evolutionary hidden state 41
0.99% $\pi_{r_1} = 0.034$ $\pi_{r_2} = 0.966$ $\gamma = 81.95$
AA stationary dist.
WN stat. dist. and drift
SS stationary dist.
r_1
r_2
Ala Cys Asp Glu Phe Gly His Ile Lys Leu Met Asn Pro Gln Arg Ser Thr Val Trp Tyr
H S C
ϕ
ψ
1.048
1.040
1.032
1.024
1.016
Drift
0 10 20 ≥30

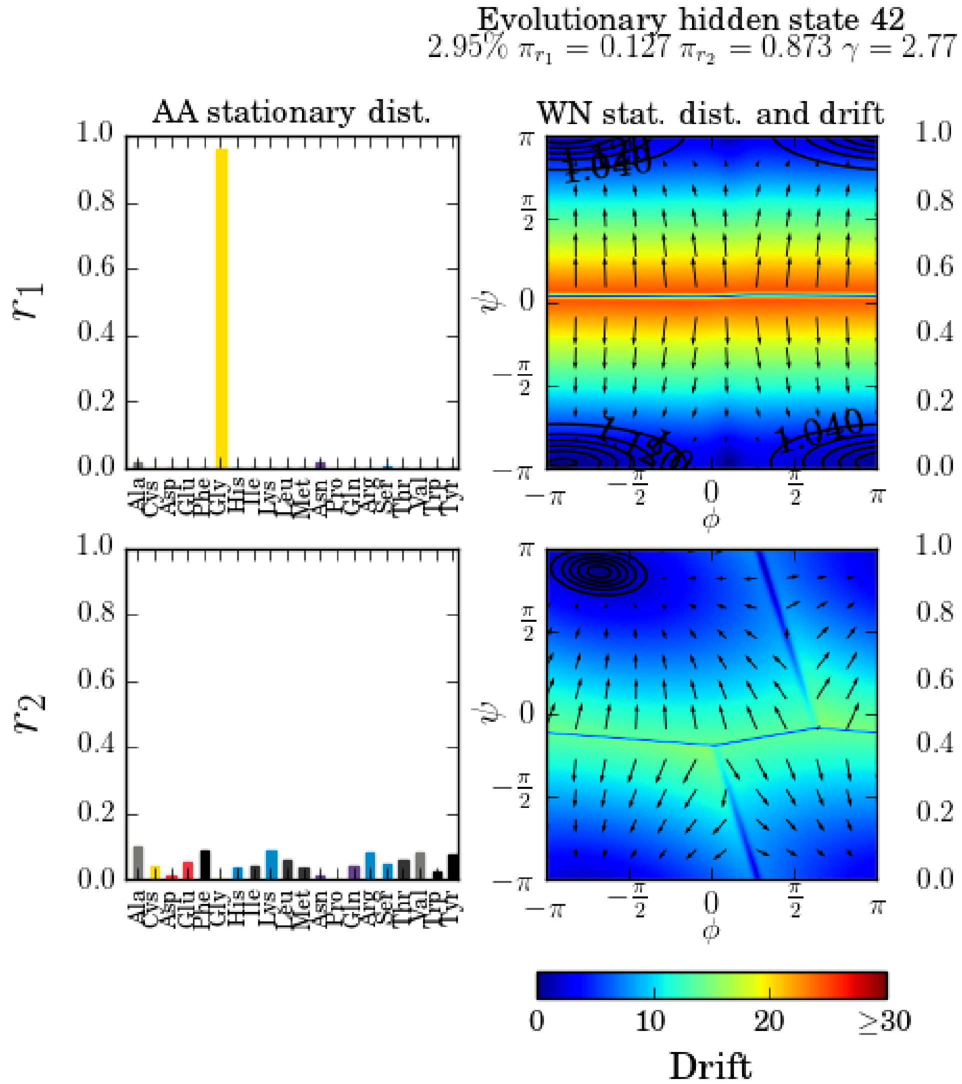

Evolutionary hidden state 42
2.95% $\pi_{r_1} = 0.127$ $\pi_{r_2} = 0.873$ $\gamma = 2.77$
AA stationary dist.
WN stat. dist. and drift
SS stationary dist.
r_1
r_2
Ala Cys Asp Glu Phe Gly His Ile Lys Leu Met Asn Pro Gln Arg Ser Thr Val Trp Tyr
ψ
ϕ
H S C
Drift
0 10 20 ≥30

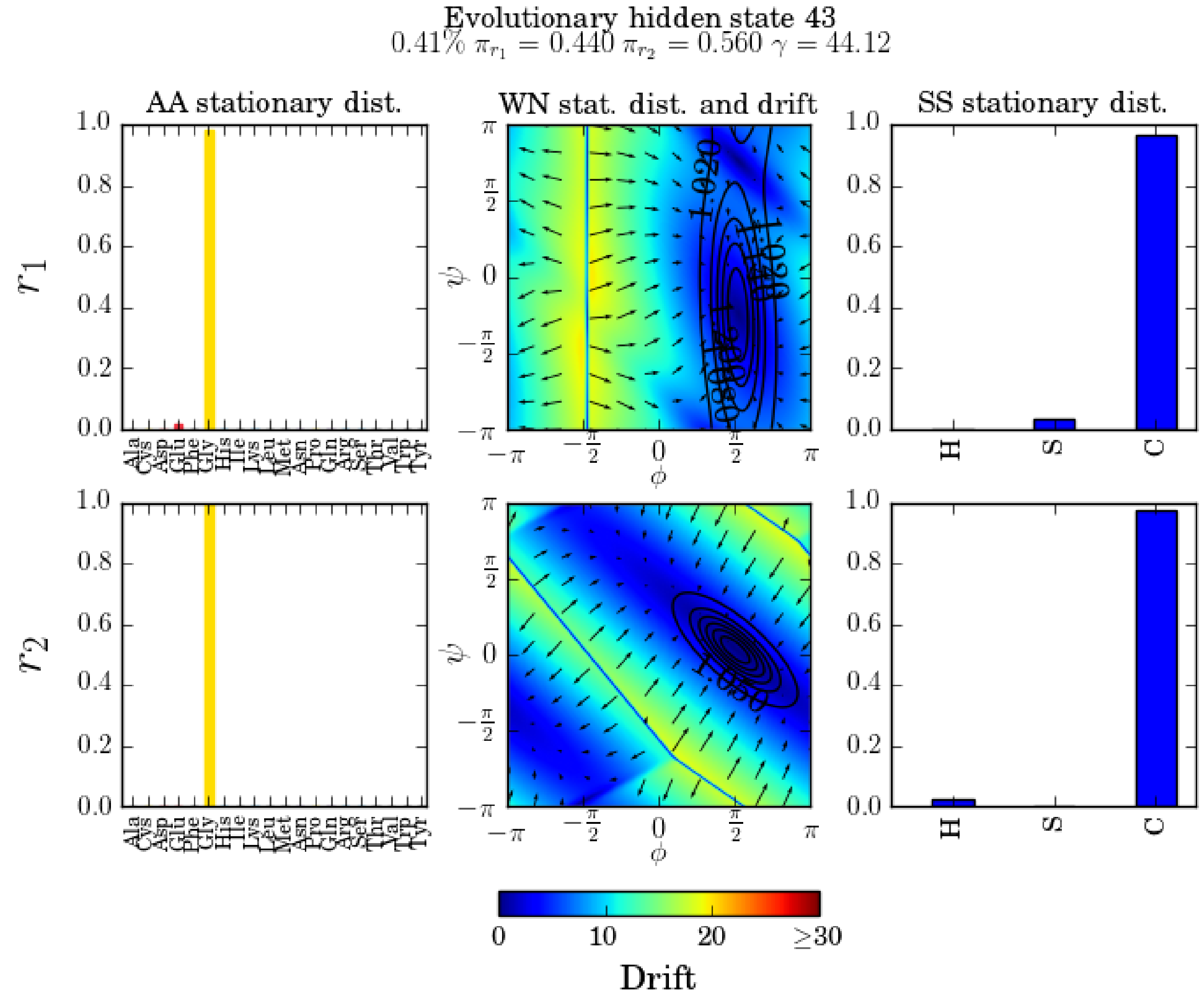
Evolutionary hidden state 43
0.41% $\pi_{r_1} = 0.440$ $\pi_{r_2} = 0.560$ $\gamma = 44.12$
AA stationary dist.
WN stat. dist. and drift
SS stationary dist.
r_1
r_2
ψ
ϕ
Ala Cys Asp Glu Phe Gly His Ile Lys Leu Met Asn Pro Gln Arg Ser Thr Val Trp Tyr
H S C
Drift
0 10 20 ≥30

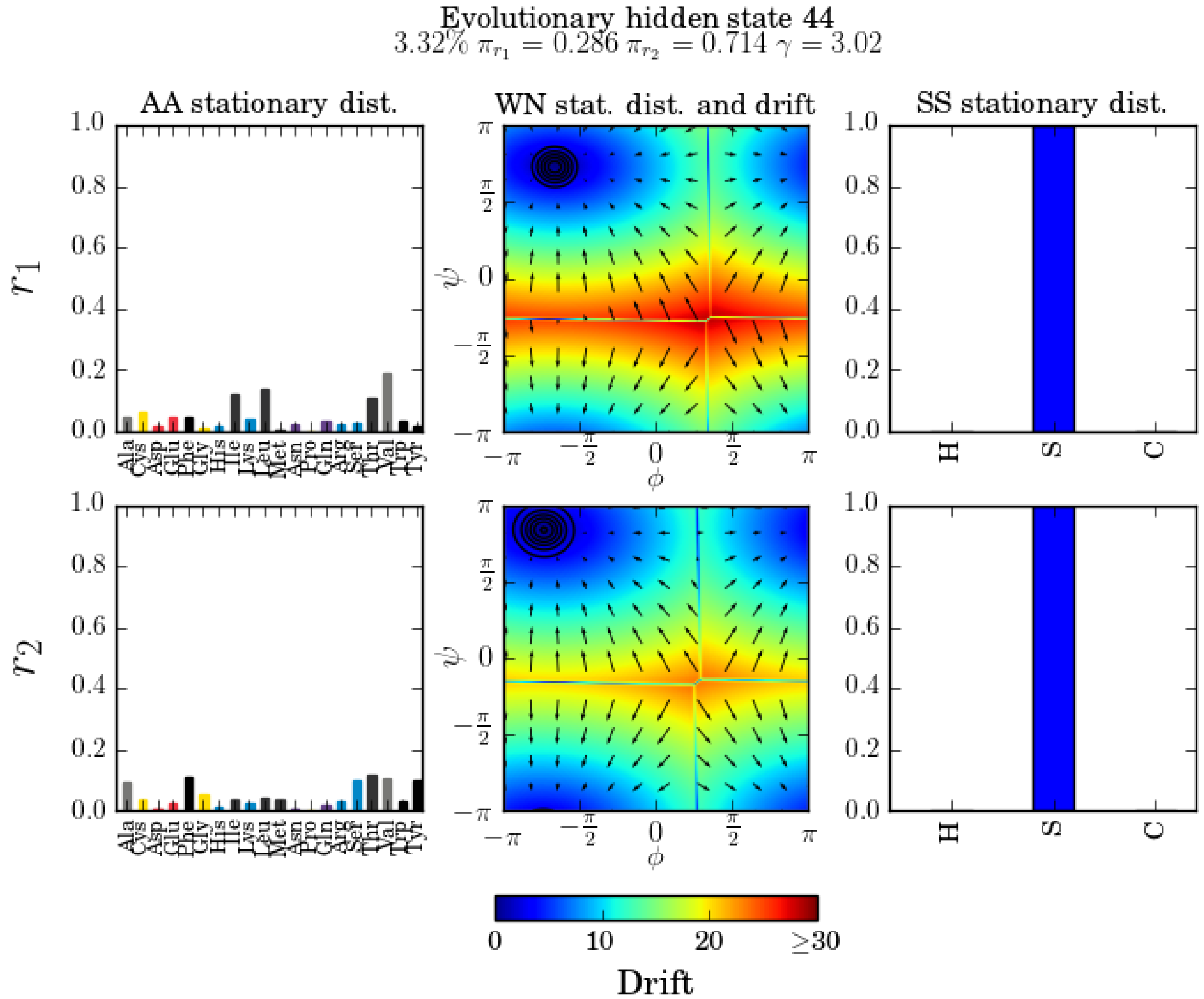

Evolutionary hidden state 44
3.32% $\pi_{r_1} = 0.286$ $\pi_{r_2} = 0.714$ $\gamma = 3.02$
AA stationary dist.
WN stat. dist. and drift
SS stationary dist.
r_1
r_2
Ala Cys Asp Glu Phe Gly His Ile Lys Leu Met Asn Pro Gln Arg Ser Thr Val Trp Tyr
ϕ
ψ
H
S
C
Drift
0
10
20
≥30

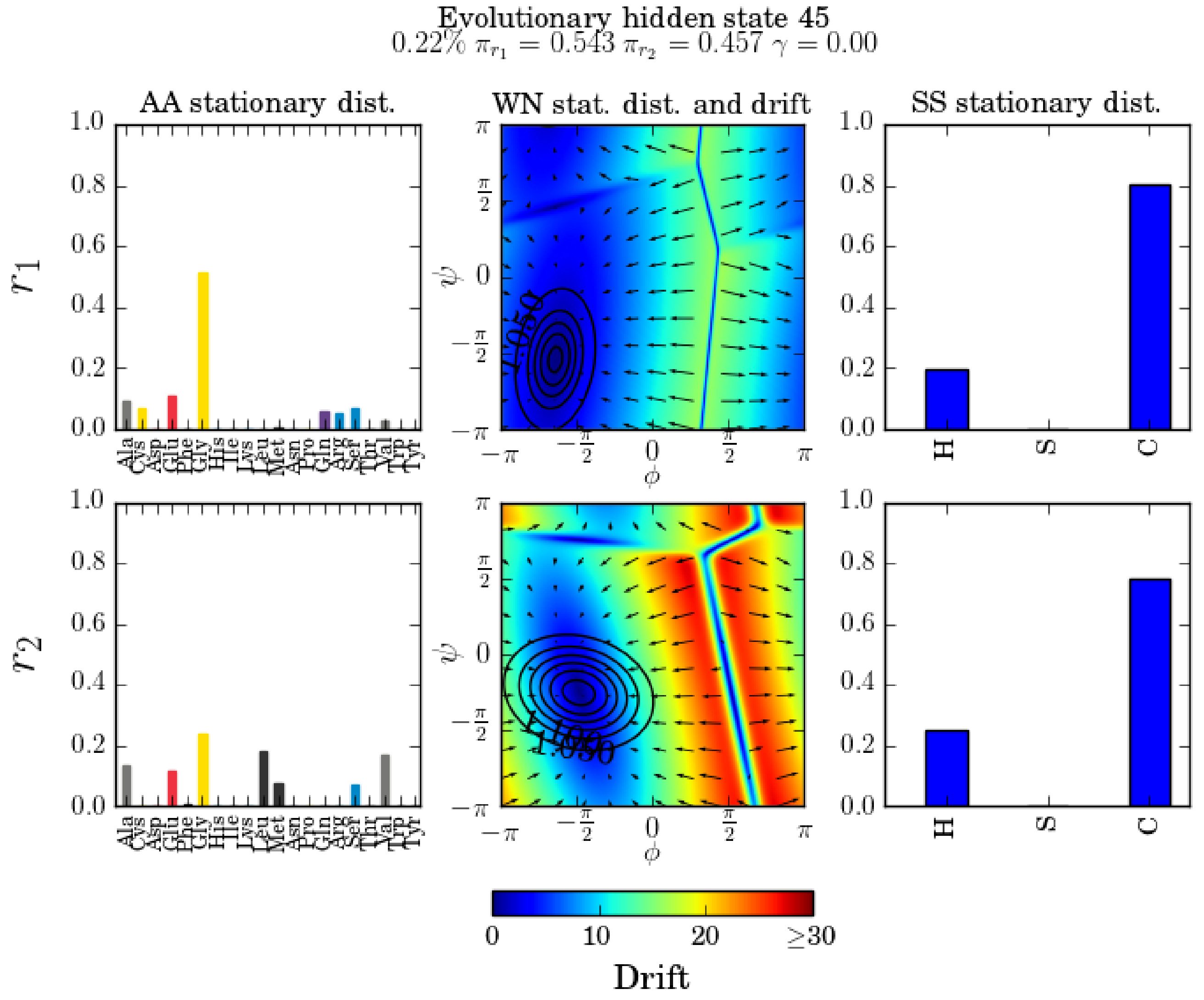

Evolutionary hidden state 45
0.22% $\pi_{r_1} = 0.543$ $\pi_{r_2} = 0.457$ $\gamma = 0.00$
AA stationary dist.
WN stat. dist. and drift
SS stationary dist.
r_1
r_2
Ala Cys Asp Glu Phe Gly His Ile Lys Leu Met Asn Pro Gln Arg Ser Thr Val Trp Tyr
H S C
ψ
ϕ
1.050
Drift
0 10 20 ≥30

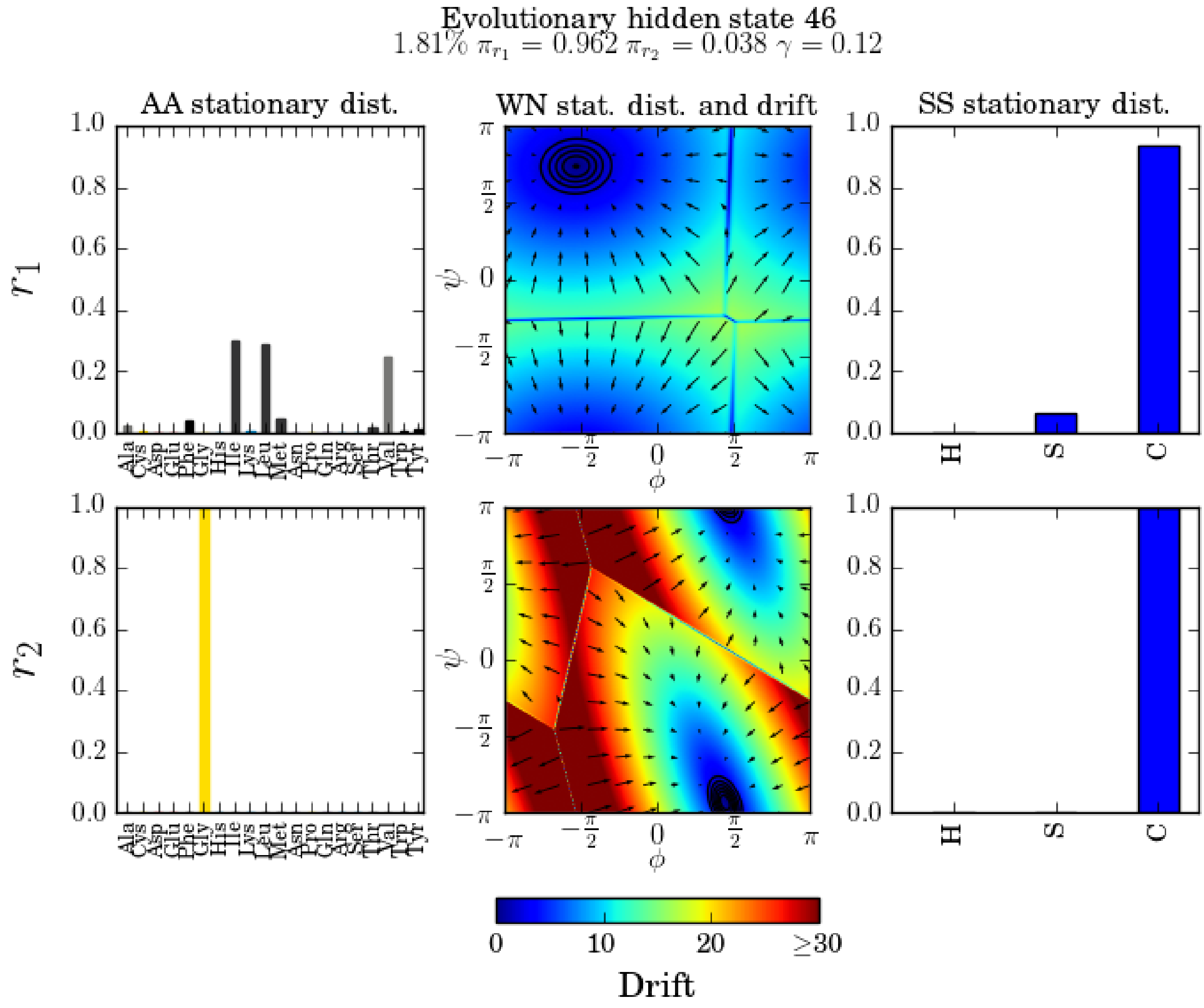

Evolutionary hidden state 46
$1.81\%\ \pi_{r_1} = 0.962\ \pi_{r_2} = 0.038\ \gamma = 0.12$
AA stationary dist.
WN stat. dist. and drift
SS stationary dist.
r_1
r_2
1.0
0.8
0.6
0.4
0.2
0.0
Ala
Cys
Asp
Glu
Phe
Gly
His
Ile
Lys
Leu
Met
Asn
Pro
Gln
Arg
Ser
Thr
Val
Trp
Tyr
ψ
ϕ
π
$\frac{\pi}{2}$
0
$-\frac{\pi}{2}$
$-\pi$
H
S
C
0
10
20
≥30
Drift

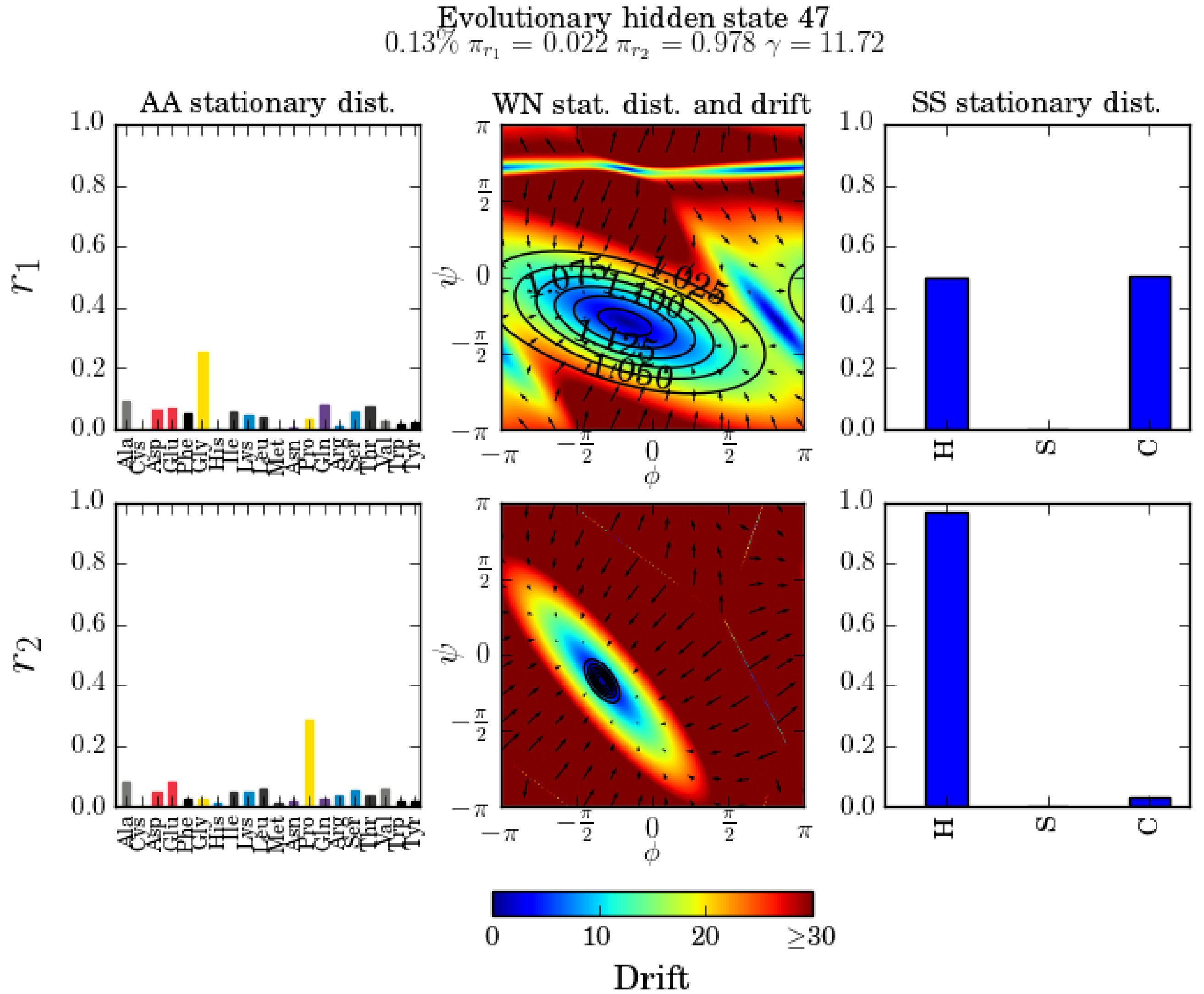

Evolutionary hidden state 47
0.13% $\pi_{r_1} = 0.022$ $\pi_{r_2} = 0.978$ $\gamma = 11.72$
AA stationary dist.
WN stat. dist. and drift
SS stationary dist.
r_1
r_2
1.0
0.8
0.6
0.4
0.2
0.0
Ala Cys Asp Glu Phe Gly His Ile Lys Leu Met Asn Pro Gln Arg Ser Thr Val Trp Tyr
π
$\frac{\pi}{2}$
0
$-\frac{\pi}{2}$
$-\pi$
ψ
ϕ
1.025
1.050
1.075
1.100
1.125
H
S
C
0
10
20
≥30
Drift

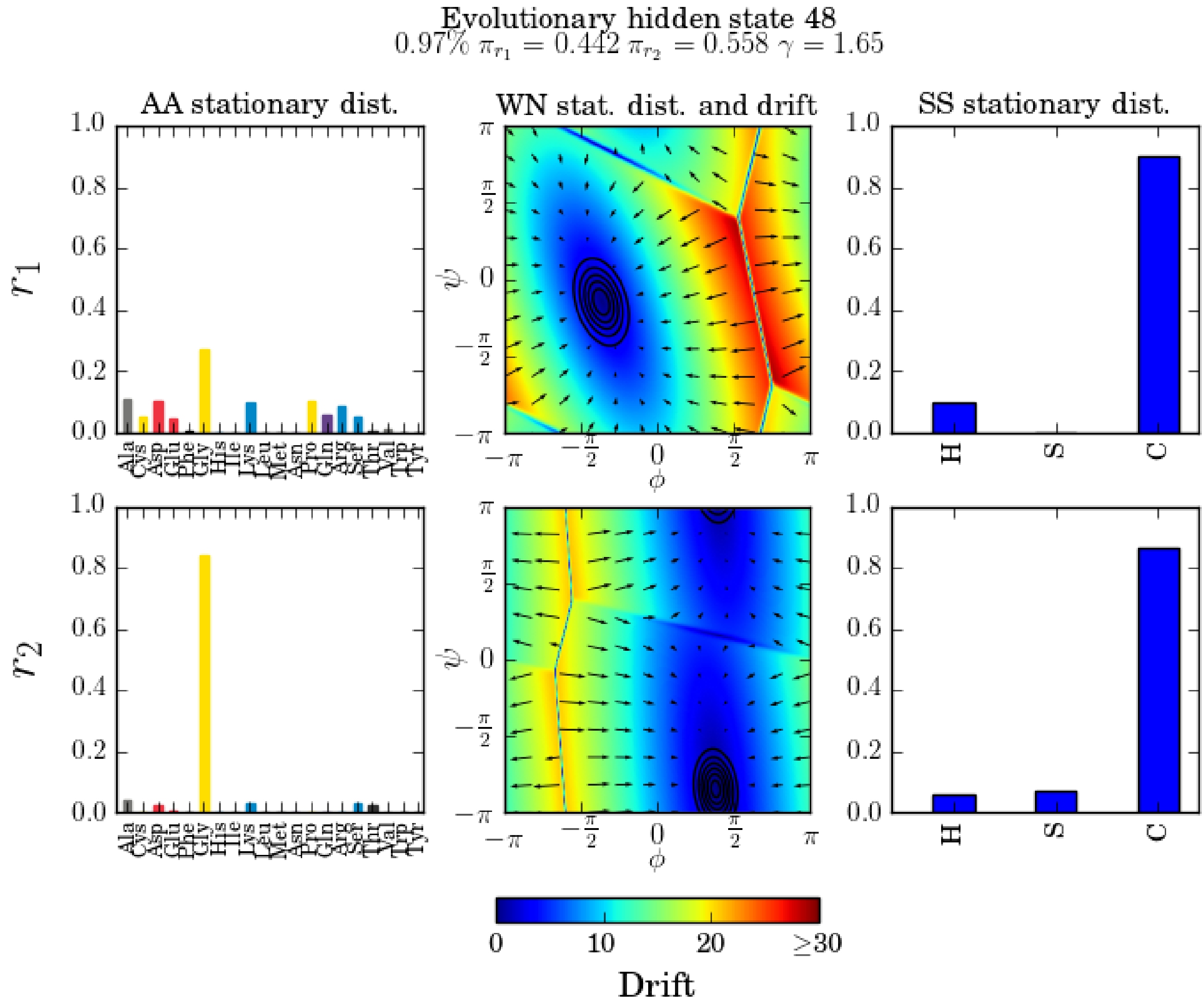

Evolutionary hidden state 48
0.97% $\pi_{r_1} = 0.442$ $\pi_{r_2} = 0.558$ $\gamma = 1.65$
AA stationary dist.
WN stat. dist. and drift
SS stationary dist.
r_1
r_2
Ala Cys Asp Glu Phe Gly His Ile Lys Leu Met Asn Pro Gln Arg Ser Thr Val Trp Tyr
ϕ
ψ
H
S
C
Drift
0
10
20
≥30

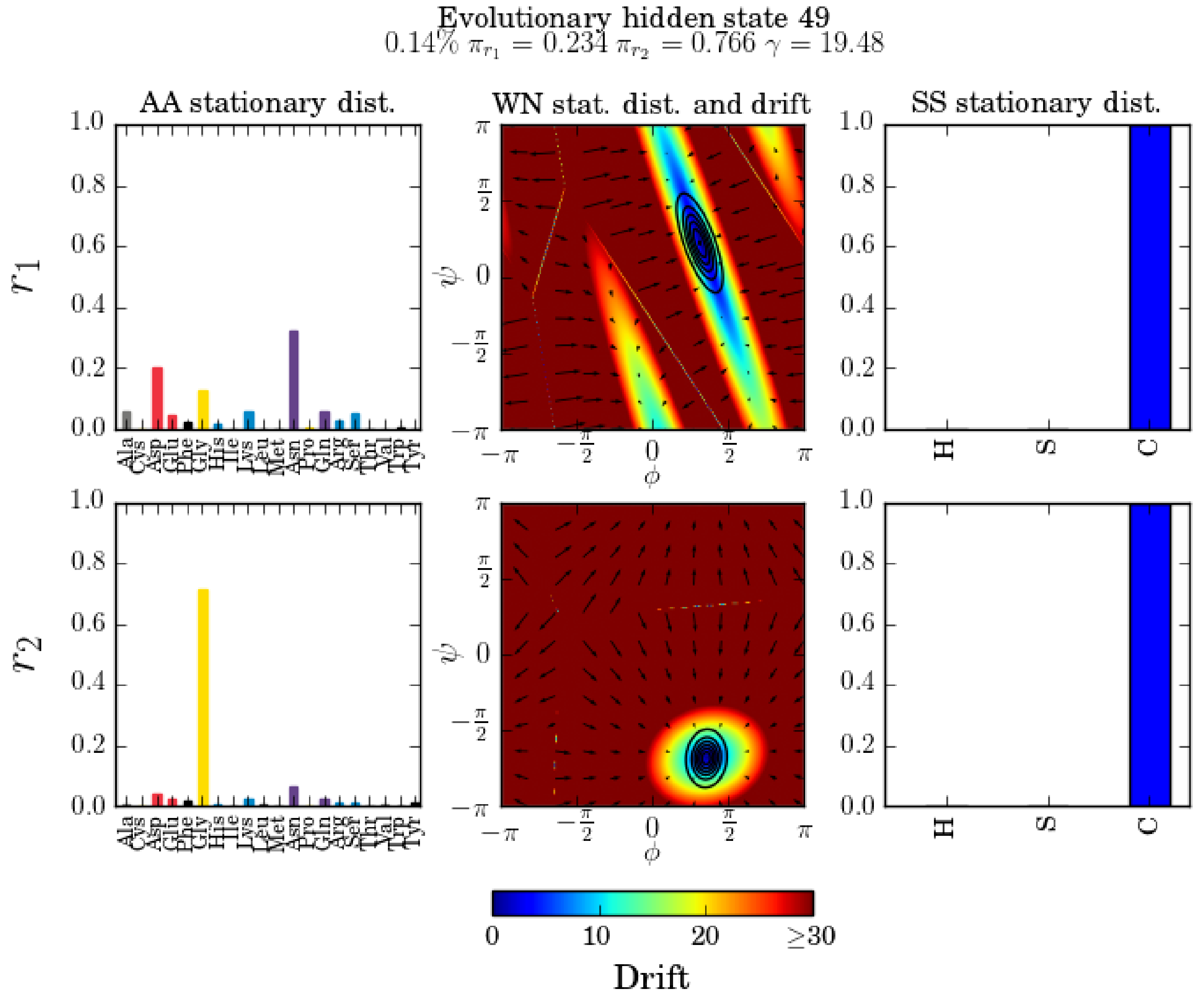

Evolutionary hidden state 49
0.14% $\pi_{r_1} = 0.234$ $\pi_{r_2} = 0.766$ $\gamma = 19.48$
AA stationary dist.
WN stat. dist. and drift
SS stationary dist.
r_1
r_2
Ala Cys Asp Glu Phe Gly His Ile Lys Leu Met Asn Pro Gln Arg Ser Thr Val Trp Tyr
ψ
ϕ
H S C
Drift
0 10 20 ≥30

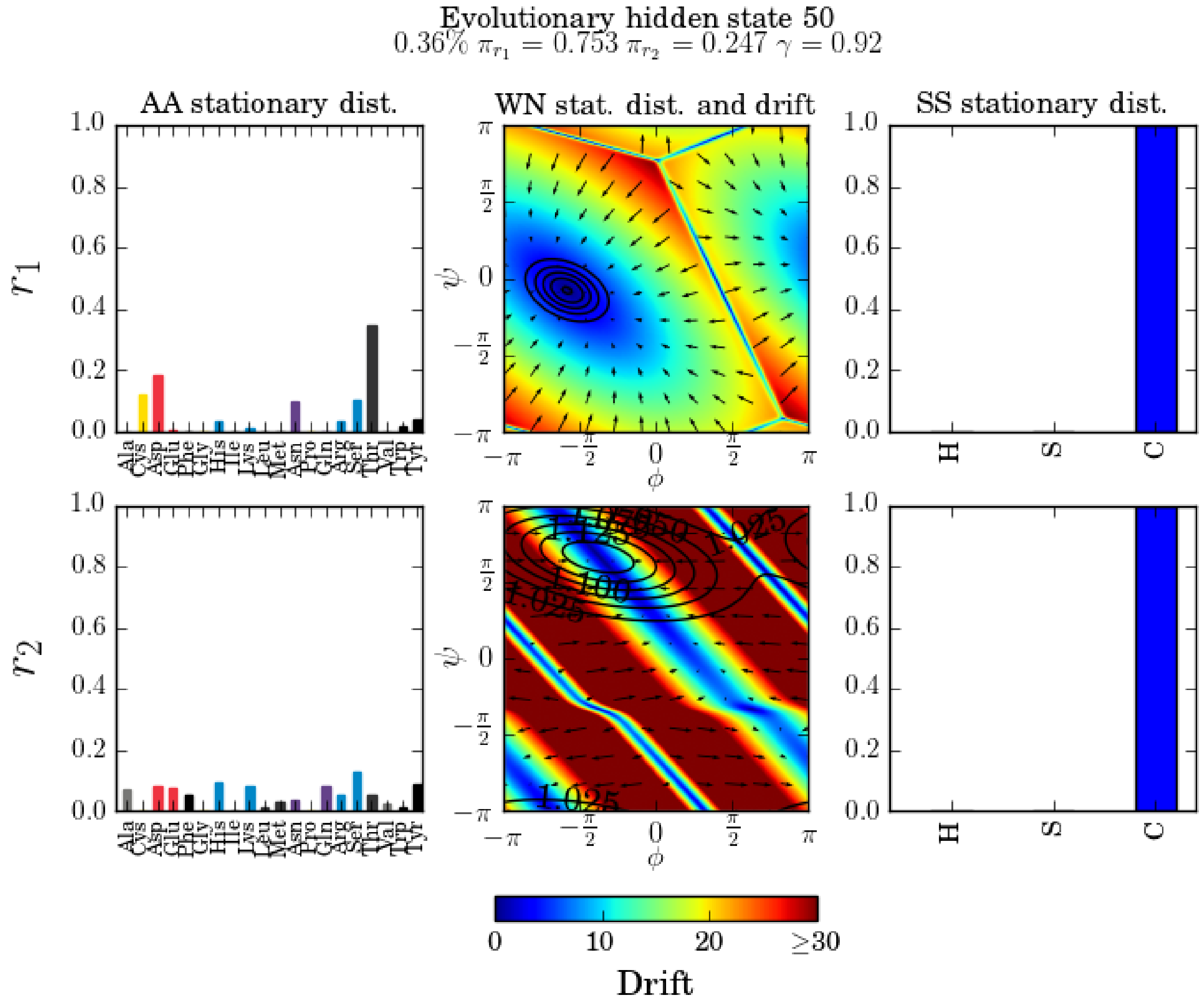
Evolutionary hidden state 50
0.36% $\pi_{r_1} = 0.753$ $\pi_{r_2} = 0.247$ $\gamma = 0.92$
AA stationary dist.
WN stat. dist. and drift
SS stationary dist.
r_1
r_2
ψ
ϕ
Ala
Cys
Asp
Glu
Phe
Gly
His
Ile
Lys
Leu
Met
Asn
Pro
Gln
Arg
Ser
Thr
Val
Trp
Tyr
H
S
C
1.100
1.025
Drift
0
10
20
≥30

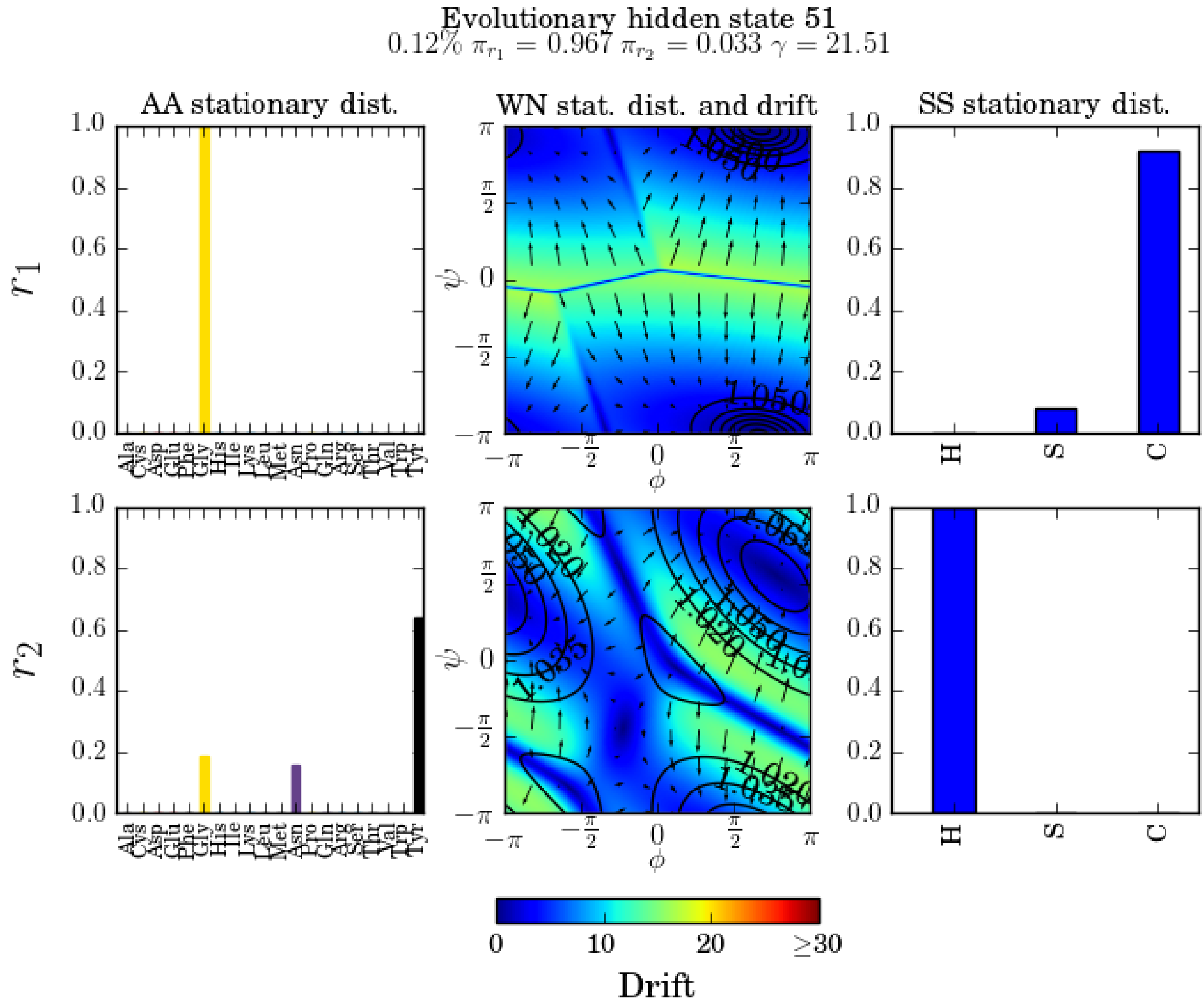
Evolutionary hidden state 51
0.12% $\pi_{r_1} = 0.967$ $\pi_{r_2} = 0.033$ $\gamma = 21.51$
AA stationary dist.
WN stat. dist. and drift
SS stationary dist.
r_1
r_2
Ala Cys Asp Glu Phe Gly His Ile Lys Leu Met Asn Pro Gln Arg Ser Thr Val Trp Tyr
H S C
ϕ
ψ
Drift
0 10 20 ≥30

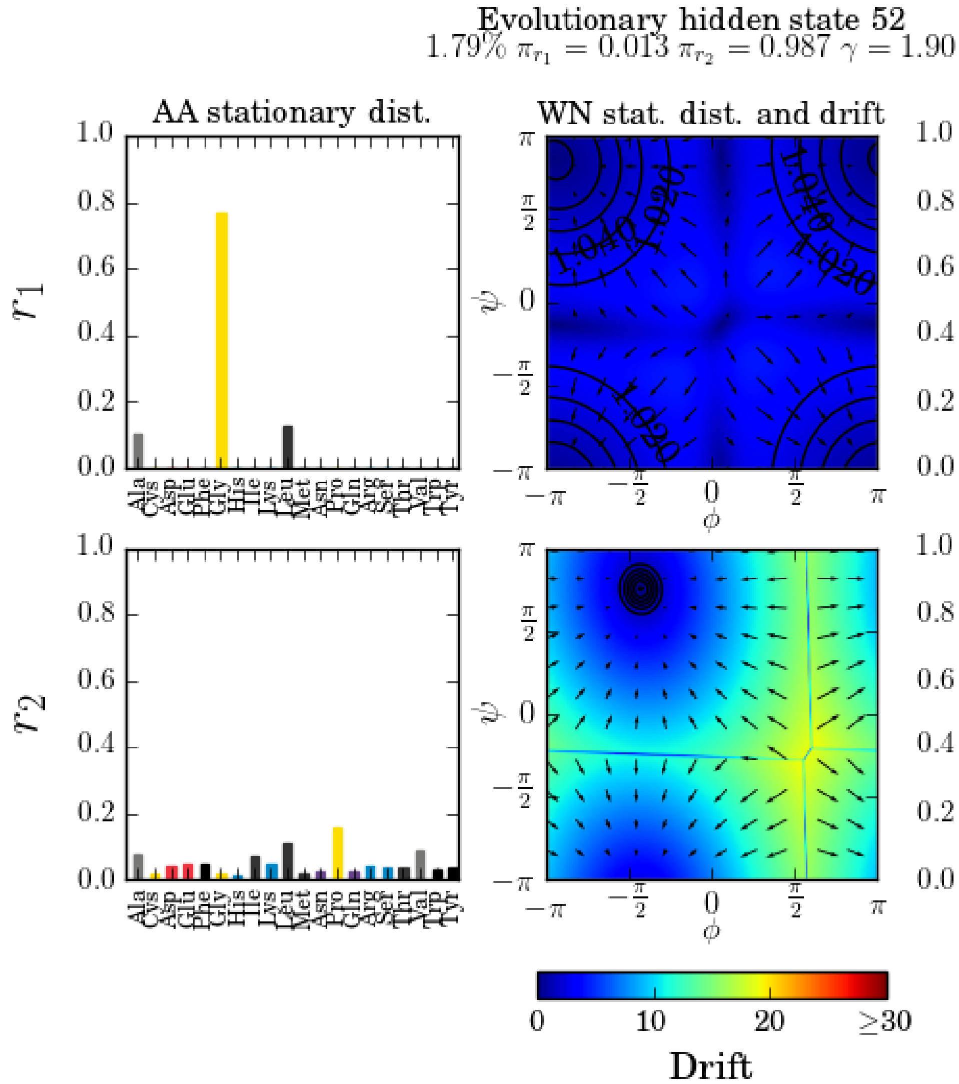

Evolutionary hidden state 52
1.79% $\pi_{r_1} = 0.013$ $\pi_{r_2} = 0.987$ $\gamma = 1.90$
AA stationary dist.
WN stat. dist. and drift
SS stationary dist.
r_1
r_2
Ala Cys Asp Glu Phe Gly His Ile Lys Leu Met Asn Pro Gln Arg Ser Thr Val Trp Tyr
H S C
ϕ
ψ
1.040
1.020
Drift
0 10 20 ≥30

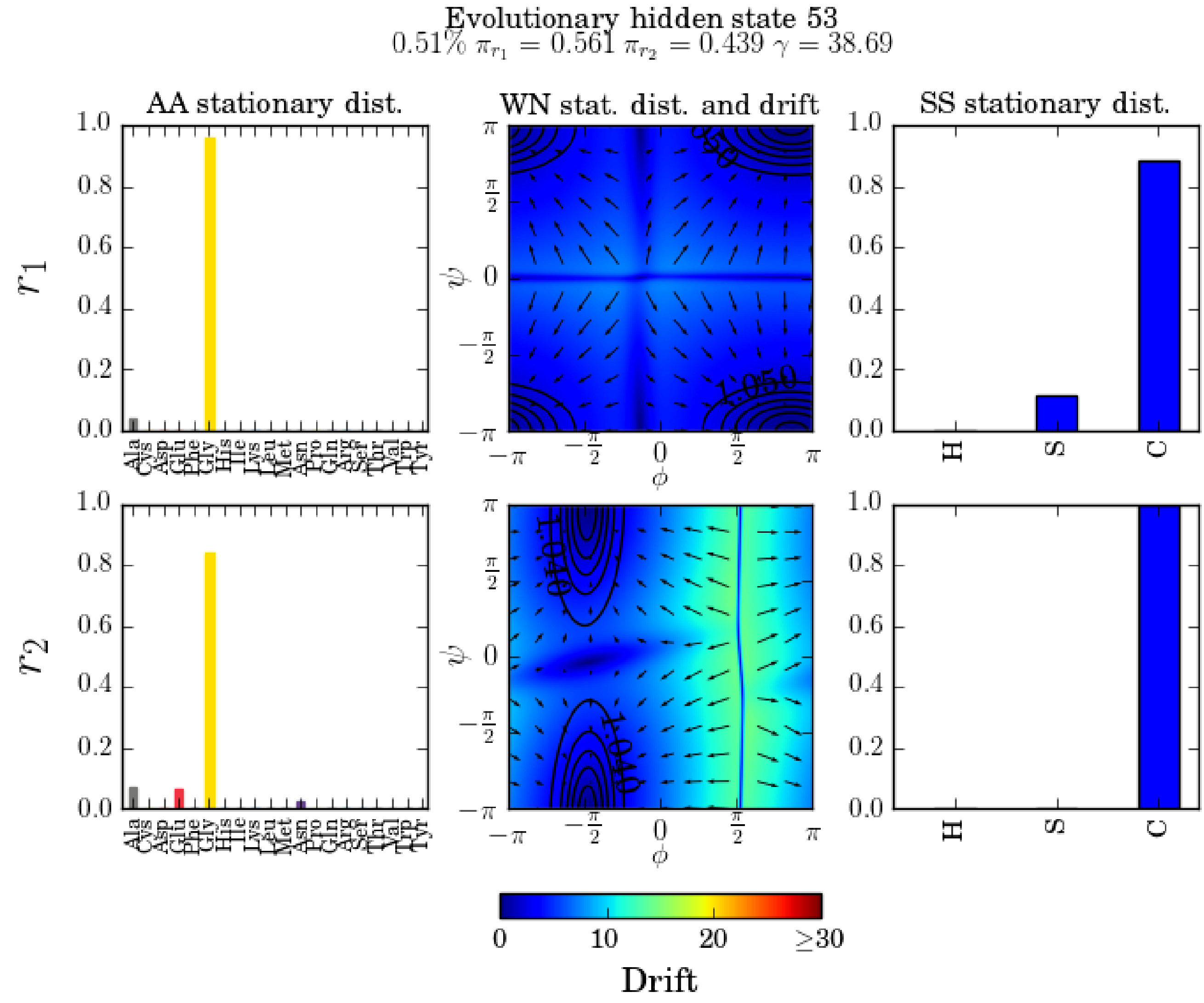

Evolutionary hidden state 53
$0.51\%\ \pi_{r_1} = 0.561\ \pi_{r_2} = 0.439\ \gamma = 38.69$
AA stationary dist.
WN stat. dist. and drift
SS stationary dist.
r_1
r_2
Ala Cys Asp Glu Phe Gly His Ile Lys Leu Met Asn Pro Gln Arg Ser Thr Val Trp Tyr
H S C
ψ
ϕ
1.050
1.040
Drift
0 10 20 ≥30

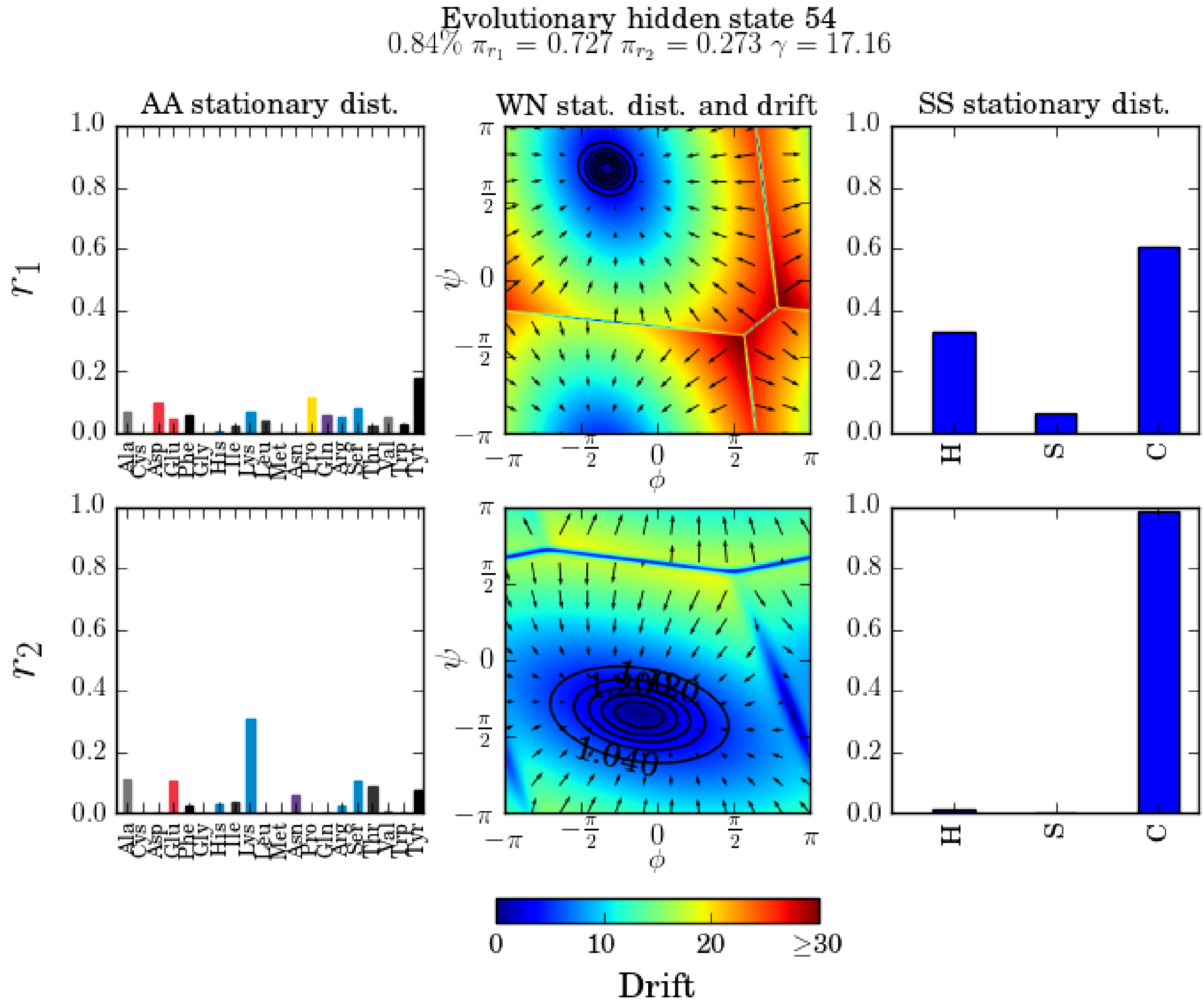
Evolutionary hidden state 54
0.84% $\pi_{r_1} = 0.727$ $\pi_{r_2} = 0.273$ $\gamma = 17.16$
AA stationary dist.
WN stat. dist. and drift
SS stationary dist.
r_1
r_2
Ala Cys Asp Glu Phe Gly His Ile Lys Leu Met Asn Pro Gln Arg Ser Thr Val Trp Tyr
H S C
ϕ
ψ
1.040
Drift

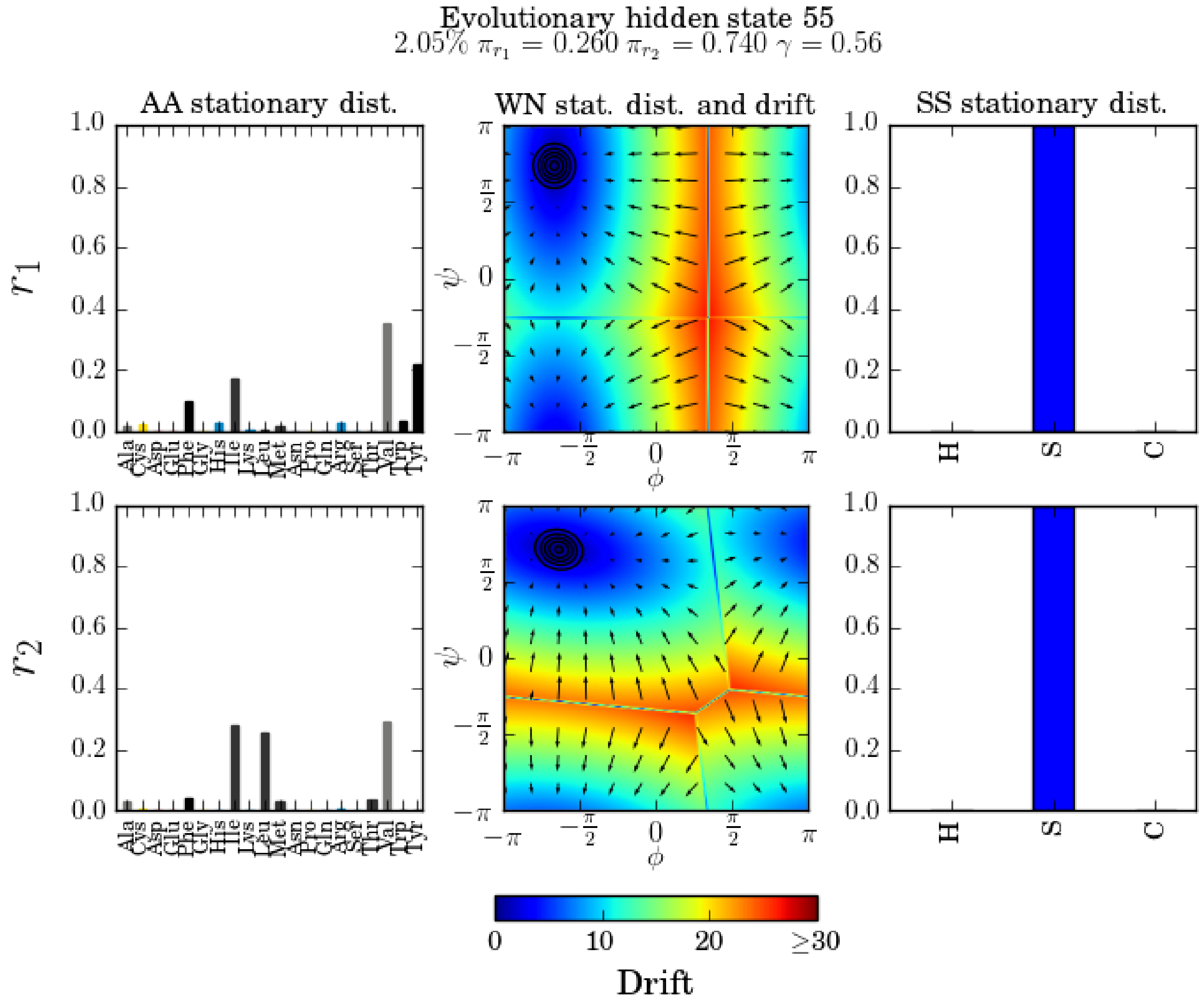
Evolutionary hidden state 55
2.05% $\pi_{r_1} = 0.260$ $\pi_{r_2} = 0.740$ $\gamma = 0.56$
AA stationary dist.
WN stat. dist. and drift
SS stationary dist.
r_1
r_2
1.0
0.8
0.6
0.4
0.2
0.0
Ala
Cys
Asp
Glu
Phe
Gly
His
Ile
Lys
Leu
Met
Asn
Pro
Gln
Arg
Ser
Thr
Val
Trp
Tyr
ψ
ϕ
$-\pi$
$-\frac{\pi}{2}$
0
$\frac{\pi}{2}$
π
H
S
C
0
10
20
≥30
Drift

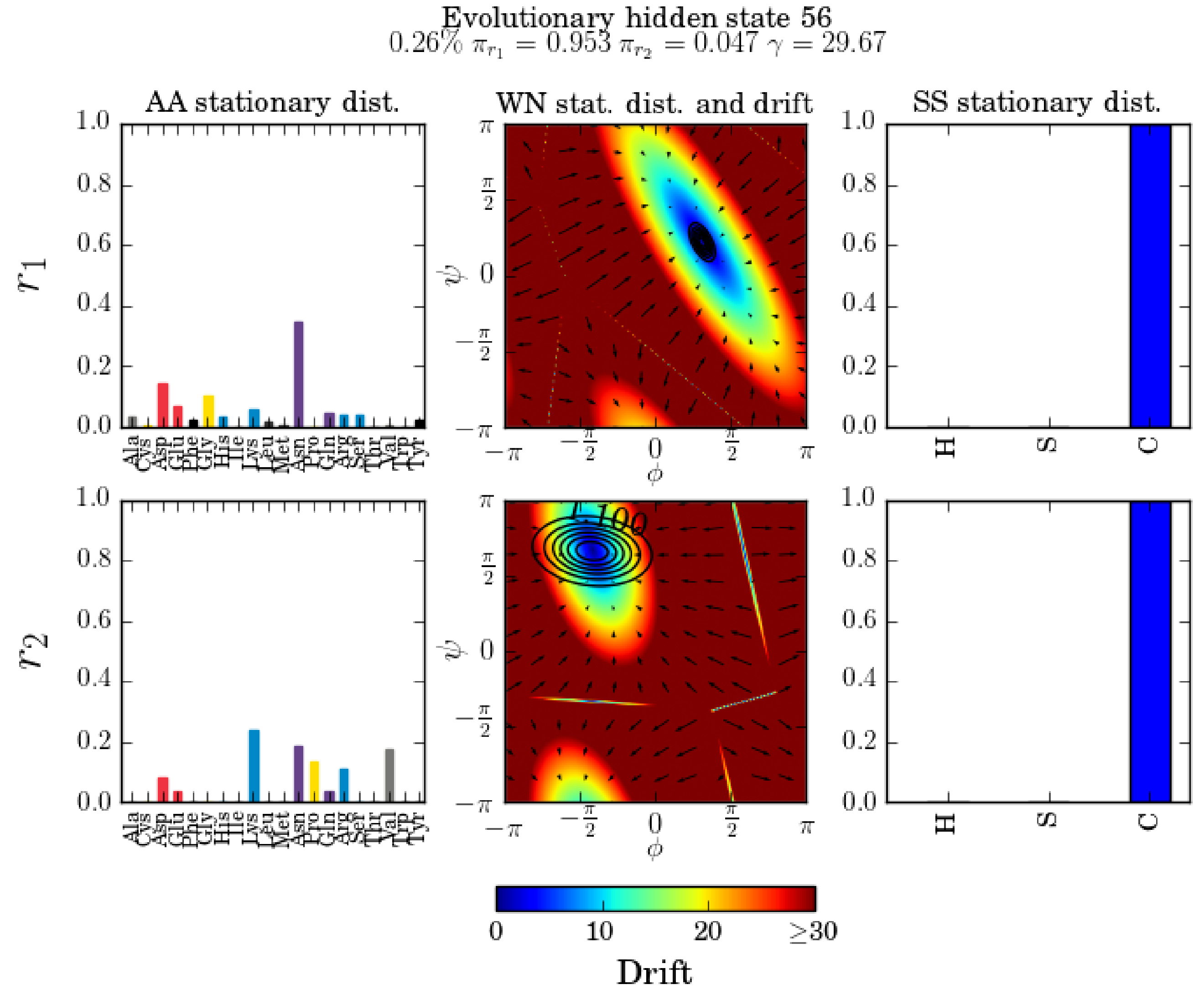
Evolutionary hidden state 56
0.26% $\pi_{r_1} = 0.953$ $\pi_{r_2} = 0.047$ $\gamma = 29.67$
AA stationary dist.
WN stat. dist. and drift
SS stationary dist.
r_1
r_2
ψ
ϕ
Ala Cys Asp Glu Phe Gly His Ile Lys Leu Met Asn Pro Gln Arg Ser Thr Val Trp Tyr
H S C
1.0 0.8 0.6 0.4 0.2 0.0
$-\pi$ $-\frac{\pi}{2}$ 0 $\frac{\pi}{2}$ π
0.100
0 10 20 ≥30
Drift

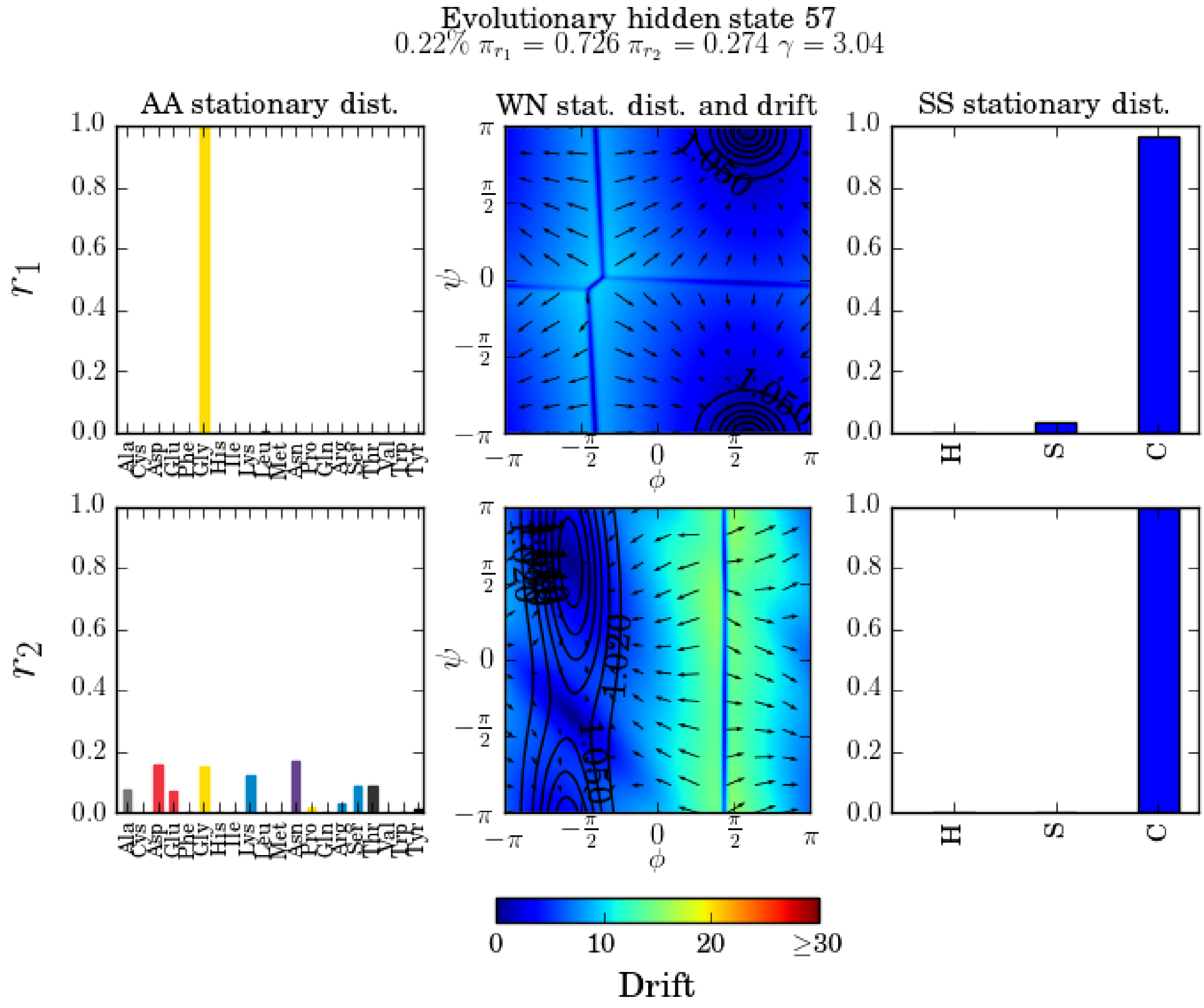
Evolutionary hidden state 57
0.22% $\pi_{r_1} = 0.726$ $\pi_{r_2} = 0.274$ $\gamma = 3.04$
AA stationary dist.
WN stat. dist. and drift
SS stationary dist.
r_1
r_2
Ala Cys Asp Glu Phe Gly His Ile Lys Leu Met Asn Pro Gln Arg Ser Thr Val Trp Tyr
H S C
ϕ
ψ
1.050
1.020
Drift
0 10 20 ≥30

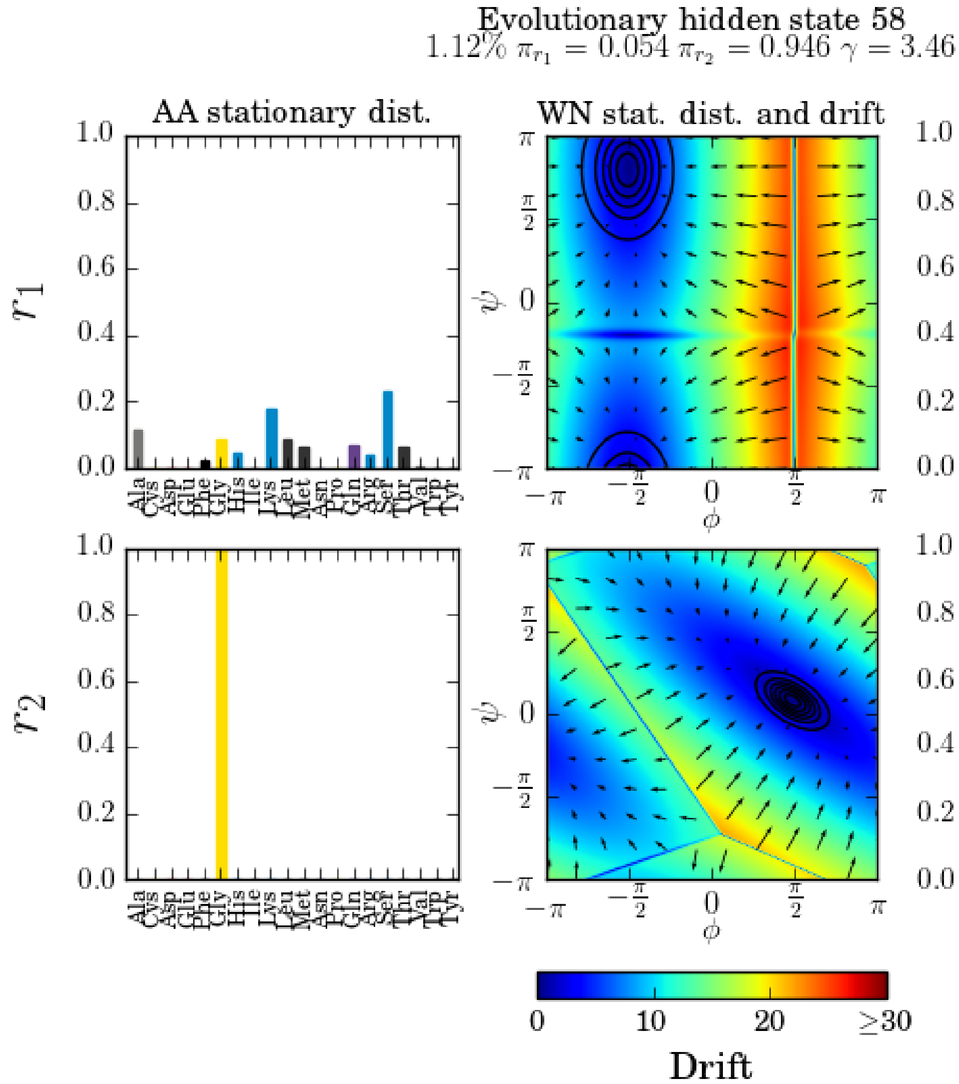

Evolutionary hidden state 58
1.12% $\pi_{r_1} = 0.054$ $\pi_{r_2} = 0.946$ $\gamma = 3.46$
AA stationary dist.
WN stat. dist. and drift
SS stationary dist.
r_1
r_2
Ala Cys Asp Glu Phe Gly His Ile Lys Leu Met Asn Pro Gln Arg Ser Thr Val Trp Tyr
H S C
ψ
ϕ
Drift
0 10 20 ≥30

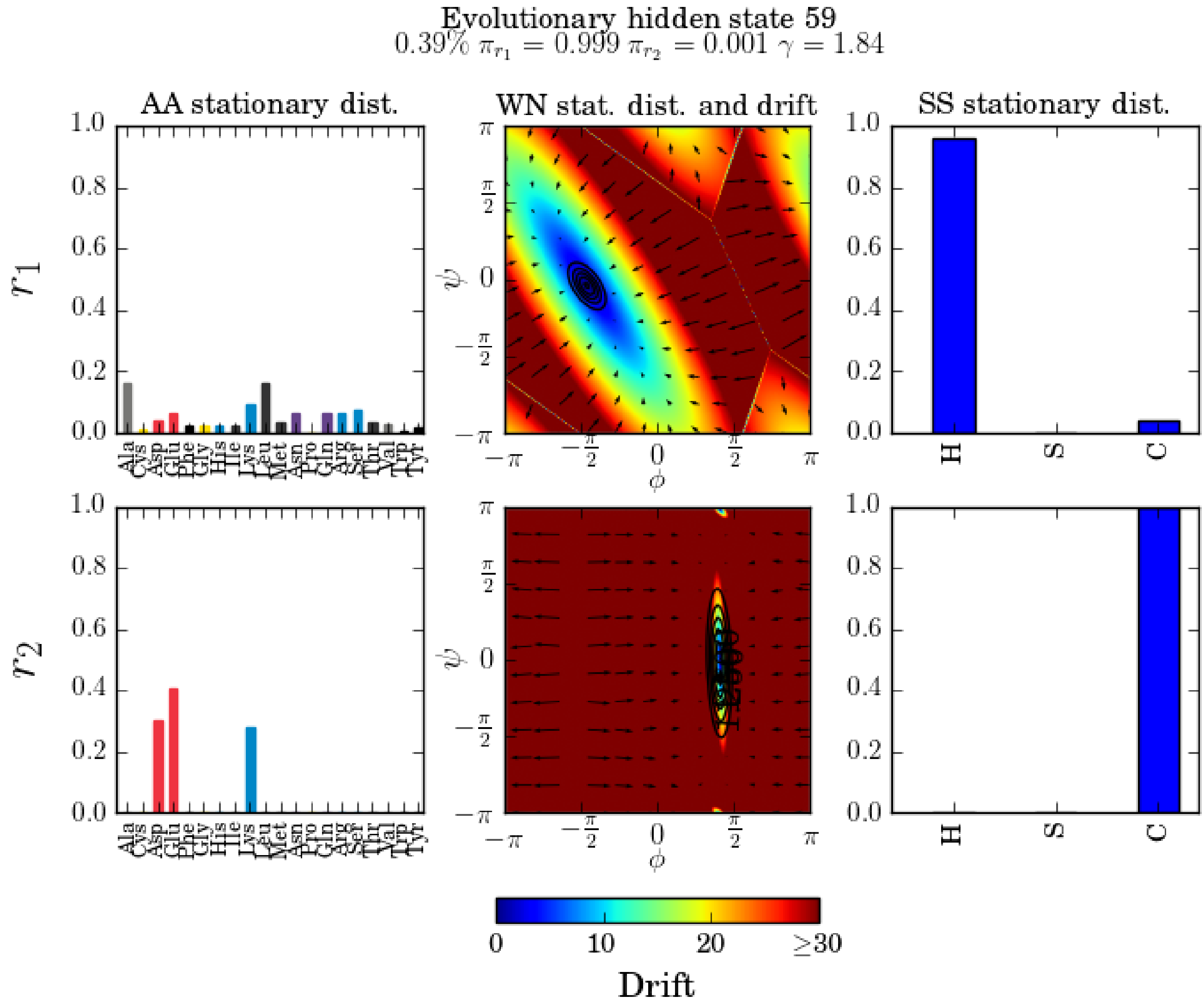

Evolutionary hidden state 59
0.39% $\pi_{r_1} = 0.999$ $\pi_{r_2} = 0.001$ $\gamma = 1.84$
AA stationary dist.
WN stat. dist. and drift
SS stationary dist.
r_1
r_2
Ala Cys Asp Glu Phe Gly His Ile Lys Leu Met Asn Pro Gln Arg Ser Thr Val Trp Tyr
H S C
ϕ
ψ
Drift
0 10 20 ≥30

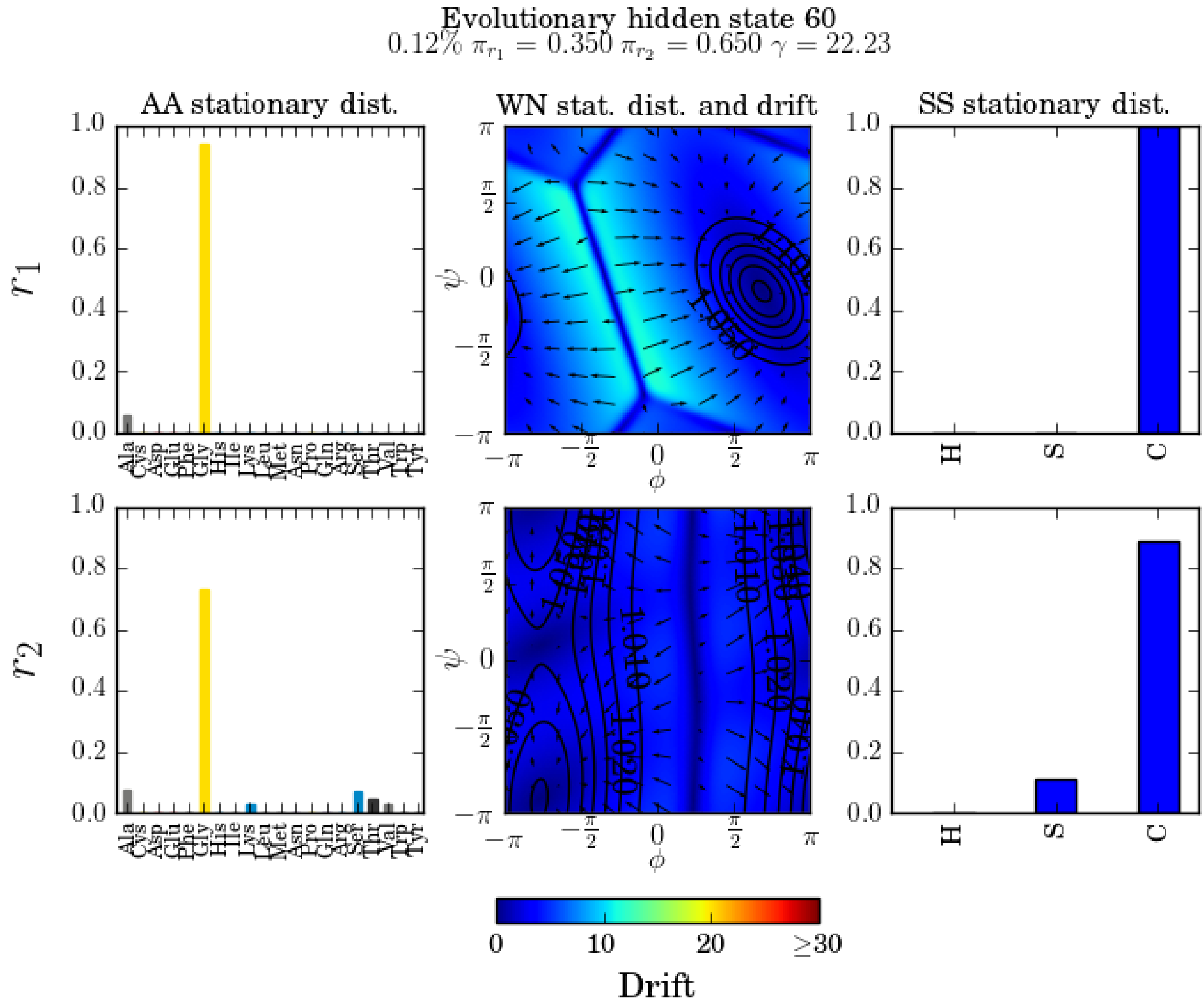

Evolutionary hidden state 60
0.12% $\pi_{r_1} = 0.350$ $\pi_{r_2} = 0.650$ $\gamma = 22.23$
AA stationary dist.
WN stat. dist. and drift
SS stationary dist.
r_1
r_2
Ala Cys Asp Glu Phe Gly His Ile Lys Leu Met Asn Pro Gln Arg Ser Thr Val Trp Tyr
H S C
ψ
ϕ
Drift
0 10 20 ≥30

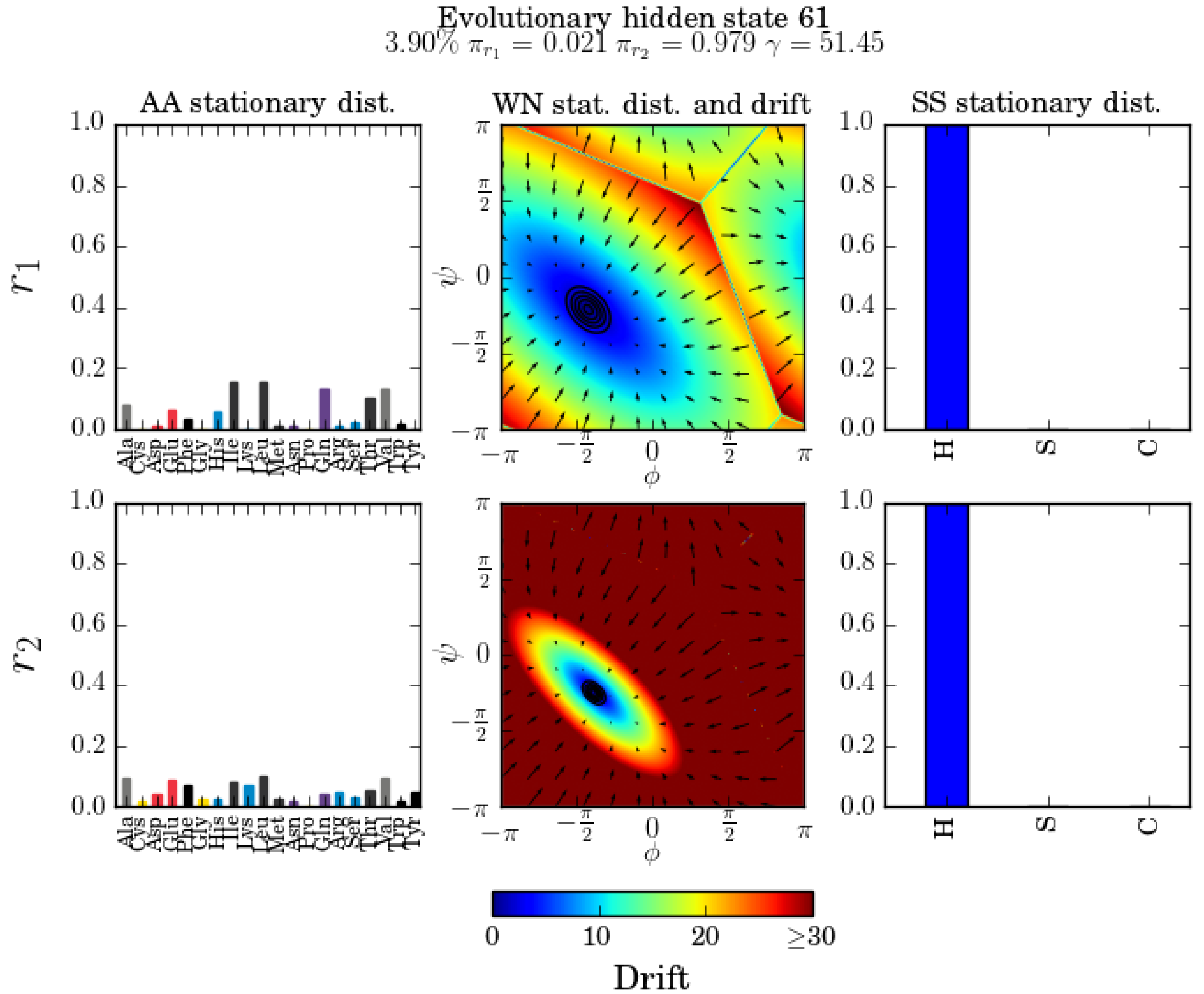

Evolutionary hidden state 61
3.90% $\pi_{r_1} = 0.021$ $\pi_{r_2} = 0.979$ $\gamma = 51.45$
AA stationary dist.
WN stat. dist. and drift
SS stationary dist.
r_1
r_2
Ala Cys Asp Glu Phe Gly His Ile Lys Leu Met Asn Pro Gln Arg Ser Thr Val Trp Tyr
ϕ
ψ
H S C
Drift
0 10 20 ≥30

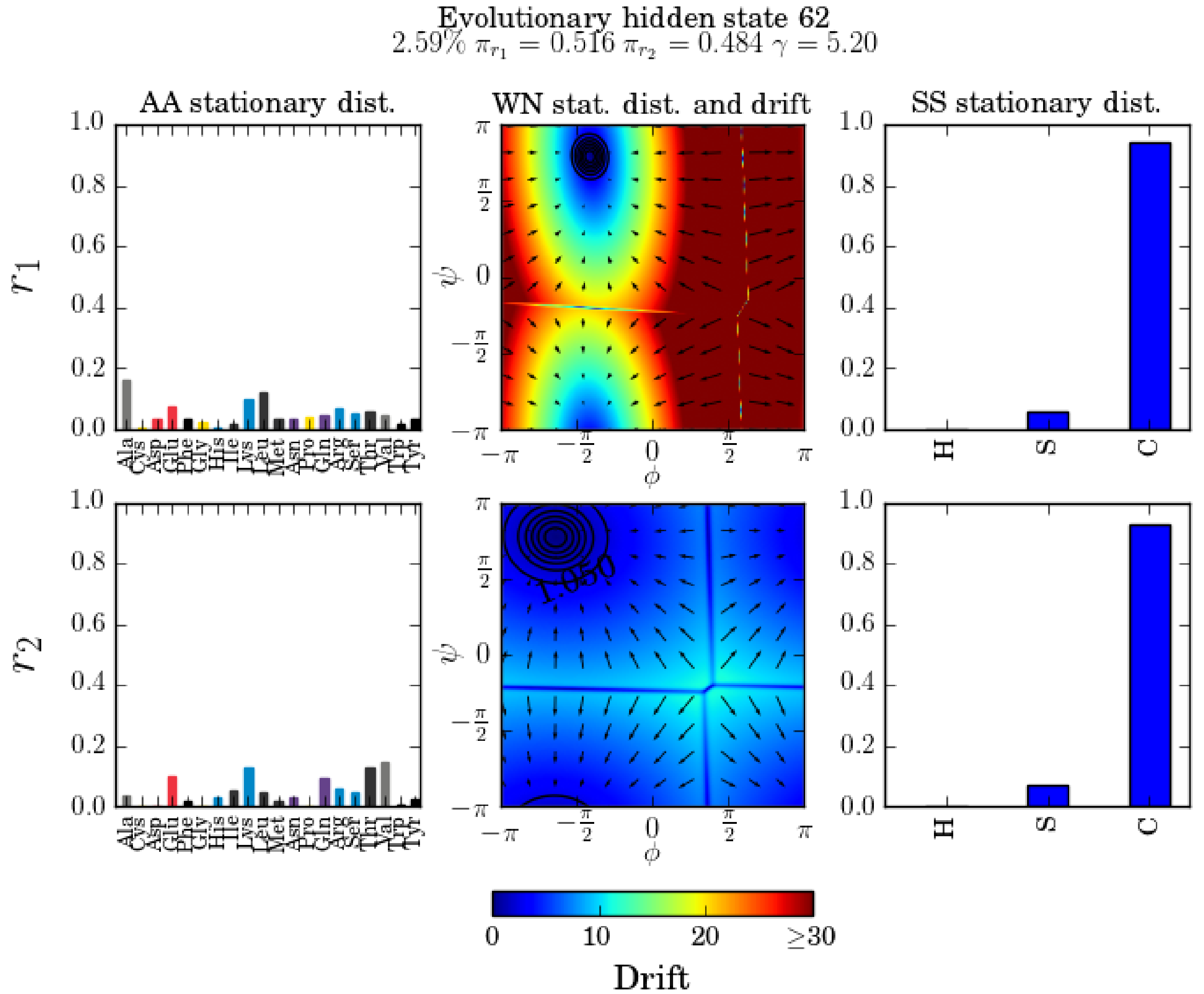
Evolutionary hidden state 62
2.59% $\pi_{r_1} = 0.516$ $\pi_{r_2} = 0.484$ $\gamma = 5.20$
AA stationary dist.
WN stat. dist. and drift
SS stationary dist.
r_1
r_2
Ala Cys Asp Glu Phe Gly His Ile Lys Leu Met Asn Pro Gln Arg Ser Thr Val Trp Tyr
H S C
ϕ
ψ
1.050
Drift
0 10 20 ≥30

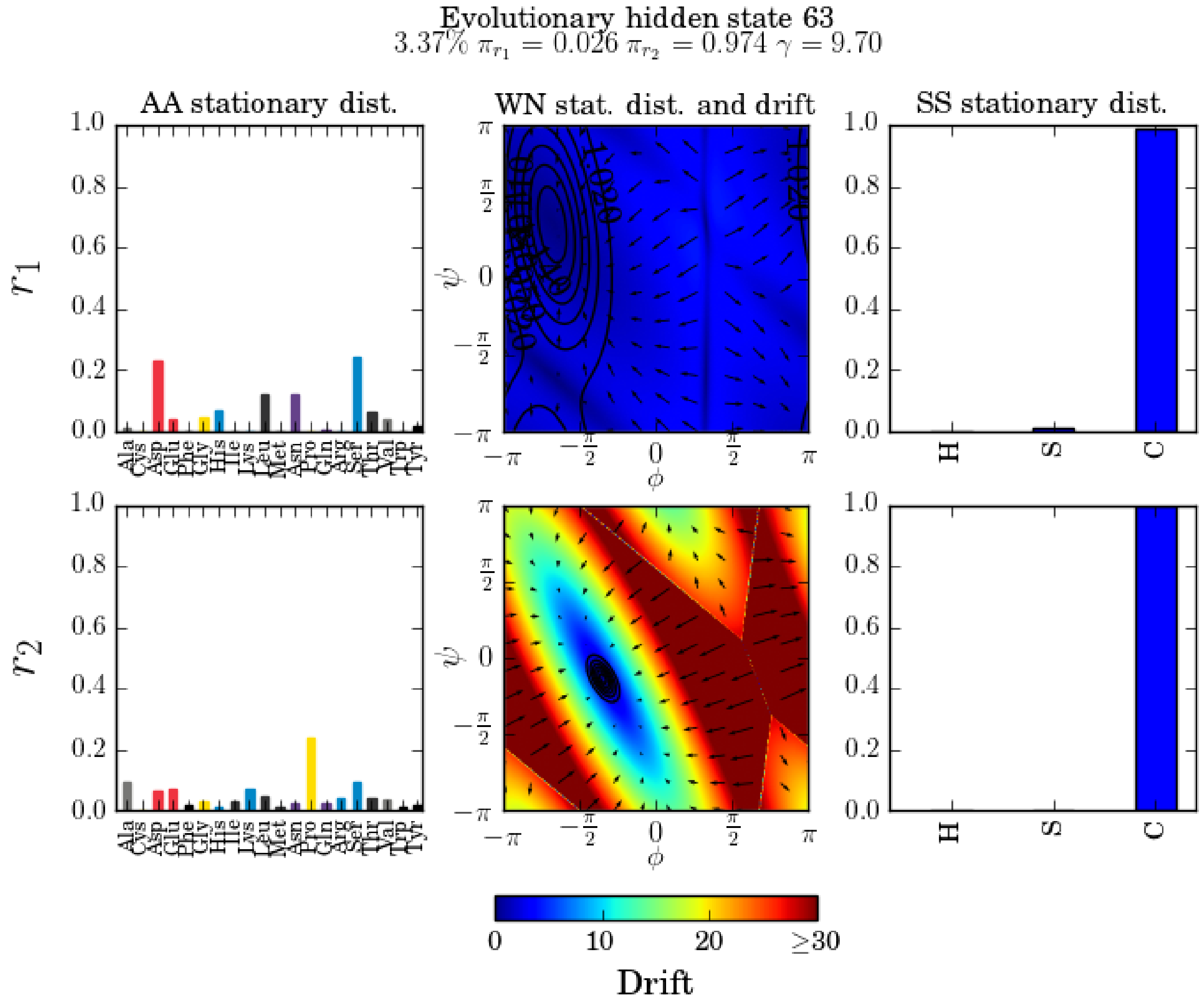

Evolutionary hidden state 63
3.37% $\pi_{r_1} = 0.026$ $\pi_{r_2} = 0.974$ $\gamma = 9.70$
AA stationary dist.
WN stat. dist. and drift
SS stationary dist.
r_1
r_2
Ala Cys Asp Glu Phe Gly His Ile Lys Leu Met Asn Pro Gln Arg Ser Thr Val Trp Tyr
ϕ
ψ
H
S
C
Drift

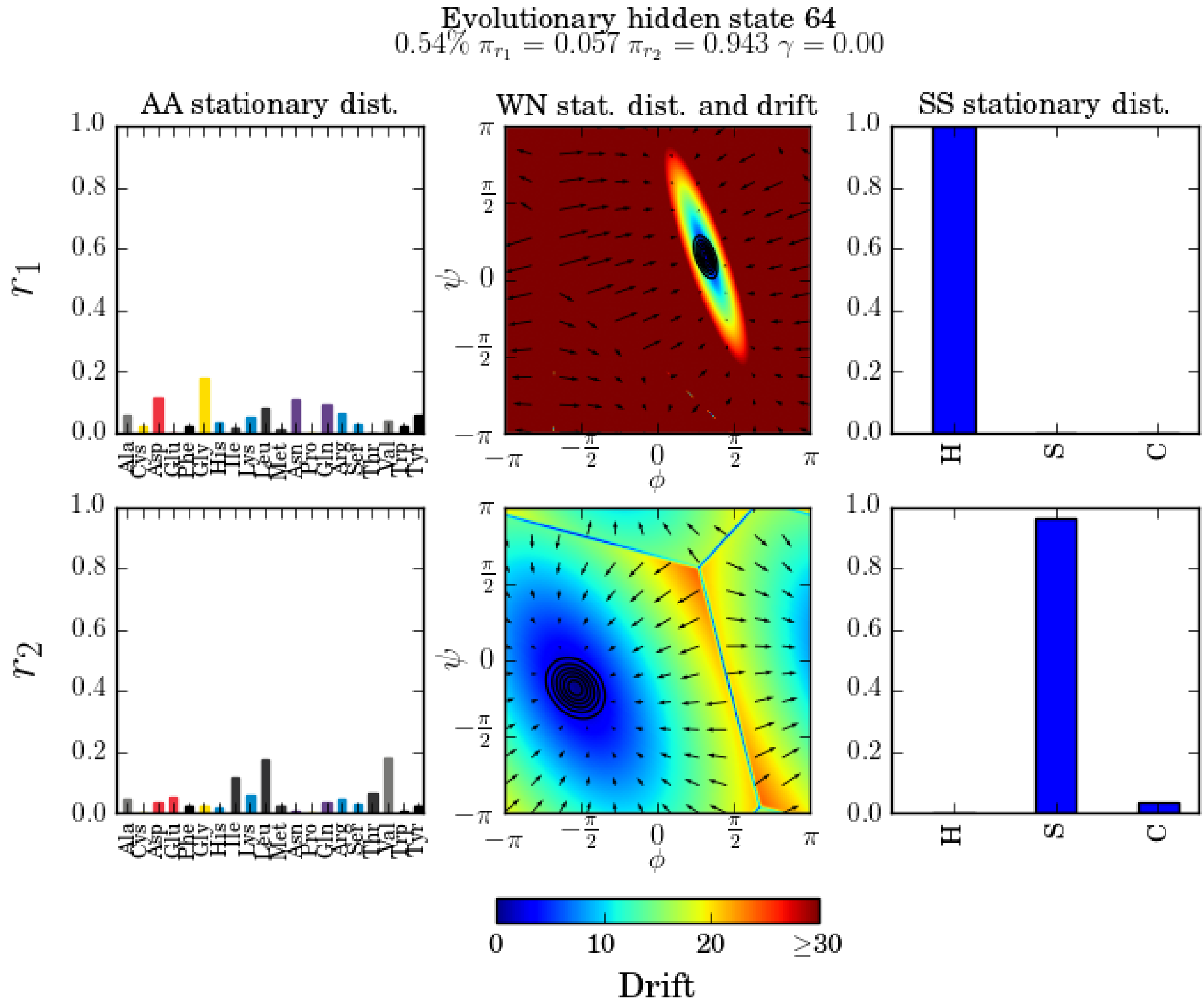

Evolutionary hidden state 64
0.54% $\pi_{r_1} = 0.057$ $\pi_{r_2} = 0.943$ $\gamma = 0.00$
AA stationary dist.
WN stat. dist. and drift
SS stationary dist.
r_1
r_2
Ala Cys Asp Glu Phe Gly His Ile Lys Leu Met Asn Pro Gln Arg Ser Thr Val Trp Tyr
ϕ
ψ
H S C
Drift
0 10 20 ≥30
