# Supplementary material for: A Generative Angular Model of Protein Structure Evolution
Source: Mol Biol Evol. 2017 Apr 27;34(8):2085–100. doi: 10.1093/molbev/msx137 (PMC5850488; doi:10.1093/molbev/msx137)
Supplement: Supplementary Data [file msx137_Supp.zip › supplementary-revision.pdf]

Supplementary material

1 Supplementary methods

1.1 Computation of transition probability density

The computationally tractable nature of the stationary density and tpd (equation 6 in the main text) required is key to enabling efficient training of and sampling under the model. Specifically, the computation of (equation 5 in the main text) involves evaluating e^{-tA} and Γ_t, which we can work out explicitly. First, $e^{-tA} = a(t)I - b(t)A$ with $a(t) = e^{-rt}(\cosh(qt) + r\frac{\sinh(qt)}{q})$, $b(t) = e^{-rt}\frac{\sinh(qt)}{q}$, $r = \frac{\mathrm{tr}(A)}{2}$ and $q = \sqrt{|\det(A - rI)|}$. Second, since $A^{-1}\Sigma$ is symmetric,

$$\Gamma_t = s(t)\tfrac{1}{2}A^{-1}\Sigma + i(t)\Sigma,$$

with $s(t) = 1 - a(2t)$ and $i(t) = \frac{b(2t)}{2}$. This gives a neat interpolation of the stationary and infinitesimal covariance matrices, particularly convenient for efficiently evaluating (equation 5 in the main text) at different t's.

1.2 Construction of test and training datasets

For each protein family in HOMSTRAD (ranging in size from 2 to 22 homologous proteins each), a phylogenetic tree was inferred from the HOMSTRAD protein family sequence alignment using FastTree (Price *et al.* (2010)). Each protein family tree was taken and protein pairs selected such that the sum of the branches between pairs was maximised, whilst ensuring that no pair of proteins in the set shared an overlapping evolutionary history. This was done in order to maximise the amount of information, whilst minimising dependencies due to shared evolutionary history.

Dihedral angles were computed from the PDB coordinates of each protein structure using the BioPython.PDB package (Hamelryck and Manderick (2003). Furthermore, each protein structure was obtained from the PDB

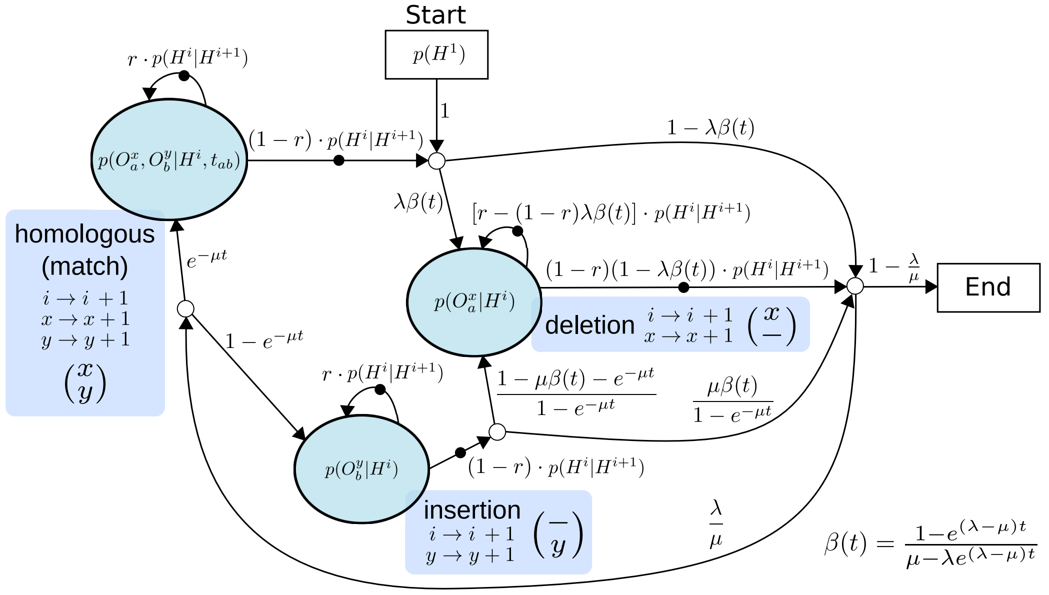

Figure 1: Diagrammatic representation of modified TKF92 alignment with neighbour-dependent evolutionary hidden states. The small white nodes represent non-emitting nodes. The three large oval nodes represent emitting nodes, emitting a deletion with respect to the first sequence $\binom{x}{-}$, a insertion with respect to the first sequence $\binom{-}{y}$ or a homologous pair of amino acids $\binom{x}{y}$. Edges with filled black circles indicate that the evolutionary hidden state is permitted to transition to a potentially different evolutionary hidden state, such transitions are permissible on all edges which exit one of the three emitting states. Parameters determining the transition probabilities are as follows: the insertion rate ($\lambda > 0$), the deletion rate ($\mu > 0$, the extension rate ($0 < r < 1$) and the evolutionary time t_{ab}. It is required that $\lambda < \mu$, such that the distribution ($t \to \infty$) of sequence lengths is finite at stationarity. The evolutionary hidden state transition probabilities, $P(H^i|H^{i-1})$, are given by a $q \times q$ transition probability matrix and the initial probabilities at the first alignment site, $p(H^i)$, is given by a length q probability vector.

database. The secondary structure was annotated at each amino acid position using DSSP (Touw *et al.* (2015)). In the case of gaps (missing residues), the fragments were treated as separate proteins without secondary structure annotation, in order to avoid misclassification by DSSP. In these cases, the secondary structure annotations were thus treated as latent variables. All 38 proteins in the test dataset were free of gaps and were from distinct protein families.

1.3 Statistical alignment: modelling insertions and deletions with neighbouring dependencies

Protein sequences can not only undergo point mutation events, but also insertion and deletion (indel) events. We describe a modified pairwise TKF92 alignment HMM that models both local sequence/structure evolution and sequence alignment based on (Miklós *et al.* (2004)).

Whilst it is possible to fix the alignment in advance by pre-aligning the sequences using one of the many available optimisation-based alignment methods (Edgar (2004); Katoh *et al.* (2002)) or using a curated alignment (such as from the HOMSTRAD database), doing so ignores alignment uncertainty.

An alignment can be thought of as a statement about homology, such that when amino acid positions are aligned in order to indicate homology they are considered to have evolved solely via mutation along the evolutionary trajectory linking them and therefore not via an indel. As the evolutionary trajectory of indels is rarely observed in practice, it is difficult to make statements about the true underlying alignment (homology relationships), especially when the compared sequences are distantly related and/or the rate indel of evolution is high. The TKF92 model (Thorne *et al.* (1992)) gives a suitable distribution describing indel evolution.

For the pairwise case, the TKF92 model can be represented as an HMM using the formulation described in Miklós et al. (2008). This HMM formulation allows one to sum over all possible pairwise alignments in $\mathcal{O}(nm)$ time using the HMM forward-backward algorithm, where n and m are the respective lengths of the two sequences. Thereby accounting for alignment uncertainty due to insertions or deletions.

We implemented a modified version of this HMM implementation (Figure 1) such that each emitted pair of characters is drawn from one of q evolutionary hidden states (Figure 1). Additionally, we encode neighbour-

ing dependencies amongst evolutionary hidden states along the alignment, by specifying a probability transition matrix $p(H^i, H^{i+1})$, that allows the hidden states to transition at Insertion, Deletion or Match nodes. These hidden states are intended to encode local sequence and structure evolution. The introduction of evolutionary hidden states with neighbouring dependencies increases the computational complexity from $\mathcal{O}(nm)$ in a model without evolutionary hidden states to $\mathcal{O}(nmq^2)$ in a model with evolutionary hidden states, where q is the number of evolutionary hidden states. The likelihood of an observation pair under this model depends on the homology relationship, M^i_{ab}, at a given position is as follows:

$$p(O^i|M^i_{ab}, H^i, t_{ab}) = \tag{1}$$
$$\begin{cases} p(O_a^{x(i)}, O_b^{y(i)}|H^i, t_{ab}) & \text{if observations at positions} \\ & x(i) \text{ and } y(i) \text{ in proteins } a \text{ and } b, \\ & \text{respectively, are homologous.} \\ p(O_a^{x(i)}|H^i) & \text{if the observation at} \\ & \text{position x(i) in protein } a \\ & \text{is the result of an indel.} \\ p(O_b^{y(i)}|H^i) & \text{if the observation at} \\ & \text{position y(i) in protein } b \\ & \text{is the result of an indel.} \end{cases}$$

Where $M^i_{ab} \in \{\binom{x}{y}, \binom{x}{-}, \binom{-}{y}\}$ specifies one of three possible homology relationships at position i in the alignment (homologous amino acids, deletion with respect to protein a, and insertion with respect to protein a, respectively.). Where $x \in \{1, \ldots, |a|\}$ and $y \in \{1, \ldots, |b|\}$ specify the indices of the positions in proteins a and b, respectively. Note that a disadvantage of our approach is that neighbouring amino acid positions in the presence of a deletion or series of deletions are no longer treated as directly adjacent by our HMM with respect to that particular protein, whereas in physical reality the amino acid positions would be directly adjacent to one another. However, the original HMM formulation where we treat the alignment as given *a priori* and similar phylogenetics HMMs (Liò *et al.* (1998); Siepel and Haussler (2004)) have the same property.

1.4 Model training and selection

Training Stochastic EM (StEM, Gilks *et al.* (1995)) was used to trained the model. StEM is a stochastic version of the well known Expectation-Maximization algorithm (Gilks *et al.* (1995)). Its distinguishing feature is that the E-step consists of filling in the values of the latent variables using sampling. Only a single value is sampled. StEM is attractive due to its computational efficiency and its tendency to avoid getting stuck in local minima (Gilks *et al.* (1995))

Forward Filtering Backward Sampling (FFBS) was used in the E-step to jointly sample alignment configurations (M_{ab}), hidden node states (H) and site-classes, (r_a, r_b). The Metropolis-Hasting algorithm was used to sample the four pair-specific continuous parameters $\theta_{ab} = \{t_{ab}, \lambda_{ab}, \mu_{ab}, r_{ab}\}$: evolutionary time ($t_{ab}$), insertion ($\lambda_{ab}$), deletion ($\mu_{ab}$) and geometric-extension (r_{ab}) rates. In other words, at iteration k for each pair of unaligned observation sequences O_a and O_b we draw samples, from the following joint-distribution:

$$Z_{ab}^{(k)} \sim p(M_{ab}, H, r_a, r_b, \theta_{ab} | O_a, O_b, \Psi^{(k)}).$$

In the M-step the samples from the previous E-step, were used to update the hidden node parameters ($\hat{\Psi}$) using efficient sufficient statistics (ESSs). Where ESSs weren't used, the COBYLA optimization algorithm (Powell (1994)) in the NLOpt library (Johnson (2014)) was used to update the parameters.

Selection Models with 8, 16, 32, 48, 52, 56, 60, 64, 68, 72, 76, 80, 96 and 112 hidden states were trained until convergence for varying numbers of repetitions (2 to 4) using different initial random number seeds. The highest log-likelihood model of each repetition was selected for downstream analysis.

Following that, marginal likelihoods $p(D|\text{model})$ and corresponding Bayesian information criterion (BIC) scores were computed under each model by fixing the alignments to the respective HOMSTRAD alignments. The alignments were fixed *apriori* in order to make computation of the marginal likelihoods computationally tractable. Additionally, predictive accuracies under a homology modelling scenario, $p(X_b|A_a, A_b, X_a, \text{model})$, were calculated for each of the 38 protein pairs in the test dataset.

1.5 Parallelisation of model training

The StEM algorithm is trivially parallelised. In the E-step, the parameters, alignment and hidden states of protein pair proteins can be independently sampled in parallel, conditioned on the parameters, $\Psi^{(r)}$, from the M-step. Whereas, in the M-step the parameters corresponding to each hidden state can be independently updated in parallel conditioned on the samples from the E-step.

1.6 Calculation of angular distances

For benchmarking purposes, the angular cosine distance was used to measure distances between pairs of dihedral angles, $\langle\phi_a, \psi_a\rangle$ and $\langle\phi_b, \psi_b\rangle$. It is defined as follows (Downs and Mardia (2002)):

$$\begin{aligned} &d(\langle\phi_a, \psi_a\rangle, \langle\phi_b, \psi_b\rangle) \\ &= \sqrt{4 - 2\cos(\phi_a - \phi_b) - 2\cos(\psi_a - \psi_b)}. \end{aligned} \tag{2}$$

The maximum possible distance is $\sqrt{8} \approx 2.828$. It has the property that when $\phi_a - \phi_b \approx 0$ and $\psi_a - \psi_b \approx 0$ are near zero it may be approximated by the Euclidean distance – using the small angle approximation for cosine ($\cos\theta \approx 1 - \frac{\theta^2}{2}$ when θ is near zero):

$$\begin{aligned} &d(\langle\phi_a, \psi_a\rangle, \langle\phi_b, \psi_b\rangle) \\ &\approx \sqrt{4 - 2(1 - (\phi_a - \phi_b)^2/2) - 2(1 - (\psi_a - \psi_b)^2/2)} \\ &= \sqrt{(\phi_a - \phi_b)^2 + (\psi_a - \psi_b)^2}. \end{aligned}$$

1.7 Time-reversibility

The three stochastic processes are assumed to be time-reversible. This, together with assumption of time-reversibility in jumping between evolutionary site classes ensures overall time-reversibility. This allows us to treat the phylogenetic tree relating proteins p_a and p_b as unrooted, implying we can arbitrarily pick p_a or p_b as a root of the phylogenetic tree (Felsenstein (1981)). This avoids the need to marginalise over the common ancestor protein of proteins p_a and p_b. Note that whilst time-reversibility of the evolutionary processes at each site holds, this is different from reversibility of the HMM. The transition probability matrix of the HMM is not restricted to be reversible and therefore detailed-balance does not necessarily hold.

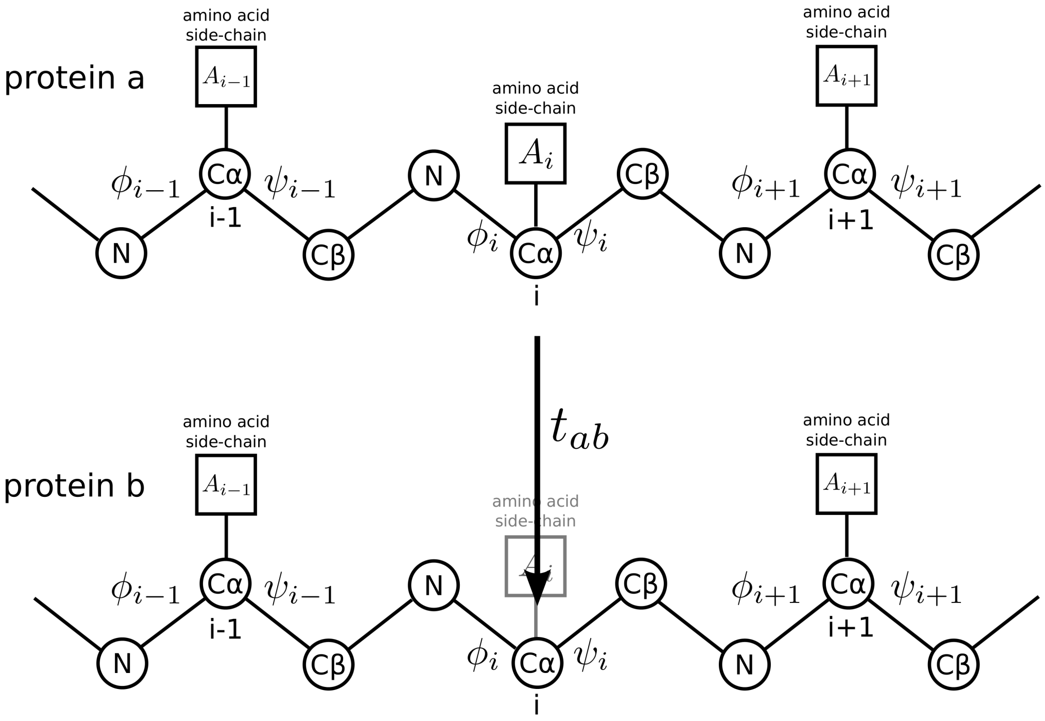

Figure 2: Time-reversibility allows an arbitrary rooting of the phylogeny. The stochastic processes describing the observations have all been chosen such that time-reversibility holds. This is particularly useful in the case of a pairwise phylogeny, because it permits us to arbitrarily pick one of the extant proteins (protein a or protein b) as the root of the phylogeny, without changing the likelihood of the data. This avoids computationally costly marginalisation of the unobserved common ancestor protein that in reality is shared by both of the extant proteins. Furthermore, only a single parameter, t_{ab}, the evolutionary time, need be marginalised when training the model or performing inference.

2 Supplementary results

2.1 Estimates of evolutionary time from dihedral angles are consistent with estimates from sequence

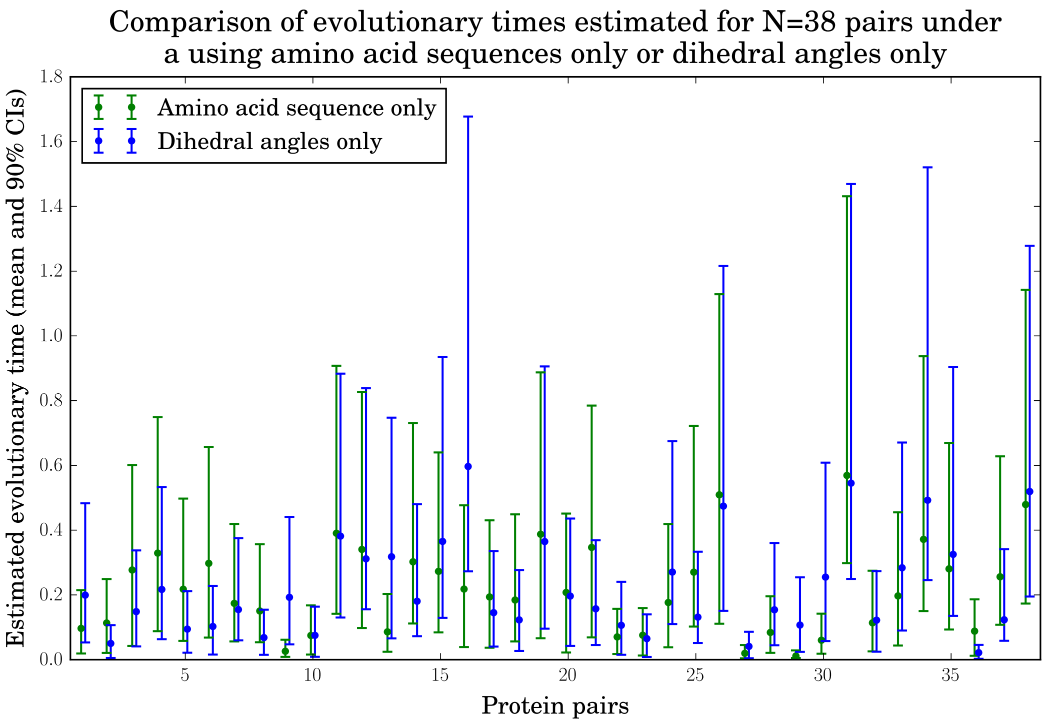

Figure 3: Evolutionary times were estimated for 38 protein pairs under a model with jump evolution under two different conditions. Under the first condition where only the amino acid sequences were treat as observed, whereas under the second only the dihedral angles were treated as observed.

Figure 3 compares evolutionary times estimated using pairs of homologous amino acid sequences only versus pairs of homologous dihedral angles only. The mean 90% confidence interval (CI) widths for the sampled evolutionary times were smaller for amino acid sequences (mean 90% CI width = 0.139) than dihedral angles ((mean 90% CI width = 0.191)

2.2 Exchangeability of secondary structure

Depicted below is the secondary structure exchangeability matrix V (see 'Secondary structure evolution' in the Methods section) inferred under the 64 hidden state model:

	**Helix**	**Sheet**	**Coil**
**Helix**	-19.82	0.02	19.80
**Sheet**	0.02	-0.67	0.65
**Coil**	19.80	0.65	-20.45

Comparing the magnitudes of the exchangeability rates shows that exchanges between a helix and a coil occur at a high rate (19.80), exchanges between a coil and sheet occur at an intermediate rate (0.65), and exchanges between a sheet and helix occur at a very low rate (0.02). The overall rate patterns suggest that in order for an evolutionary transition between a helix and a sheet to occur, a helix needs to first be unfolded into a coil, followed by a coil being folded into a sheet.

References

Downs, T. D. and Mardia, K. 2002. Circular regression. *Biometrika*, 89(3): 683–698.

Edgar, R. C. 2004. MUSCLE: multiple sequence alignment with high accuracy and high throughput. *Nucleic acids research*, 32(5): 1792–1797.

Felsenstein, J. 1981. Evolutionary trees from DNA sequences: a maximum likelihood approach. *Journal of molecular evolution*, 17(6): 368–376.

Gilks, W. R., Richardson, S., and Spiegelhalter, D. 1995. *Markov chain Monte Carlo in practice*. CRC press.

Hamelryck, T. and Manderick, B. 2003. Pdb file parser and structure class implemented in python. *Bioinformatics*, 19(17): 2308–2310.

Johnson, S. G. 2014. The NLopt nonlinear-optimization package.

Katoh, K., Misawa, K., Kuma, K.-i., and Miyata, T. 2002. Mafft: a novel method for rapid multiple sequence alignment based on fast fourier transform. *Nucleic acids research*, 30(14): 3059–3066.

Liò, P., Goldman, N., Thorne, J. L., and Jones, D. T. 1998. Passml: combining evolutionary inference and protein secondary structure prediction. *Bioinformatics*, 14(8): 726–733.

Miklós, I., Lunter, G., and Holmes, I. 2004. A long indel model for evolutionary sequence alignment. *Molecular Biology and Evolution*, 21(3): 529–540.

Powell, M. J. D. 1994. *A Direct Search Optimization Method That Models the Objective and Constraint Functions by Linear Interpolation*, pages 51–67. Springer Netherlands.

Price, M. N., Dehal, P. S., and Arkin, A. P. 2010. FastTree 2–approximately maximum-likelihood trees for large alignments. *PloS one*, 5(3): e9490.

Siepel, A. and Haussler, D. 2004. Combining phylogenetic and hidden markov models in biosequence analysis. *Journal of Computational Biology*, 11(2-3): 413–428.

Thorne, J. L., Kishino, H., and Felsenstein, J. 1992. Inching toward reality: an improved likelihood model of sequence evolution. *Journal of molecular evolution*, 34(1): 3–16.

Touw, W. G., Baakman, C., Black, J., te Beek, T. A., Krieger, E., Joosten, R. P., and Vriend, G. 2015. A series of PDB-related databanks for everyday needs. *Nucleic acids research*, 43(D1): D364–D368.
